# Integrated multi-omics analysis and machine learning identify hub genes and potential mechanisms of resistance to immunotherapy in gastric cancer

**DOI:** 10.18632/aging.205760

**Published:** 2024-04-22

**Authors:** Jinsong Wang, Jia Feng, Xinyi Chen, Yiming Weng, Tong Wang, Jiayan Wei, Yujie Zhan, Min Peng

**Affiliations:** 1 Department of Oncology, Renmin Hospital of Wuhan University, Wuhan 430060, Hubei, China

**Keywords:** gastric cancer, immunotherapy, machine learning, cancer-associated fibroblasts, prognosis

## Abstract

Background: Patients with gastric cancer respond poorly to immunotherapy. There are still unknowns about the biomarkers associated with immunotherapy sensitivity and their underlying molecular mechanisms.

Methods: Gene expression data for gastric cancer were gathered from TCGA and GEO databases. DEGs associated with immunotherapy response came from ICBatlas. KEGG and GO analyses investigated pathways. Hub genes identification employed multiple machine algorithms. Associations between hub genes and signaling pathways, disease genes, immune cell infiltration, drug sensitivity, and prognostic predictions were explored via multi-omics analysis. Hub gene expression was validated through HPA and CCLE. Multiple algorithms pinpointed Cancer-Associated Fibroblasts genes (CAFs), with ten machine-learning methods generating CAFs scores for prognosis. Model gene expression was validated at the single-cell level using the TISCH database.

Results: We identified 201 upregulated and 935 downregulated DEGs. Three hub genes, namely CDH6, EGFLAM, and RASGRF2, were unveiled. These genes are implicated in diverse disease-related signaling pathways. Additionally, they exhibited significant correlations with disease-associated gene expression, immune cell infiltration, and drug sensitivity. Exploration of the HPA and CCLE databases exposed substantial expression variations across patients and cell lines for these genes. Subsequently, we identified CAFs-associated genes and established a robust prognostic model. The analysis in the TISCH database showed that the genes in this model were highly expressed in CAFs.

Conclusions: The results unveil an association between CDH6, EGFLAM, and RASGRF2 and the immunotherapeutic response in gastric cancer. These genes hold potential as predictive biomarkers for gastric cancer immunotherapy resistance and prognostic assessment.

## INTRODUCTION

Gastric cancer is a prevalent global malignancy, carrying a significant burden of morbidity and mortality [1]. Worldwide, it ranks as the fourth leading cause of cancer-related death and the fifth most prevalent cancer [2]. In 2020 alone, there were over a million new cases of gastric cancer, with an estimated 700,000 deaths [2]. Notably, East Asia, particularly China, bears a substantial burden, with China accounting for around 44% of new global cases and half of the world’s fatalities [2]. The prevalent *Helicobacter pylori* infection can be blamed for the high prevalence of stomach cancer in China [3, 4]. Gastric cancer elusive etiology and delayed diagnosis contribute to its dismal survival rates [5]. Consequently, the imperative lies in the prevention, early detection, treatment efficacy, and prediction of sensitivity for gastric cancer.

Immune checkpoints are pivotal in orchestrating the host’s immune response, yet tumors exploit these checkpoints to evade immune eradication and simultaneously subdue immune reactions [6]. With the discovery of immune checkpoint pathways and ways to block them, targeted immunotherapy has become a major mutation in the treatment of solid tumors, bringing new hope to patients [7]. Since the first immunotherapeutic drug was applied to melanoma in 2011, immunotherapeutic drugs have evolved rapidly and become one of the most commonly used drugs in treating most tumors. However, the variability in response rates to such agents remains substantial. Notably, tumors boasting heightened somatic mutations, like melanoma and non-small cell lung cancer, tend to evince more favorable immunotherapeutic responses [8, 9]. Furthermore, patients with high PD1 and PD-L1 expression responded better to immunotherapy. Research has shown that PD-L1 is highly expressed in various solid tumors, including esophageal, colorectal, pancreatic, gastric, lung, and breast cancers [10]. Meanwhile, gastric cancer patients have a highly complex immune microenvironment as well as a high rate of somatic mutation [11, 12]. These studies suggest that gastric cancer patients may derive greater benefits from immunotherapy. However, the use of immunotherapy in gastric cancer has fallen short of expectations. It is only approved for use in advanced gastric cancer in some countries [13]. Therefore, identifying which gastric cancer patients are sensitive to immunotherapy or which are unsuitable for immunotherapy is crucial, and there is an urgent need for several biomarkers to aid in this identification.

In this investigation, we discerned genes intricately linked to immunotherapeutic response in gastric cancer, employing comprehensive genome-wide gene expression profiles from the TCGA and ICBatlas datasets. By employing diverse machine-learning techniques, we systematically evaluated potential biomarkers that hold promise for gauging the efficacy of immunotherapy in gastric cancer. Additionally, we pinpointed genes associated with CAFs and subsequently crafted a predictive model and a corresponding nomogram to forecast prognosis. Notably, the integrity of our model was reaffirmed through meticulous validation against pertinent data from the GEO repository.

## MATERIALS AND METHODS

### Acquisition of datasets related to gastric cancer

We sourced stomach adenocarcinoma (STAD) expression profiles, mutational data, and clinical records from the TCGA database (https://portal.gdc.cancer.gov/). The transcriptomic information for STAD encompassed a cohort of 448 samples, comprising 412 tumor tissues and 36 normal tissues. GSE15459 expression profiles and complete clinical information were obtained from the GEO database (https://www.ncbi.nlm.nih.gov/geo/). Extract differentially expressed genes (DEGs) associated with gastric cancer immunotherapy response from the ERP107734 data in the ICBatlas database (http://bioinfo.life.hust.edu.cn/ICBatlas/).

### Acquisition of DEGs and functional analysis

DEGs were obtained from the ICBatlas database, where genes with FDR (False Discovery Rate) < 0.05 and |log2FC (Log2 Fold Change)| > 1 were used for subsequent analysis. KEGG and GO analyses were conducted to determine the pathways and functions of DEGs enrichment associated with immunotherapy response, facilitated by the “clusterProfiler” software package. The Search Tool for the Retrieval of Interacting Genes (STRING) database obtained a protein-protein interaction (PPI) network graph with a minimum interaction score of 0.7 (http://string.embl.de/) [14].

### Identifying hub genes through machine learning

Firstly, the genes most relevant to the disease among the DEGs were identified in the TCGA transcript data by the “WGCNA” package [15]. Subsequently, using P<0.05 as the criterion, perform univariate Cox regression to screen for prognostic genes among the genes obtained from the Weighted gene co-expression network analysis (WGCNA) analysis. We used the “randomForestSRC” package and the “randomSurvivalForest” package to perform a random survival forest algorithm to rank the importance of prognostic genes. We selected genes with significance >0.4 for subsequent analyses. We used the “XGBoost”, and “Boruta” packages for the Boruta and Extreme Gradient Boosting (XGBoost) algorithms [16] for prognostic genes were ranked in terms of their importance. By using these three machine learning methods, the hub genes are ultimately determined.

### Gene set variation analysis (GSVA) of hub genes

GSVA [17] was performed using the R package “GSVA” to explore the relevance of core genes in the TCGA_STAD cohort to the Hallmark pathway. Relevant gene sets were downloaded from the Molecular Signature Database (MSigDB) (http://software.broadinstitute.org/gsea/msigdb/index.jsp).

### Gene set enrichment analysis (GSEA) of hub genes

In this study, differentially expressed genes between the high and low-expression groups were identified based on the expression profiles of STAD patients using the GSEA tool (http://www.broadinstitute.org/gsea) [18]. When screening gene sets, the maximum gene set size was set to 500 and the minimum gene set size to 15. After 1000 permutations, a significantly enriched gene set was obtained based on a significance level of P<0.05.

### Core gene mutation analysis

Mutational differences in somatic cells between high and low expression of core genes were investigated using TCGA somatic mutation data by the “maftools” package [19]. Mutations in core genes were studied through the cBioPortal database (https://www.cbioportal.org).

### Correlation analysis between core genes and disease-related genes

We searched the GeneCards database for STAD-associated disease genes using the keyword “STAD” and used the 20 genes with the highest relevance scores for subsequent analyses (https://www.genecards.org/). We performed Pearson correlation analysis between the expression levels of core genes and disease-related genes, where P<0.05 was considered statistically significant.

### Immune infiltration analysis

The transcript data from the TCGA_STAD cohort were analyzed using the cell-type identification by estimating relative subsets of RNA transcripts (CIBERSORT) algorithm [20] to calculate the relative content of 22 types of immune infiltrating cells and to analyze the difference between the immune cell content of normal and tumor tissues. We performed Pearson correlation analysis between the expression of core genes and the content of immune cells. The expression of core genes and immune-related genes were compared using Pearson correlation analysis after immune-related genes were also retrieved from the Tumor and Immune System Interaction Database (TISIDB) (http://cis.hku.hk/TISIDB/). P<0.05 was considered statistically significant.

### Drug sensitivity analyses

Downloaded the relevant data from the Genomics of Drug Sensitivity in Cancer (GDSC) database (https://www.cancerrxgene.org/), and used the R package “oncoPredict” to predict drug sensitivity for each tumor sample in order to evaluate the predictive ability of core genes on drug treatment response. Default values were selected for all parameters, including “combat” to eliminate batch effects and average duplicate gene expression. The IC_50_ differences between different expressions of the core genes were then compared using the Wilcoxon test. Finally, we plot the results using the “ggplot” R package.

### Validation of core gene expression in databases

Expression data of gastric cancer cell lines were downloaded from the CCLE (http://www.broadinstitute.<soft-enter/>org/ccle) database to validate the gene expression levels of core genes in gastric cancer cell lines. Immunohistochemistry-related data were downloaded from the HPA (http://www.proteinatlas.org) database to validate the protein expression levels of the core genes in normal and gastric cancer tissues.

### Identification of CAFs-related genes

Immunotherapy evaluation-related metrics were calculated using the TIDE (Tumor Immune Dysfunction and Exclusion) database, and the expression of core genes was analyzed for Pearson correlation with these metrics. At the same time, we assessed the tumor mutation burden (TMB) of each patient in the TCGA_STAD cohort. We performed Pearson correlation analysis between TMB and the expression levels of core genes.

CAFs content was calculated using three methods: the Estimated Proportion of Immune and Cancer Cells (EPIC) algorithm [21], the xCell algorithm [22], the Microenvironmental Cell Population-counter (MCP-counter) algorithm [23]. First, in the TCGA transcripts, we counted genes associated with the core genes, and genes with correlation coefficients >0.3 and p<0.05 were included in subsequent analyses. We then performed WGCNA analysis using the “WGCNA” package to obtain the CAFs genes associated with the core genes. Finally, we selected gene significance (GS) > 0.5 as potential CAFs-related genes.

### Enrichment analysis

Utilizing the “clusterProfiler” software package, we executed KEGG and GO analyses to unravel the pathways and functions enriched with CAFs-related genes. We also conducted Disease Ontology (DO) enrichment analysis using the R package “DOSE” [24] to identify which diseases are associated with CAFs-related genes.

### Construction and validation of CAFs features

The obtained CAFs-related genes were subjected to univariate Cox regression analysis, and genes with P<0.05 were considered prognostic-related genes. We used the prognostic-related genes obtained from the univariate Cox regression analysis and took the intersection with the genes from the GSE15459 dataset for further analysis. To build a CAFs signature with high accuracy and generalizability, we integrate ten machine learning algorithms, including Cox boost, Stepwise Cox, Lasso, Ridge, Elastic Net (Enet), Survival Support Vector Machines (survival-svm), Generalized Boosted Regression Models (GBMs), Supervised Principal Components (SuperPC), Partial Minimum Cox (plsRcox) and RSF. In constructing this model, we set “ntree” to 1000 and “nodesize” to 5. During the model selection process, we calculate each model’s consistency index (C-index) and filter based on a combination of the number of genes in the model and the C-index. Models with higher C-index and fewer genes were considered the optimal models.

The Kaplan-Meier method was employed to generate survival curves. At the same time, the log-rank test was utilized to assess the disparity in survival rates between the high- and low-risk cohorts. The models’ accuracy was evaluated using Receiver Operating Characteristic (ROC) curves. The model underwent univariate and multivariate Cox regression analyses to evaluate its potential as an independent prognostic marker for STAD patients. All results were validated in the GSE15459 dataset. A nomogram with a calibration plot was constructed using the “rms” package to predict the consistency between actual and predicted survival. A multivariable ROC curve was constructed to compare with other factors to validate the model and nomogram optimality. Evaluation of the clinical usefulness of model and nomogram by decision curves was done.

### Single-cell sequencing analysis

The genes in the CAFs risk scores and models were subjected to Pearson correlation analysis with CAFs marker genes in the previous literature to see whether the genes identified by machine learning and the constructed models were associated with CAFs marker genes reported by previous authors. We analyzed single-cell RNA-sequencing (scRNA-seq) data from STAD tissues (GSE167297) based on the TISCH database [25], which was used to identify whether genes in the model were highly expressed in CAFs.

### Statistical analysis

R software (version 4.2.1) was used for all statistical analyses. Wilcoxon tests were used for group comparisons. Prognostic-related genes were screened using univariate Cox regression analysis. The log-rank test and Kaplan-Meier analysis were employed to compare overall survival. All correlation tests were conducted using the Pearson correlation analysis method in the study. P<0.05 was considered statistically significant.

### Availability of data and materials

Our research examined datasets that are publicly accessible. These data are available here: GSE15459 cohort from GEO database (https://www.ncbi.nlm.<soft-enter/>nih.gov/geo/); TCGA_STAD from TCGA database (https://portal.gdc.cancer.gov/).

## RESULTS

### Screening for immunotherapy efficacy-related DEGs and functional enrichment of DEGs

We filtered out 1136 DEGs between gastric cancer samples with response and without response to immune therapy from the ICBatlas database using the criteria of FDR<0.05 and |log2FC|>1 (Supplementary Table 1). The number of up-regulated genes was 201, and the number of down-regulated genes was 935 in responders compared to non-responders. Supplementary Figure 1A represents the volcano plot, while Supplementary Figure 1B shows the heatmap of DEGs. To further investigate the functions and pathways of DEGs, we performed KEGG and GO enrichment analyses. The KEGG pathway enrichment results showed that DEGs were mainly enriched in cancer, PI3K-Akt, gastric cancer, and MAPK pathways (Supplementary Figure 1C). GO enrichment analysis showed that DEGs were mainly involved in functions such as extracellular matrix composition, basement membrane, and receptor-ligand activity (Supplementary Figure 1D). PPI network analysis revealed that most DEGs were tightly and intricately linked (Supplementary Figure 1E).

### Machine learning of DEGs and identification of three hub genes, CDH6, EGFLAM, and RASGRF2

**Figure 1:**
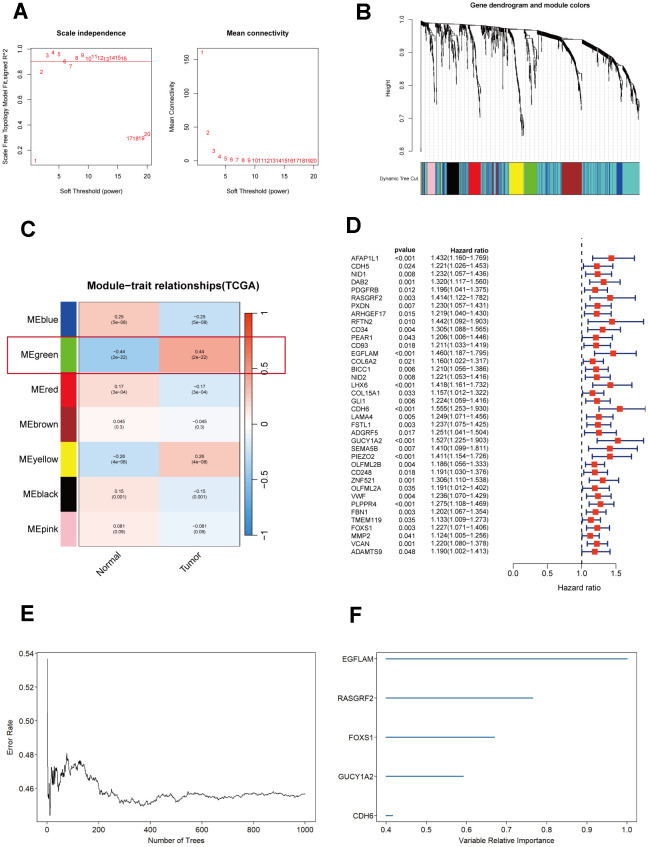
**Selection and analysis of differentially expressed genes in the TCGA_STAD cohort.** (**A**) Scale independence and mean connectivity plot generated using WGCNA. (**B**) Gene dendrogram and nodule color from WGCNA analysis. (**C**) Merged module correlation coefficients from WGCNA analysis. (**D**) Results of the univariate Cox regression analysis (P<0.05). (**E**) Random forest analysis of prognostic genes. (**F**) Ranking plot of selected features with random forest importance scores> 0.4.

**Figure 2:**
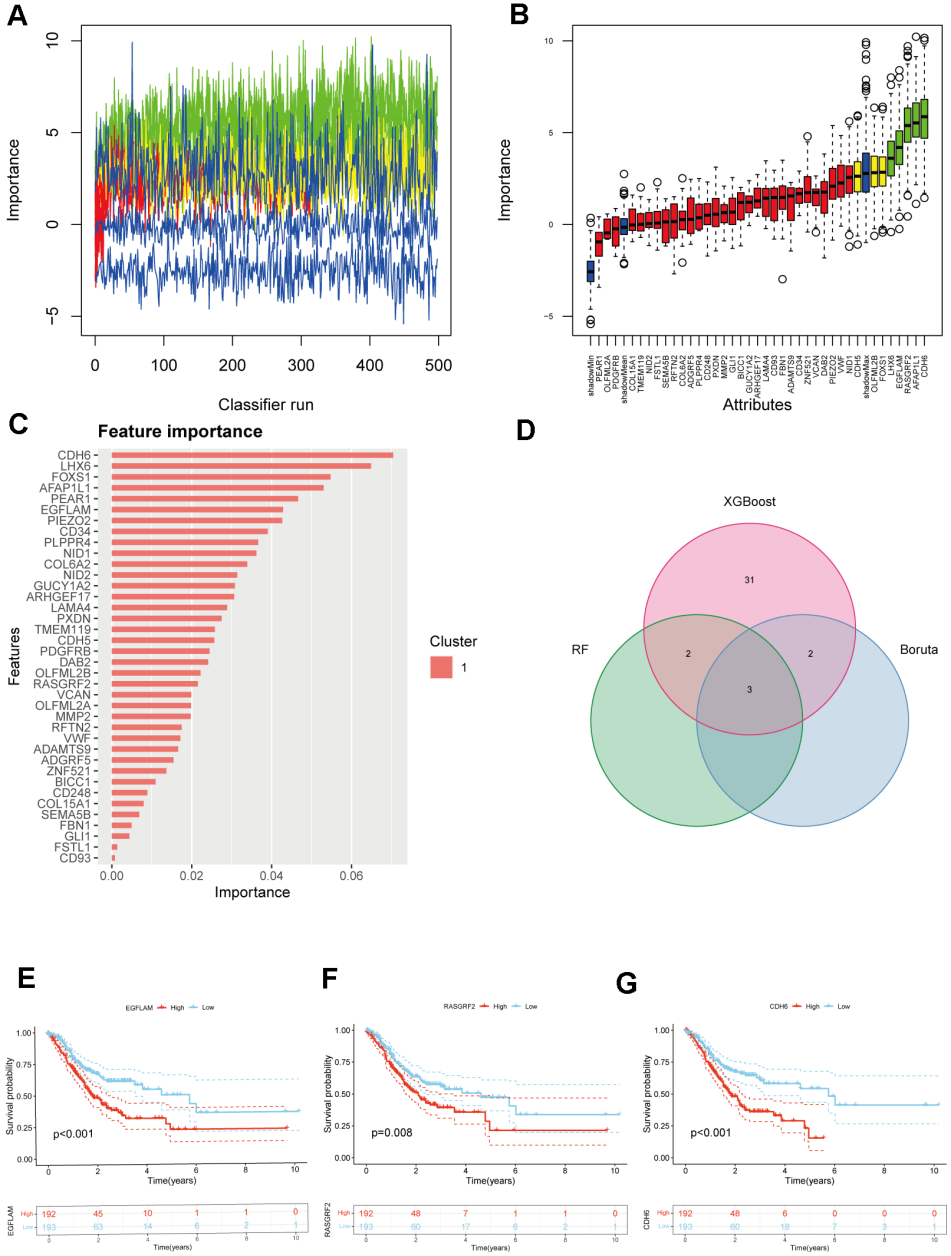
**Selection of hub genes from prognostic genes.** (**A**) Shadow feature plot for Boruta algorithm. (**B**) Confirmed plot for Boruta algorithm. (**C**) XGBoost analysis. (**D**) Venn diagram of hub genes identification using different methods. (**E**–**G**) Kaplan-Meier survival analysis of EGFLAM, RASGRF2, and CDH6 (p<0.001, p=0.008, and p<0.001, respectively).

To further understand which genes in DEGs affect immunotherapy, we selected the TCGA_STAD cohort for WGCNA analysis and screened the genes most relevant to STAD. The green module in the WGCNA analysis was most relevant to STAD and was selected for our subsequent analysis, which included 62 genes (Figure 1A–1C). Subsequently, using a significance level of P<0.05, we screened out 38 genes that are significantly associated with prognosis through univariate Cox analysis (Figure 1D). These 38 genes were screened by Random Forest Analysis, Boruta, and XGBoost to identify the final genes (Figures 1E, 1F, 2A–2C). Finally, the intersection of these three machine-learning screened genes was taken, and three genes met our criteria, namely CDH6, EGFLAM, and RASGRF2 (Figure 2D). The Kaplan-Meier survival analysis demonstrated a significant correlation between elevated overall survival and reduced expression of EGFLAM, RASGRF2, and CDH6 (P<0.001, P=0.008, and P<0.001) in contrast to high expression levels (Figure 2E–2G). Furthermore, these three genes exhibited heightened expression levels among non-responsive immunotherapy patients (Supplementary Table 1). These findings imply that gastric cancer patients with elevated expression of these three genes might experience limited or potentially no advantages from immunotherapy.

### Exploration of specific signaling pathways associated with the hub genes EGFLAM, RASGRF2 and CDH6

**Figure 3:**
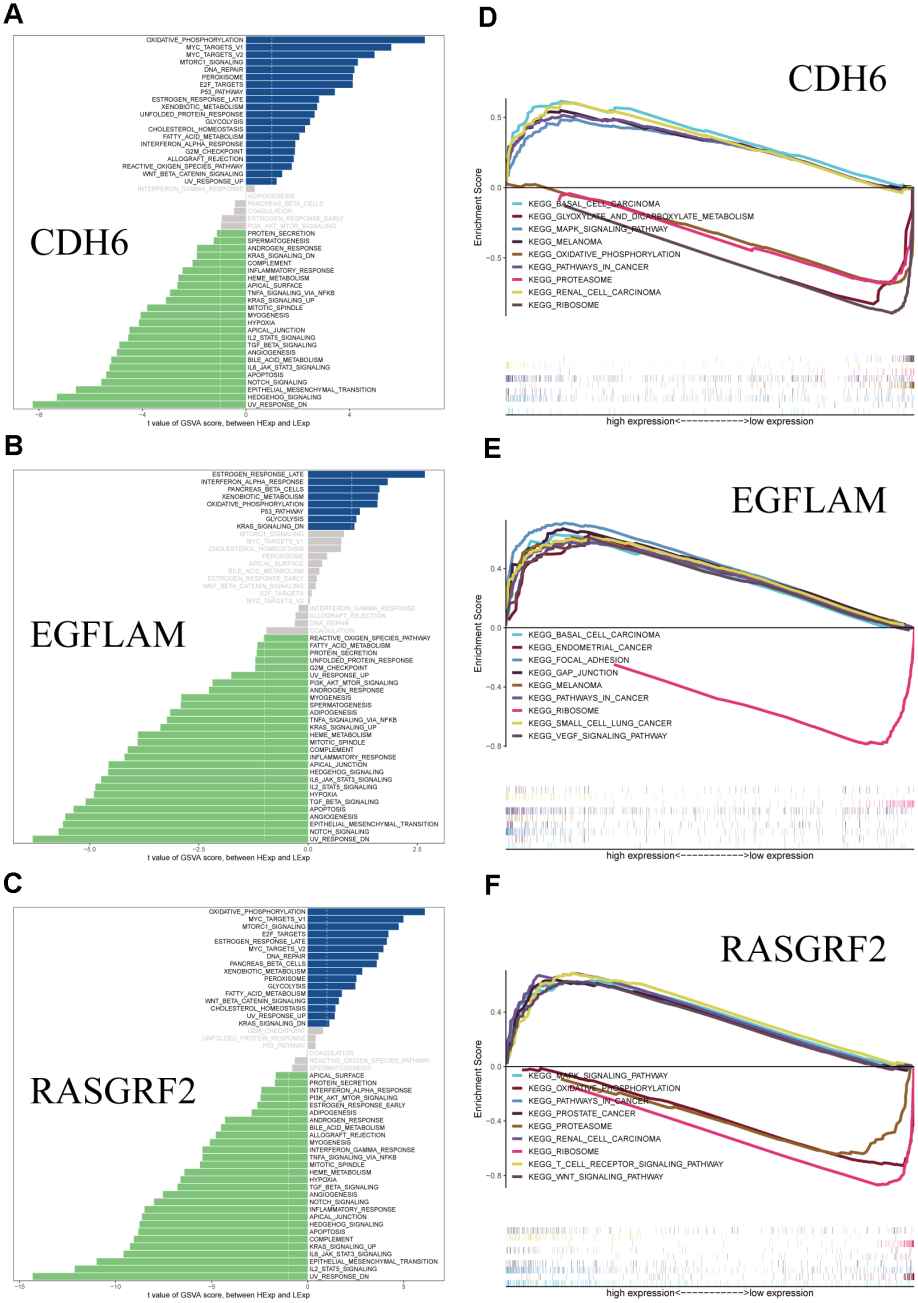
**GSVA and GSEA analysis of the hub genes.** (**A**) GSVA analysis of CDH6. (**B**) GSVA analysis of EGFLAM. (**C**) GSVA analysis of RASGRF2. (**D**) GSEA analysis of CDH6. (**E**) GSEA analysis of EGFLAM. (**F**) GSEA analysis of RASGRF2.

Subsequently, we performed an in-depth analysis of specific signaling pathways involving these three core genes and investigated the potential impact of the candidate genes on pathways related to disease progression. The GSVA analysis revealed that the overexpression of CDH6 is mainly enriched in pathways such as oxidative phosphorylation, P53 signaling pathway, DNA repair, G2M checkpoint, and UV-response-up. Low-expressed CDH6 is mainly enriched in apoptosis, angiogenesis, UV-response-down, TGFβ signaling pathway, IL2-STAT5 signaling pathway, and other pathways. The overexpression of EGFLAM is predominantly enriched in signaling pathways, including the P53 signaling pathway, glycolysis, oxidative phosphorylation, downstream effects of the KRAS signaling pathway, and interferon-alpha response. The low expression of EGFLAM was mainly enriched in the IL6_JAK_STAT3 signaling pathway, apoptosis, PI3K_AKT_MTOR signaling pathway, fatty acid metabolism, TGFβ signaling pathway, and other signaling pathways. The upregulated RASGRF2 is primarily enriched in the MTORC1 signaling pathway, glycolysis, cholesterol homeostasis, MYC target genes, and E2F target genes. Low expression of RASGRF2 was mainly enriched in the PI3K_AKT_MTOR signaling pathway, adipogenesis, apoptosis, TNF-α signaling via NFKB, and protein secretion (Figure 3A–3C). In addition, we performed a GSEA enrichment analysis of these three genes. The results indicate that the elevated expression of CDH6 is enriched in cancer, MAPK signaling pathway, and other pathways. The downregulated expression of CDH6 is enriched in pathways such as glyoxylate and dicarboxylate metabolism, proteasome, and ribosome. The overexpression of EGFLAM is enriched in pathways including focal adhesion, gap junction, cancer, VEGF signaling pathway, and others. Low expression of EGFLAM is enriched in ribosomes. The elevated expression of RASGRF2 is enriched in pathways such as the MAPK signaling pathway, cancer, Wnt signaling pathway, and others. RASGRF2 low expression was enriched in oxidative phosphorylation, proteasome, and ribosome (Figure 3D–3F).

### Analysis of mutations in the three core genes and their relationships with disease-related genes

Searched and selected the top 20 genes most relevant to STAD from the GeneCards database. Comparison between the STAD group and the normal group revealed significant differences in the expression of genes such as CDH1, BRCA2, BRCA1, TP53, APC, and ATM (Supplementary Figure 2A). Through Pearson correlation analysis, we found significant correlations between the three central genes, CDH6, EGFLAM, and RASGRF2, and the expression of several disease-related genes in STAD. As presented in Supplementary Figure 2B, CDH6 was positively correlated with the expression of genes such as APC, ATM, and PIK3CA; the high expression of EGFLAM was correlated with the high expression of genes such as APC, ATM, and PTEN; and RASGRF2 was significantly positively correlated with the expression of genes such as APC, ATM, and MLH1. With the help of the STAD cohort from the cBioportal database, we investigated the mutation status of these three core genes. It was found that all three core genes were mutated to varying degrees, with RASGRF2 at 4%, EGFLAM at 11%, and CDH6 at 8% (Supplementary Figure 3A). Furthermore, we analyzed the mutation rates between the high and low expression groups of these three core genes. Their results showed that the low expression group of the RASGRF2 gene had a higher mutation rate than the high expression group; the mutation rate of the EGFLAM gene was similar between the high and low expression groups; and the low expression group of the CDH6 gene had a higher mutation rate than the high expression group (Supplementary Figure 3B–3D).

### Study of the clinical predictive potential of three core genes based on multi-omics research

The tumor microenvironment is irreplaceable in tumor development, patient survival, and treatment sensitivity. The tumor microenvironment mainly includes tumor cells, immune cells, and mesenchymal stromal cells. By investigating the relationship between the expression of hub genes and tumor immune infiltration, we can gain deeper insights into how these hub genes influence the development of STAD, uncovering potential molecular mechanisms. Supplementary Figure 4A demonstrates the Pearson correlation between immune cells in STAD patients. STAD patients had higher levels of B cells naive, T cells CD4 memory activated, T cells regulatory (Tregs), Macrophages M0, and Macrophages M1 than normal patients (Supplementary Figure 4B). CDH6 was positively correlated with B cells naive, Mast cells resting, T cells CD4 memory resting, etc., and negatively correlated with T cells CD8, T cells CD4 memory activated, and T cells follicular helper; EGFLAM was positively correlated with Macrophages M0, Mast cells activated, Neutrophils, etc., and negatively correlated with NK cells activated, T cells CD8, T cells regulatory (Tregs), etc.; RASGRF2 was positively correlated with B cells naive, Mast cells resting, Macrophages M2 were positively correlated, and negatively correlated with NK cells activated, T cells regulatory (Tregs), Plasma cells, etc. (Supplementary Figure 4C). In addition, we obtained chemokines-related, immunostimulatory-related, immunoinhibitor-related, MHC-related, and receptor-related genes from the TISIDB database and performed a Pearson correlation analysis. The results demonstrate that CDH6, EGFLAM, and RASGRF2 exhibit varying correlations with these immune-related genes (Supplementary Figure 5).

In order to understand the sensitivity of these three pivotal genes to chemotherapeutic drugs, we conducted drug sensitivity studies based on the GDSC database using the “oncoPredict” package. The findings reveal that the expression of CDH6, EGFLAM, and RASGRF2 is correlated with the sensitivity to drugs like Buparlisib, Cediranib, Dasatinib, Dinaciclib, Erlotinib, Niraparib (Supplementary Figure 6).

### Creating nomogram and crafting calibration curves for STAD patient outcome prediction

**Figure 4:**
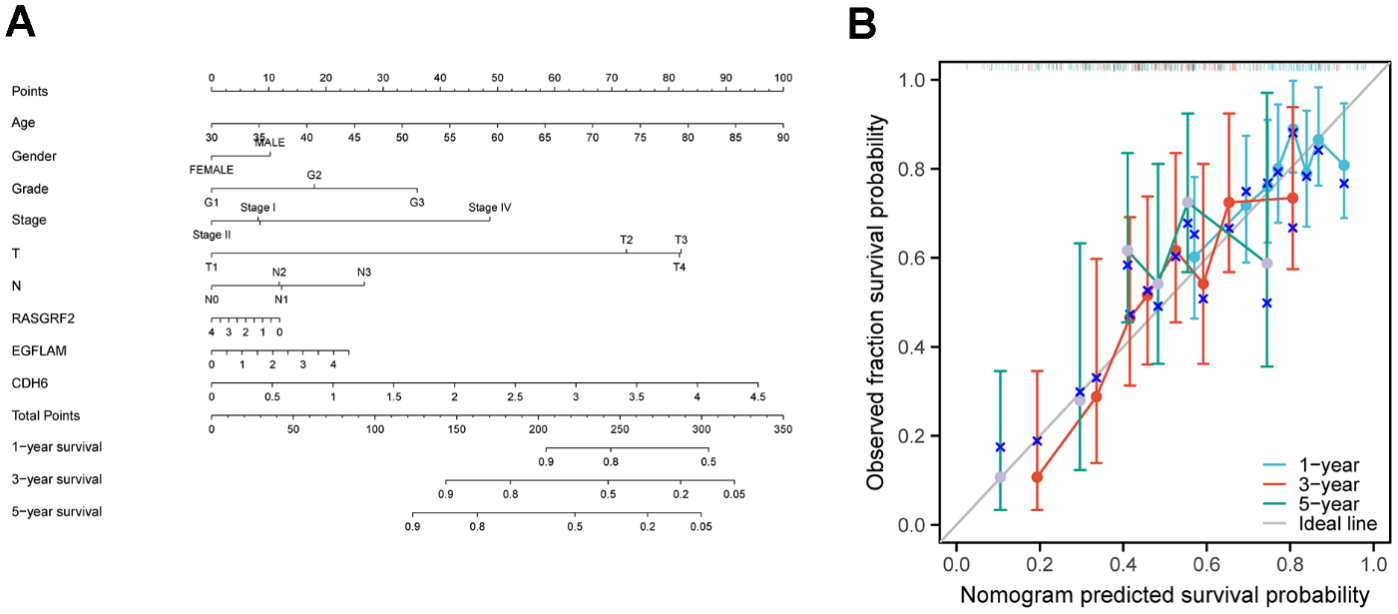
**Construction of a nomogram for prognostic prediction of STAD patients.** (**A**) Nomogram illustrating the 1-year, 3-year, and 5-year overall survival prediction for patients with STAD. (**B**) Calibration curves of a nomogram predicting 1-year, 3-year, and 5-year overall survival.

By incorporating the expression levels of CDH6, EGFLAM, and RASGRF2, along with age, gender, stage, and grade, we effectively developed a prognostic nomogram for anticipating the overall survival of STAD patients. Based on logistic regression analyses, we observed that the three key genes, CDH6, EGFLAM, and RASGRF2, contributed differently at different STAD scoring stages. The higher the overall scores for the three key genes and clinical features, the worse the 1-, 3-, and 5-year overall survival of the patients (Figure 4A). Next, we constructed a calibration curve. The calibration curve showed significant agreement between the predicted and observed OS (Figure 4B).

### Validation of protein expression and cellular expression levels of CDH6, EGFLAM and RASGRF2 genes

We pooled the IHC maps of CDH6, EGFLAM, and RASGRF2 from the HPA database to validate the protein expression levels of these three pivotal genes in clinical specimens. In normal tissues, the expression of CDH6 shows various levels, ranging from low to moderate. However, in gastric cancer patients, the expression of CDH6 also demonstrates diverse degrees, ranging from undetectable low to moderate levels (Supplementary Figure 7A). EGFLAM showed high levels of expression in normal tissues and diverse levels in gastric cancer patients, ranging from undetectable low to moderate to high levels (Supplementary Figure 7C). RASGRF2 showed high levels of expression in normal tissues and diverse levels in gastric cancer patients, ranging from moderate to high (Supplementary Figure 7E). In clinical samples, the variation in protein expression levels of hub genes may account for differences in immune therapy sensitivity among gastric cancer patients. Furthermore, we also analyzed the gene expression levels of hub genes in gastric cancer cell lines using the CCLE database. Noticeable variations were observed in the expression levels of identical hub genes within gastric cancer cell lines. (Supplementary Figure 7B, 7D, 7F).

### Acquisition and functional analysis of CAFs-related genes

**Figure 5:**
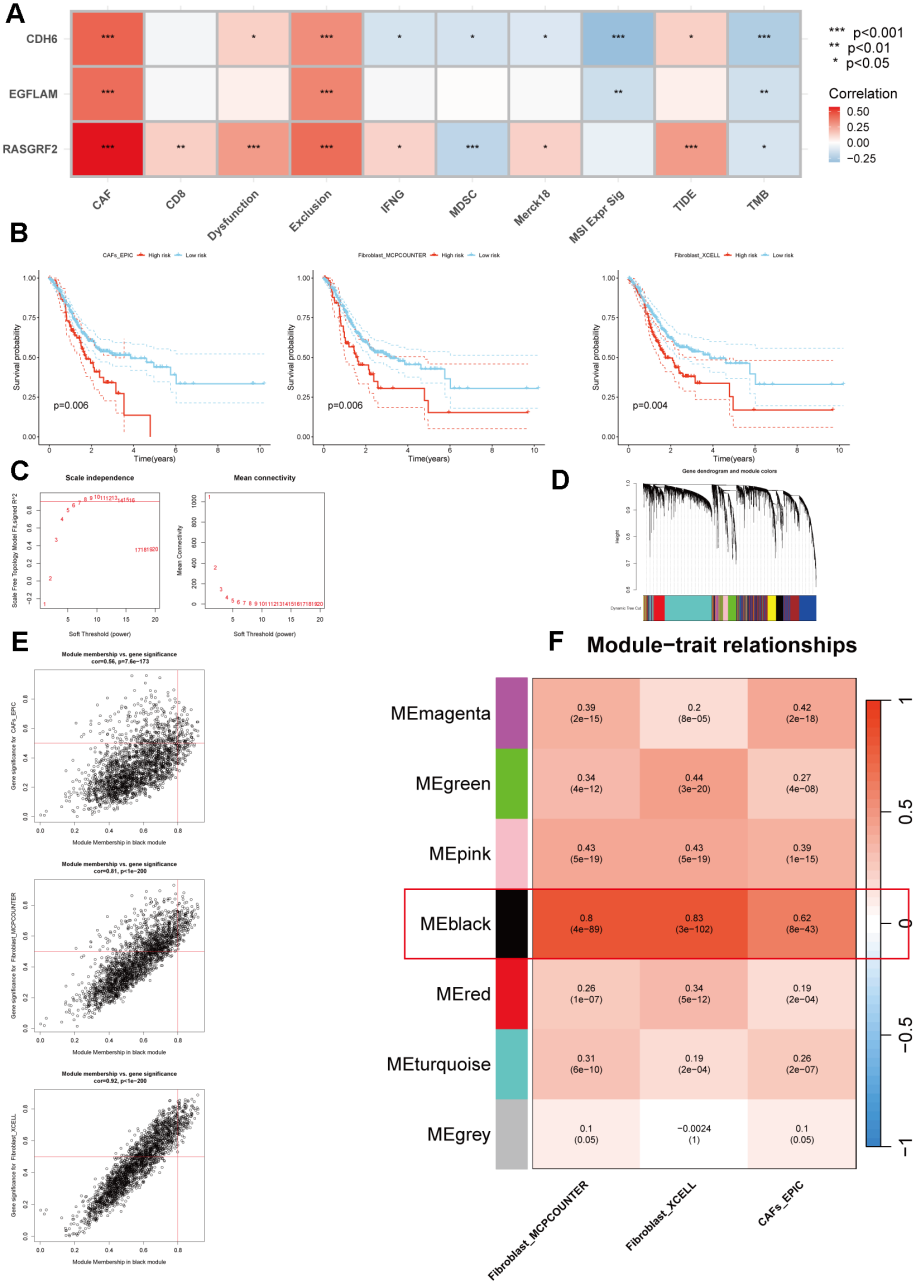
**Multiple methods for identifying CAFs-associated genes related to hub genes.** (**A**) Pearson correlation analysis of hub genes with immunotherapy-related indicators. (**B**) The Kaplan-Meier survival analysis of CAFs scores was calculated using three methods (p=0.006, p=0.006, and p=0.004, respectively). (**C**) Scale independence and mean connectivity analysis. (**D**) Cluster dendrogram among modules. (**E**) Scatterplot of MM and GS from the black module. (**F**) Module-trait relationships. *p < 0.05, **p < 0.01, ***p < 0.001.

A negative correlation between hub genes and TMB was found by calculating TMB and performing Pearson correlation analysis, consistent with poor immunotherapy sensitivity in patients with high hub gene expression. In addition, the TIDE database and Pearson correlation analysis revealed that hub genes were positively correlated with CAFs, Dysfunction, and Exclusion, especially CAFs, with which all three hub genes had significant positive correlations (Figure 5A). Therefore, CAFs may be associated with a low benefit of immunotherapy with high expression of the hub genes. Subsequently, based on the TCGA_STAD cohort, the EPIC, xCell, and MCP-counter algorithms were employed to estimate the content of CAFs for each patient, and all samples were divided into high CAFs and low CAFs groups using the optimal cutoff value of CAFs content. In the TCGA_STAD cohort, those with low CAFs content had better overall survival (P=0.006, P=0.006, and P=0.004) (Figure 5B). In order to obtain the CAFs genes related to hub genes, we performed correlation analysis based on the TCGA_STAD cohort to obtain a total of 4020 genes related to hub genes. For these 4020 genes, we performed WGCNA analysis, in which the black module had the strongest positive correlation with the Fibroblasts_MCPcounter score (Cor = 0.8, P = 4e-89), Fibroblasts_XCELL score (Cor = 0.83, P = 3e-102) and CAFs_EPIC score (Cor = 0.62, P = 8e-43) had the strongest positive correlations (Figure 5C–5F). Finally, we obtained 262 genes in the black module as potential CAFs-associated genes associated with hub genes, using GS > 0.5 as the threshold (Supplementary Table 2).

In order to explore the functions and pathways of these 262 genes and which diseases they are associated with, we performed KEGG, GO, and DO analyses. KEGG enrichment analysis showed that CAFs-related genes were mainly enriched in the PI3K-Akt signaling pathway, cancer, cAMP signaling pathway, ECM-receptor interaction, Wnt signaling pathway, and other pathways (Supplementary Figure 8A). Based on the GO enrichment analysis outcomes, CAFs-associated genes predominantly participated in the regulation of cellular response to growth factor stimulation, extracellular matrix composition, basement membrane, and fibroblast activation (Supplementary Figure 8B). DO results suggest these genes are associated with bone cancer, fibrosarcoma, and connective tissue cancer (Supplementary Figure 8C).

### Model construction driven by CAFs properties

**Figure 6:**
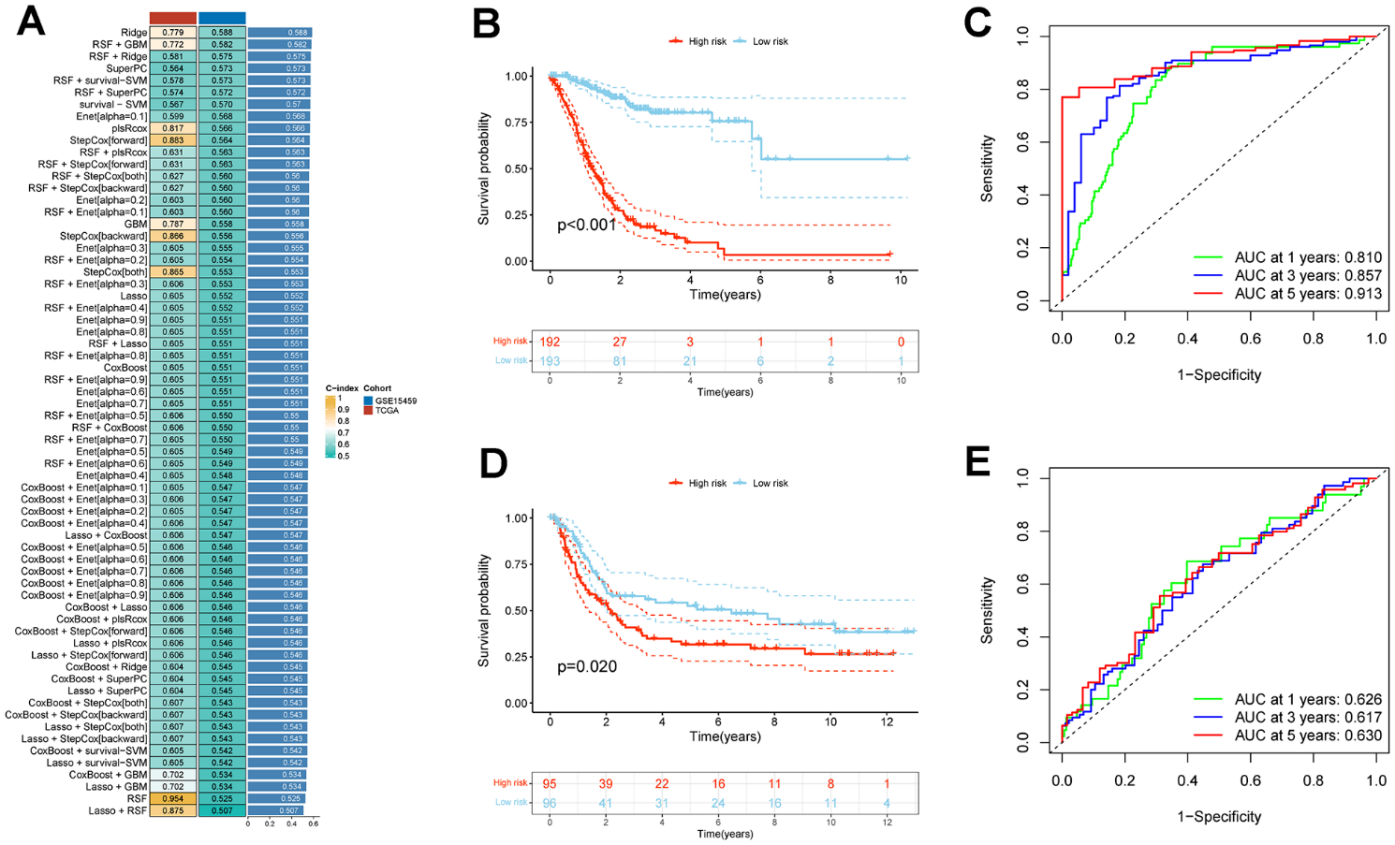
**Establishment and validation of CAFs risk model in STAD through a combination of 10 machine learning approaches.** (**A**) A c-index ranking map of 10 machine learning combinatorial models. (**B**) Kaplan-Meier survival analysis of risk models for CAFs in the TCGA_STAD cohort. (**C**) Time-dependent ROC curves for the TCGA_STAD cohort CAFs risk model. (**D**) Kaplan-Meier survival analysis of risk models for CAFs in the GSE15459 cohort. (**E**) Time-dependent ROC curves for the GSE15459 cohort CAFs risk model.

In the TCGA_STAD cohort, a univariate Cox regression analysis of these 262 genes yielded a total of 218 genes that were associated with prognosis. After taking the intersection with the GSE15459 dataset, 195 genes were obtained for model construction (Supplementary Table 3). Subsequently, these 195 genes were incorporated into combinations of 10 machine algorithms to generate a series of models, and the concordance index (C-index) was calculated for each model. Based on the C-index and model gene count criteria, we ultimately selected the algorithmic model composed of RSF and GBM, which exhibited a higher validation set C-index and fewer genes. This model encompasses 27 genes (Figure 6A).

**Figure 7:**
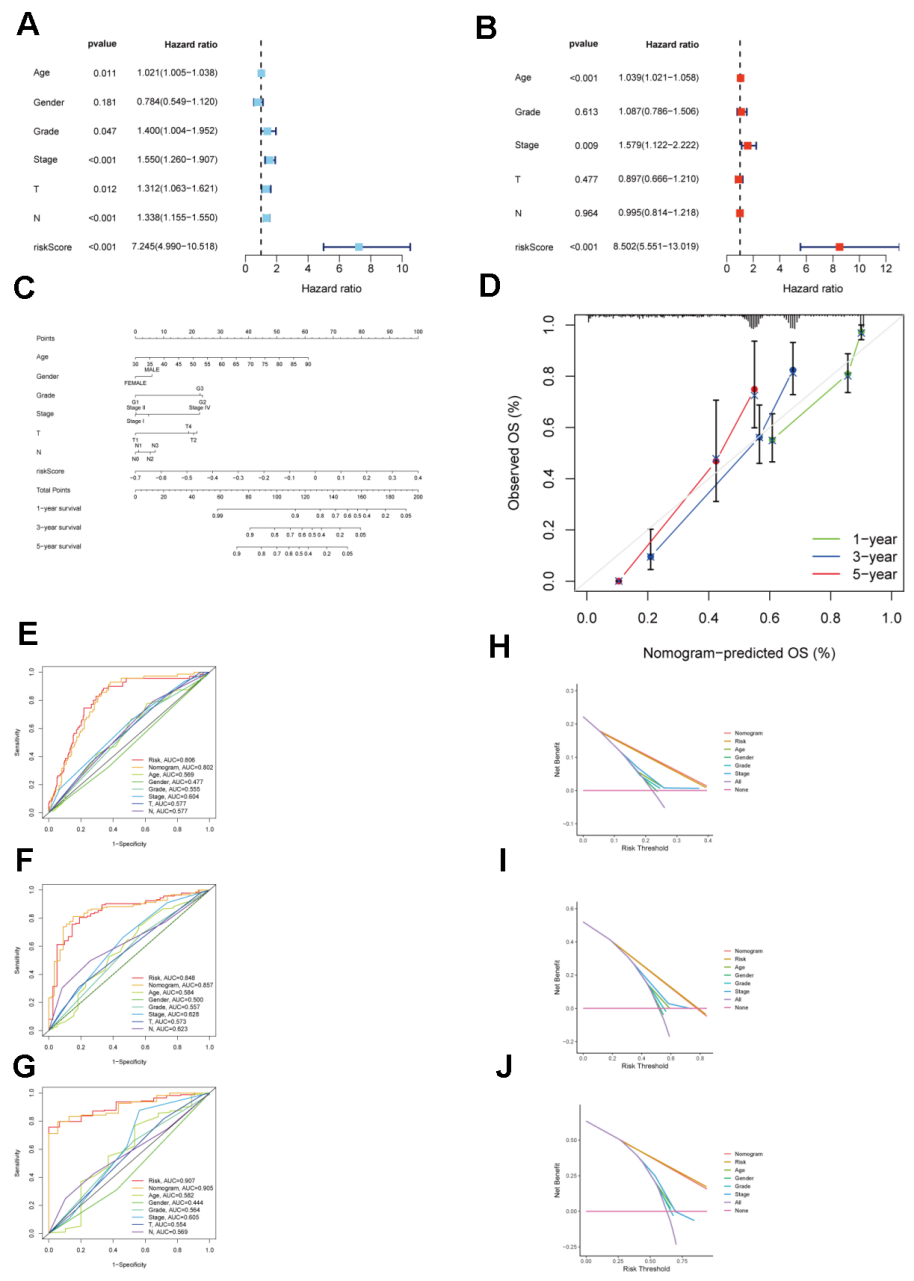
**Validation of the clinical significance of a risk model for CAFs in the TCGA_STAD cohort.** (**A**) Univariate Cox regression analysis for the CAFs risk model in the TCGA STAD cohort. (**B**) Multivariate Cox regression analysis for the CAFs risk model in the TCGA STAD cohort. (**C**) TCGA_STAD on the nomogram of the CAFs risk model. (**D**) Calibration curve of TCGA_STAD on the nomogram of the CAFs risk model. (**E**–**G**) Multivariate ROC curve for 1-year, 3-year, and 5-year. (**H**–**J**) Decision curve for 1-year, 3-year, 5-year.

Patients within each cohort were divided into high and low-risk categories, classified according to the median risk scores derived from the model. In both TCGA_STAD and GSE15459 cohorts, the low-risk group exhibited significantly improved overall survival (P < 0.001 and P = 0.02, respectively) (Figure 6B, 6D). In both the TCGA_STAD and GSE15459 cohorts, the risk score was included in univariate and multivariate Cox regression analyses alongside clinical characteristics. The results indicated that this risk score is an independent prognostic indicator for STAD patients (Figure 7A, 7B). Furthermore, for 1-year, 3-year, and 5-year overall survival rates, ROC curve results indicate that our model possesses excellent predictive performance (Figure 6C, 6E). To develop a tool that could predict the overall survival of STAD patients in the TCGA_STAD cohort, we created a nomogram incorporating clinical features (Figure 7C). Subsequently, we plotted a calibration curve, which showed a high degree of agreement between actual and expected survival (Figure 7D). The results of the multivariate ROC curves for 1-, 3-, and 5-year OS showed clear superiority of our model and nomogram over other clinical features (Figure 7E, 7G, 7I). DCA analyses at 1, 3, and 5 years showed that using a model or nomogram was more favorable than using other clinical characteristics to predict patient prognosis (Figure 7F, 7H, 7J).

### Validation of scRNA-seq for model genes

**Figure 8:**
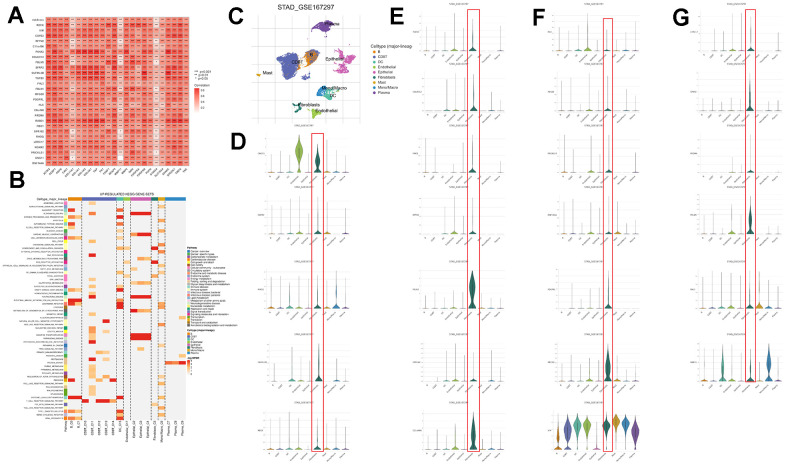
**Validation of genes in the CAFs risk model at the single-cell level.** (**A**) Pearson correlation analysis of risk models with reported CAFs genes. (**B**) GSEA of genes that are upregulated in different cell types. (**C**) Predominant cellular category in single-cell sequencing. (**D**–**G**) Validation of risk model genes for CAFs.

A Pearson correlation analysis was conducted between CAFs risk scores, model gene expression levels, and previously reported CAFs marker genes from existing literature. The examination unveiled a notable positive correlation linking CAFs risk scores, model genes, and the previously documented CAFs marker genes (Figure 8A). To investigate whether model genes are expressed in CAFs, we performed a single-cell analysis based on the TISCH database. We identified scRNA-seq to 9 cell types: B, CD8T, DC, endothelial, epithelial, fibroblasts, Mast, Mono/Macro, and plasma (Figure 8C). Model genes are 25 in this scRNA-seq. The differential analysis showed that most model genes were highly expressed in fibroblasts, especially GNG11, FBLN1, MFGE8, C11orf96, and VIM expression was significantly higher (Figure 8D–8G). Furthermore, the single-cell GSEA-KEGG analysis corroborated the outcomes of the KEGG analysis for CAFs-related genes, illustrating a notable enrichment of fibroblasts within the extracellular matrix-receptor interaction pathway (Figure 8B). These results suggest that the genes in the CAFs-associated models screened and constructed based on machine learning may be CAFs-specific markers.

## Discussion and Conclusions

Gastric cancer is a global tumor with a high clinical burden [26]. It possesses high heterogeneity, and its survival rate is low [27, 28]. In recent years, immunotherapy has developed rapidly and brought breakthroughs in treating gastric cancer. However, the efficacy of immunotherapy in gastric cancer is not apparent. Therefore, an in-depth understanding of the mechanisms of immunotherapy sensitivity is essential to enhance the efficacy of gastric cancer immunotherapy. We comprehensively explored the biomarkers and underlying mechanisms of immunotherapy resistance in gastric cancer patients through bioinformatics analyses.

This study obtained DEGs between immunotherapy responders and non-responders among gastric cancer patients from the ICBatlas database. DEGs include 201 up-regulated and 935 down-regulated genes. The KEGG and GO enrichment analyses revealed significant enrichment of DEGs in multiple tumor-related biological processes, while the PPI results demonstrated intricate interactions among the DEGs. A series of analyses, including WGCNA, univariate Cox, random forest, XGBoost, and Boruta, were performed on the DEGs from the TCGA_STAD cohort, revealing that three genes, namely CDH6, EGFLAM, and RASGRF2, met the criteria. Finally, these three genes were significantly associated with survival by Kaplan-Meier survival analysis and were identified as hub genes for this study. The results of GSVA and GSEA analyses of the three hub genes, CDH6, EGFLAM, and RASGRF2, indicated that their differential expression levels may affect multiple signaling pathways associated with disease progression, including oxidative phosphorylation, DNA repair, apoptosis, angiogenesis, TGFβ signaling, and IL2-STAT5 signaling pathways that may be affected by CDH6. Furthermore, these three hub genes show significant correlations with STAD-related genes, including APC, ATM, MLH1, PIK3CA, PTEN, and others. Gene mutation analyses revealed significant mutation profiles in hub genes, as well as different somatic mutation rates between hub gene expression differences, factors that may potentially influence the efficacy of immunotherapy. Multi-omics studies have revealed significant correlations between CDH6, EGFLAM, and RASGRF2 with tumor immunity, multiple immune-related genes, and chemotherapeutic drug sensitivity. By integrating the expression data of CDH6, EGFLAM, and RASGRF2, as well as clinical features, a nomogram for predicting the prognosis of STAD patients was established. The calibration curves exhibit the remarkable predictive efficacy of the nomogram in prognosticating the outcomes of STAD patients. In addition, we confirmed the expression of these three genes in clinical samples and cell lines with the help of the HPA and CCLE databases. The findings suggest significant variability in the expression levels of these genes in gastric cancer patients and cell lines, which may reflect differences in intrinsic immunotherapeutic susceptibility between patients.

Ultimately, we investigated the connections between the hub gene and additional metrics for assessing immunotherapy, and the study’s findings indicated notable correlations between the hub gene and these other assessment metrics. At the same time, we found a significant relationship between the hub gene and CAFs. CAFs are considered to play a crucial role in the interaction between the tumor microenvironment and cancer cells, driving tumor progression [29]. This implies that hub genes may potentially induce immunotherapy resistance by impacting CAFs. Furthermore, we found that high CAFs scores were associated with poor overall survival in STAD patients, consistent with previous findings. In contrast to traditional DEGs analysis for selecting CAFs markers [30], we employed various bioinformatics algorithms to assess the abundance of CAFs in each STAD sample, ensuring the robustness of the constructed WGCNA network. The KEGG, GO, and DO analyses performed on CAFs-related genes revealed their close association with the occurrence and progression of tumors. Similarly, to ensure the reliability of predictive features, we employed a combination of 10 different machine-learning algorithms for construction and validation. Additionally, we compared the model and its constituent genes with previously identified CAFs markers, revealing their close association. Also, at the single-cell level, we verified that the genes included in the model have high expression in CAFs. The above findings imply a strong association between our selected genes and CAFs, and the model we constructed can accurately predict patient prognosis.

CDH6, or CAD6 or KCAD, is a cadherin (CDH) family member. Recent research has indicated that CDH molecules play a significant role in tumor initiation, growth, and progression, potentially serving as diagnostic markers, prognostic indicators, and potential therapeutic targets for cancer patients [31, 32]. As one of the family members, the CDH6 protein has five extracellular structural domains and one cytoplasmic structural domain, a particular structure that makes it unique from other family members in terms of its interaction with connexin molecules [33]. Elevated CDH6 expression is closely linked to unfavorable prognostic outcomes across multiple malignant tumors. In papillary thyroid carcinoma, aberrant up-regulation of CDH6 may promote epithelial-mesenchymal transformation and cancer cell metastasis by regulating autophagic processes [34, 35]. CDH6 is highly expressed in renal cancer and is associated with lymph node invasion and metastasis [36]. Furthermore, CDH6 shows a high expression level in osteosarcoma, which is closely associated with patient prognosis [37]. In addition, heightened CDH6 expression in gastric cancer is connected to tumor advancement and unfavorable prognostic implications [38]. Finally, CDH6 is also overexpressed in tumors such as snuff carcinoma, ovarian carcinoma, and oral squamous carcinoma and may be involved in the prognosis of patients [39–41]. To the best of our knowledge, studies on CDH6 have been limited to its effects on tumors, and no studies have examined the role of CDH6 in immunotherapy resistance in gastric cancer.

RASGRF2, functioning as a guanylate exchange factor for RasGTPase, participates in diverse cellular processes and contributes to tumor progression, migration, and invasion. Recent genome-wide association studies have unveiled a link between RASGRF2 and the susceptibility to malignant mesothelioma (MM) [42]. High expression of RASGRF2 inhibits tumor migration invasion in colorectal cancer [43]. However, high RASGRF2 expression in lung adenocarcinoma is associated with tumor invasion and poor prognosis [44]. However, in-depth studies and research on the function of RASGRF2 in gastric cancer and its importance in immunotherapy resistance are still lacking. The role of EGFLAM, EGF-like, type III fibronectin, and laminin G structural domains in tumors must be studied more. In recent studies, it has been found that EGFLAM exhibits significant hypomethylation in ovarian cancer [45], while in glioblastoma, EGFLAM is associated with tumor cell migration, invasion, and adverse prognosis [46].

However, our study has some limitations and shortcomings. First, given the high heterogeneity of gastric cancer, while the number of samples we retrieved from the TCGA and GEO databases was limited, this would lead to possible bias in the results. Second, these results have not been confirmed by *in vivo* and *in vitro* experiments. Despite these shortcomings, the preliminary study provides valuable and constructive basic information. In the subsequent study, we will delve into the role of CDH6, EGFLAM, and RASGRF2 as hub genes in gastric cancer immunotherapy resistance through a series of experiments. We will also investigate the effects of CDH6, EGFLAM, and RASGRF2 on gastric cancer proliferation, migration, and invasion in both *in vivo* and *in vitro* contexts.

In summary, we identified genes associated with immunotherapy resistance in gastric cancer based on a machine learning approach, which provides a new biological marker for determining the sensitivity of gastric cancer patients to immunotherapy. In addition, CAFs scores constructed using CAFs-related genes based on these resistance genes can effectively predict the prognosis of patients and facilitate clinical decision-making.

## Supplementary Material

Supplementary Figures

Supplementary Table 1

Supplementary Table 2

Supplementary Table 3

## References

[1] Fitzmaurice C, Akinyemiju TF, Al Lami FH, Alam T, Alizadeh-Navaei R, Allen C, Alsharif U, Alvis-Guzman N, Amini E, Anderson BO, Aremu O, Artaman A, Asgedom SW, et al, and Global Burden of Disease Cancer Collaboration. Global, Regional, and National Cancer Incidence, Mortality, Years of Life Lost, Years Lived With Disability, and Disability-Adjusted Life-Years for 29 Cancer Groups, 1990 to 2016: A Systematic Analysis for the Global Burden of Disease Study. JAMA Oncol. 2018; 4:1553–68. 10.1001/jamaoncol.2018.2706 29860482 PMC6248091

[2] Sung H, Ferlay J, Siegel RL, Laversanne M, Soerjomataram I, Jemal A, Bray F. Global Cancer Statistics 2020: GLOBOCAN Estimates of Incidence and Mortality Worldwide for 36 Cancers in 185 Countries. CA Cancer J Clin. 2021; 71:209–49. 10.3322/caac.21660 33538338

[3] Xie C, Lu NH. Review: clinical management of Helicobacter pylori infection in China. Helicobacter. 2015; 20:1–10. 10.1111/hel.12178 25382801

[4] Xia C, Dong X, Li H, Cao M, Sun D, He S, Yang F, Yan X, Zhang S, Li N, Chen W. Cancer statistics in China and United States, 2022: profiles, trends, and determinants. Chin Med J (Engl). 2022; 135:584–90. 10.1097/CM9.0000000000002108 35143424 PMC8920425

[5] Digklia A, Wagner AD. Advanced gastric cancer: Current treatment landscape and future perspectives. World J Gastroenterol. 2016; 22:2403–14. 10.3748/wjg.v22.i8.2403 26937129 PMC4768187

[6] Goel G, Sun W. Cancer immunotherapy in clinical practice -- the past, present, and future. Chin J Cancer. 2014; 33:445–57. 10.5732/cjc.014.10123 25189717 PMC4190434

[7] Bonotto M, Garattini SK, Basile D, Ongaro E, Fanotto V, Cattaneo M, Cortiula F, Iacono D, Cardellino GG, Pella N, Fasola G, Antonuzzo L, Silvestris N, Aprile G. Immunotherapy for gastric cancers: emerging role and future perspectives. Expert Rev Clin Pharmacol. 2017; 10:609–19. 10.1080/17512433.2017.1313113 28349740

[8] Hodi FS, O’Day SJ, McDermott DF, Weber RW, Sosman JA, Haanen JB, Gonzalez R, Robert C, Schadendorf D, Hassel JC, Akerley W, van den Eertwegh AJ, Lutzky J, et al. Improved survival with ipilimumab in patients with metastatic melanoma. N Engl J Med. 2010; 363:711–23. 10.1056/NEJMoa1003466 20525992 PMC3549297

[9] Brahmer JR, Tykodi SS, Chow LQ, Hwu WJ, Topalian SL, Hwu P, Drake CG, Camacho LH, Kauh J, Odunsi K, Pitot HC, Hamid O, Bhatia S, et al. Safety and activity of anti-PD-L1 antibody in patients with advanced cancer. N Engl J Med. 2012; 366:2455–65. 10.1056/NEJMoa1200694 22658128 PMC3563263

[10] Patel SP, Kurzrock R. PD-L1 Expression as a Predictive Biomarker in Cancer Immunotherapy. Mol Cancer Ther. 2015; 14:847–56. 10.1158/1535-7163.MCT-14-0983 25695955

[11] Hudler P. Challenges of deciphering gastric cancer heterogeneity. World J Gastroenterol. 2015; 21:10510–27. 10.3748/wjg.v21.i37.10510 26457012 PMC4588074

[12] Marrelli D, Polom K, Pascale V, Vindigni C, Piagnerelli R, De Franco L, Ferrara F, Roviello G, Garosi L, Petrioli R, Roviello F. Strong Prognostic Value of Microsatellite Instability in Intestinal Type Non-cardia Gastric Cancer. Ann Surg Oncol. 2016; 23:943–50. 10.1245/s10434-015-4931-3 26530444

[13] Högner A, Moehler M. Immunotherapy in Gastric Cancer. Curr Oncol. 2022; 29:1559–74. 10.3390/curroncol29030131 35323331 PMC8946975

[14] Szklarczyk D, Morris JH, Cook H, Kuhn M, Wyder S, Simonovic M, Santos A, Doncheva NT, Roth A, Bork P, Jensen LJ, von Mering C. The STRING database in 2017: quality-controlled protein-protein association networks, made broadly accessible. Nucleic Acids Res. 2017; 45:D362–8. 10.1093/nar/gkw937 27924014 PMC5210637

[15] Langfelder P, Horvath S. WGCNA: an R package for weighted correlation network analysis. BMC Bioinformatics. 2008; 9:559. 10.1186/1471-2105-9-559 19114008 PMC2631488

[16] Li W, Yin Y, Quan X, Zhang H. Gene Expression Value Prediction Based on XGBoost Algorithm. Front Genet. 2019; 10:1077. 10.3389/fgene.2019.01077 31781160 PMC6861218

[17] Hänzelmann S, Castelo R, Guinney J. GSVA: gene set variation analysis for microarray and RNA-seq data. BMC Bioinformatics. 2013; 14:7. 10.1186/1471-2105-14-7 23323831 PMC3618321

[18] Powers RK, Goodspeed A, Pielke-Lombardo H, Tan AC, Costello JC. GSEA-InContext: identifying novel and common patterns in expression experiments. Bioinformatics. 2018; 34:i555–64. 10.1093/bioinformatics/bty271 29950010 PMC6022535

[19] Mayakonda A, Lin DC, Assenov Y, Plass C, Koeffler HP. Maftools: efficient and comprehensive analysis of somatic variants in cancer. Genome Res. 2018; 28:1747–56. 10.1101/gr.239244.118 30341162 PMC6211645

[20] Chen B, Khodadoust MS, Liu CL, Newman AM, Alizadeh AA. Profiling Tumor Infiltrating Immune Cells with CIBERSORT. Methods Mol Biol. 2018; 1711:243–59. 10.1007/978-1-4939-7493-1_12 29344893 PMC5895181

[21] Racle J, de Jonge K, Baumgaertner P, Speiser DE, Gfeller D. Simultaneous enumeration of cancer and immune cell types from bulk tumor gene expression data. Elife. 2017; 6:e26476. 10.7554/eLife.26476 29130882 PMC5718706

[22] Aran D, Hu Z, Butte AJ. xCell: digitally portraying the tissue cellular heterogeneity landscape. Genome Biol. 2017; 18:220. 10.1186/s13059-017-1349-1 29141660 PMC5688663

[23] Becht E, Giraldo NA, Lacroix L, Buttard B, Elarouci N, Petitprez F, Selves J, Laurent-Puig P, Sautès-Fridman C, Fridman WH, de Reyniès A. Estimating the population abundance of tissue-infiltrating immune and stromal cell populations using gene expression. Genome Biol. 2016; 17:218. 10.1186/s13059-016-1070-5 27765066 PMC5073889

[24] Yu G, Wang LG, Yan GR, He QY. DOSE: an R/Bioconductor package for disease ontology semantic and enrichment analysis. Bioinformatics. 2015; 31:608–9. 10.1093/bioinformatics/btu684 25677125

[25] Sun D, Wang J, Han Y, Dong X, Ge J, Zheng R, Shi X, Wang B, Li Z, Ren P, Sun L, Yan Y, Zhang P, et al. TISCH: a comprehensive web resource enabling interactive single-cell transcriptome visualization of tumor microenvironment. Nucleic Acids Res. 2021; 49:D1420–30. 10.1093/nar/gkaa1020 33179754 PMC7778907

[26] Zeng Y, Jin RU. Molecular pathogenesis, targeted therapies, and future perspectives for gastric cancer. Semin Cancer Biol. 2022; 86:566–82. 10.1016/j.semcancer.2021.12.004 34933124 PMC12833737

[27] López MJ, Carbajal J, Alfaro AL, Saravia LG, Zanabria D, Araujo JM, Quispe L, Zevallos A, Buleje JL, Cho CE, Sarmiento M, Pinto JA, Fajardo W. Characteristics of gastric cancer around the world. Crit Rev Oncol Hematol. 2023; 181:103841. 10.1016/j.critrevonc.2022.103841 36240980

[28] Amin MB, Greene FL, Edge SB, Compton CC, Gershenwald JE, Brookland RK, Meyer L, Gress DM, Byrd DR, Winchester DP. The Eighth Edition AJCC Cancer Staging Manual: Continuing to build a bridge from a population-based to a more “personalized” approach to cancer staging. CA Cancer J Clin. 2017; 67:93–9. 10.3322/caac.21388 28094848

[29] De P, Aske J, Dey N. Cancer-Associated Fibroblast Functions as a Road-Block in Cancer Therapy. Cancers (Basel). 2021; 13:5246. 10.3390/cancers13205246 34680395 PMC8534063

[30] Kim MJ, Jung D, Park JY, Lee SM, An HJ. GLIS1 in Cancer-Associated Fibroblasts Regulates the Migration and Invasion of Ovarian Cancer Cells. Int J Mol Sci. 2022; 23:2218. 10.3390/ijms23042218 35216340 PMC8874490

[31] Loh CY, Chai JY, Tang TF, Wong WF, Sethi G, Shanmugam MK, Chong PP, Looi CY. The E-Cadherin and N-Cadherin Switch in Epithelial-to-Mesenchymal Transition: Signaling, Therapeutic Implications, and Challenges. Cells. 2019; 8:1118. 10.3390/cells8101118 31547193 PMC6830116

[32] Hansford S, Kaurah P, Li-Chang H, Woo M, Senz J, Pinheiro H, Schrader KA, Schaeffer DF, Shumansky K, Zogopoulos G, Santos TA, Claro I, Carvalho J, et al. Hereditary Diffuse Gastric Cancer Syndrome: CDH1 Mutations and Beyond. JAMA Oncol. 2015; 1:23–32. 10.1001/jamaoncol.2014.168 26182300

[33] Casal JI, Bartolomé RA. Beyond N-Cadherin, Relevance of Cadherins 5, 6 and 17 in Cancer Progression and Metastasis. Int J Mol Sci. 2019; 20:3373. 10.3390/ijms20133373 31324051 PMC6651558

[34] Gugnoni M, Sancisi V, Gandolfi G, Manzotti G, Ragazzi M, Giordano D, Tamagnini I, Tigano M, Frasoldati A, Piana S, Ciarrocchi A. Cadherin-6 promotes EMT and cancer metastasis by restraining autophagy. Oncogene. 2017; 36:667–77. 10.1038/onc.2016.237 27375021

[35] Ciarrocchi A, Piana S, Valcavi R, Gardini G, Casali B. Inhibitor of DNA binding-1 induces mesenchymal features and promotes invasiveness in thyroid tumour cells. Eur J Cancer. 2011; 47:934–45. 10.1016/j.ejca.2010.11.009 21146400

[36] Paul R, Ewing CM, Robinson JC, Marshall FF, Johnson KR, Wheelock MJ, Isaacs WB. Cadherin-6, a cell adhesion molecule specifically expressed in the proximal renal tubule and renal cell carcinoma. Cancer Res. 1997; 57:2741–8. 9205085

[37] Ji Q, Xu X, Song Q, Xu Y, Tai Y, Goodman SB, Bi W, Xu M, Jiao S, Maloney WJ, Wang Y. miR-223-3p Inhibits Human Osteosarcoma Metastasis and Progression by Directly Targeting CDH6. Mol Ther. 2018; 26:1299–312. 10.1016/j.ymthe.2018.03.009 29628305 PMC5993963

[38] Zhao Z, Li S, Li S, Wang J, Lin H, Fu W. High expression of oncogene cadherin-6 correlates with tumor progression and a poor prognosis in gastric cancer. Cancer Cell Int. 2021; 21:493. 10.1186/s12935-021-02071-y 34530820 PMC8447617

[39] Zuo LL, Zhang J, Liu LZ, Zhou Q, Du SJ, Xin SY, Ning ZP, Yang J, Yu HB, Yue WX, Wang J, Zhu FX, Li GY, Lu JH. Cadherin 6 is activated by Epstein-Barr virus LMP1 to mediate EMT and metastasis as an interplay node of multiple pathways in nasopharyngeal carcinoma. Oncogenesis. 2017; 6:402. 10.1038/s41389-017-0005-7 29284791 PMC5865538

[40] Karthikeyan S, Lantvit DD, Chae DH, Burdette JE. Cadherin-6 type 2, K-cadherin (CDH6) is regulated by mutant p53 in the fallopian tube but is not expressed in the ovarian surface. Oncotarget. 2016; 7:69871–82. 10.18632/oncotarget.11499 27563818 PMC5342521

[41] Ma C, Zhao JZ, Lin RT, Zhou L, Chen YN, Yu LJ, Shi TY, Wang M, Liu MM, Liu YR, Zhang T. Combined overexpression of cadherin 6, cadherin 11 and cluster of differentiation 44 is associated with lymph node metastasis and poor prognosis in oral squamous cell carcinoma. Oncol Lett. 2018; 15:9498–506. 10.3892/ol.2018.8509 29805672 PMC5958757

[42] Cadby G, Mukherjee S, Musk AW, Reid A, Garlepp M, Dick I, Robinson C, Hui J, Fiorito G, Guarrera S, Beilby J, Melton PE, Moses EK, et al. A genome-wide association study for malignant mesothelioma risk. Lung Cancer. 2013; 82:1–8. 10.1016/j.lungcan.2013.04.018 23827383

[43] Lu P, Chen J, Yan L, Yang L, Zhang L, Dai J, Hao Z, Bai T, Xi Y, Li Y, Kang Z, Xv J, Sun G, Yang T. RasGRF2 promotes migration and invasion of colorectal cancer cells by modulating expression of MMP9 through Src/Akt/NF-κB pathway. Cancer Biol Ther. 2019; 20:435–43. 10.1080/15384047.2018.1529117 30359168 PMC6422503

[44] Nakagawa T, Kim Y, Kano J, Murata Y, Kosibaty Z, Noguchi M, Sakamoto N. High expression of Ras-specific guanine nucleotide-releasing factor 2 (RasGRF2) in lung adenocarcinoma is associated with tumor invasion and poor prognosis. Pathol Int. 2021; 71:255–60. 10.1111/pin.13069 33709437 PMC8251786

[45] Gu XH, Lu Y, Ma D, Liu XS, Guo SW. [Model of aberrant DNA methylation patterns and its applications in epithelial ovarian cancer]. Zhonghua Fu Chan Ke Za Zhi. 2009; 44:754–9. 20078962

[46] Chen J, Zhang J, Hong L, Zhou Y. EGFLAM correlates with cell proliferation, migration, invasion and poor prognosis in glioblastoma. Cancer Biomark. 2019; 24:343–50. 10.3233/CBM-181740 30829611 PMC6484271

