# Supplementary material for: Integrated multi-omics analysis and machine learning identify hub genes and potential mechanisms of resistance to immunotherapy in gastric cancer
Source: Aging (Albany NY). 2024 Apr 22;16(8):7331–56. doi: 10.18632/aging.205760 (PMC11087130; doi:10.18632/aging.205760)
Supplement: Supplementary Figures [file aging-16-205760-s001.pdf]

SUPPLEMENTARY FIGURES

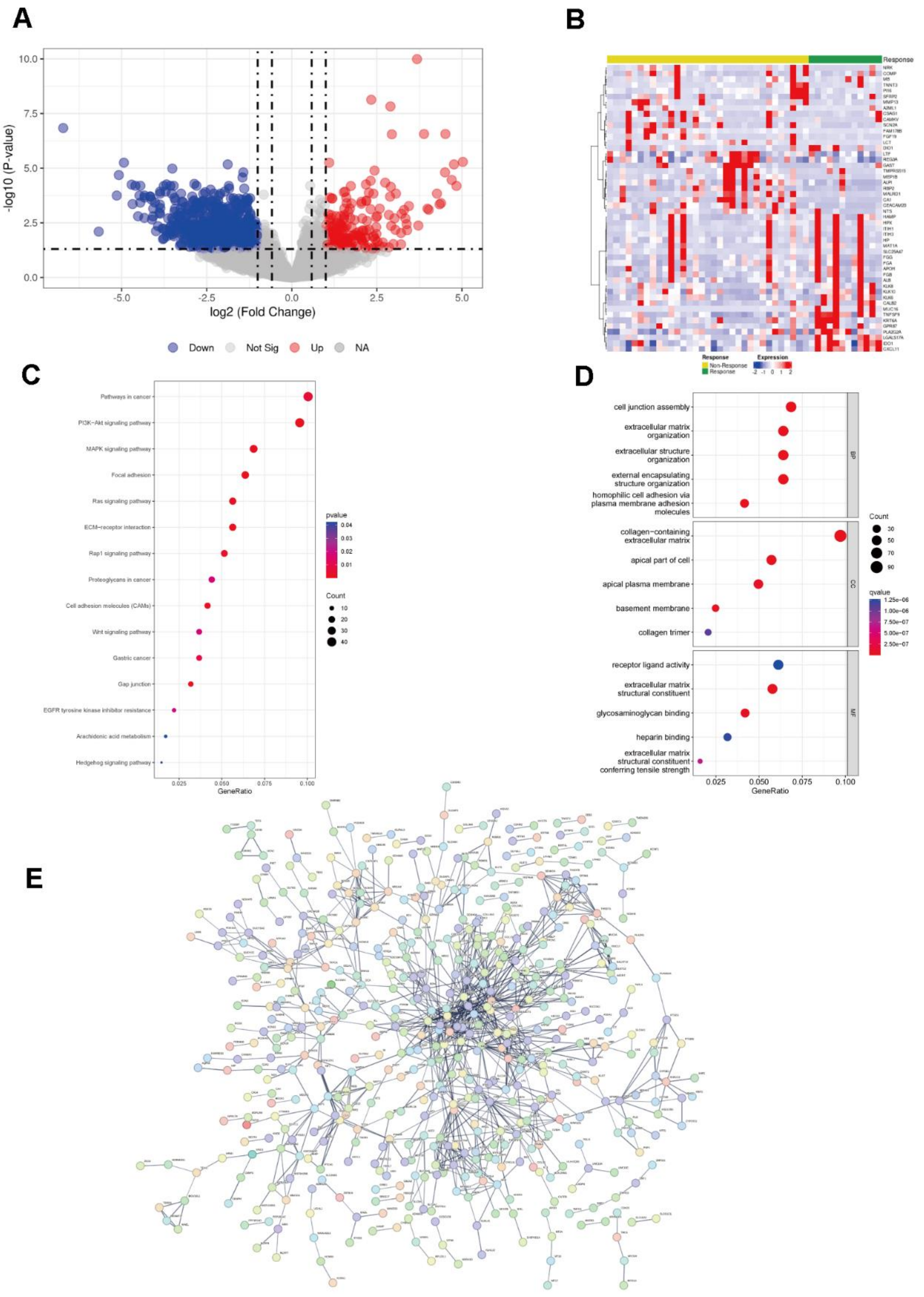

Supplementary Figure 1. Identification of differentially expressed genes (DEGs) associated with immunotherapy. (**A**) Volcano plot of DEGs. (**B**) Heatmap of DEGs. (**C**) KEGG analysis of DEGs. (**D**) GO analysis of DEGs. (**E**) PPI of DEGs.

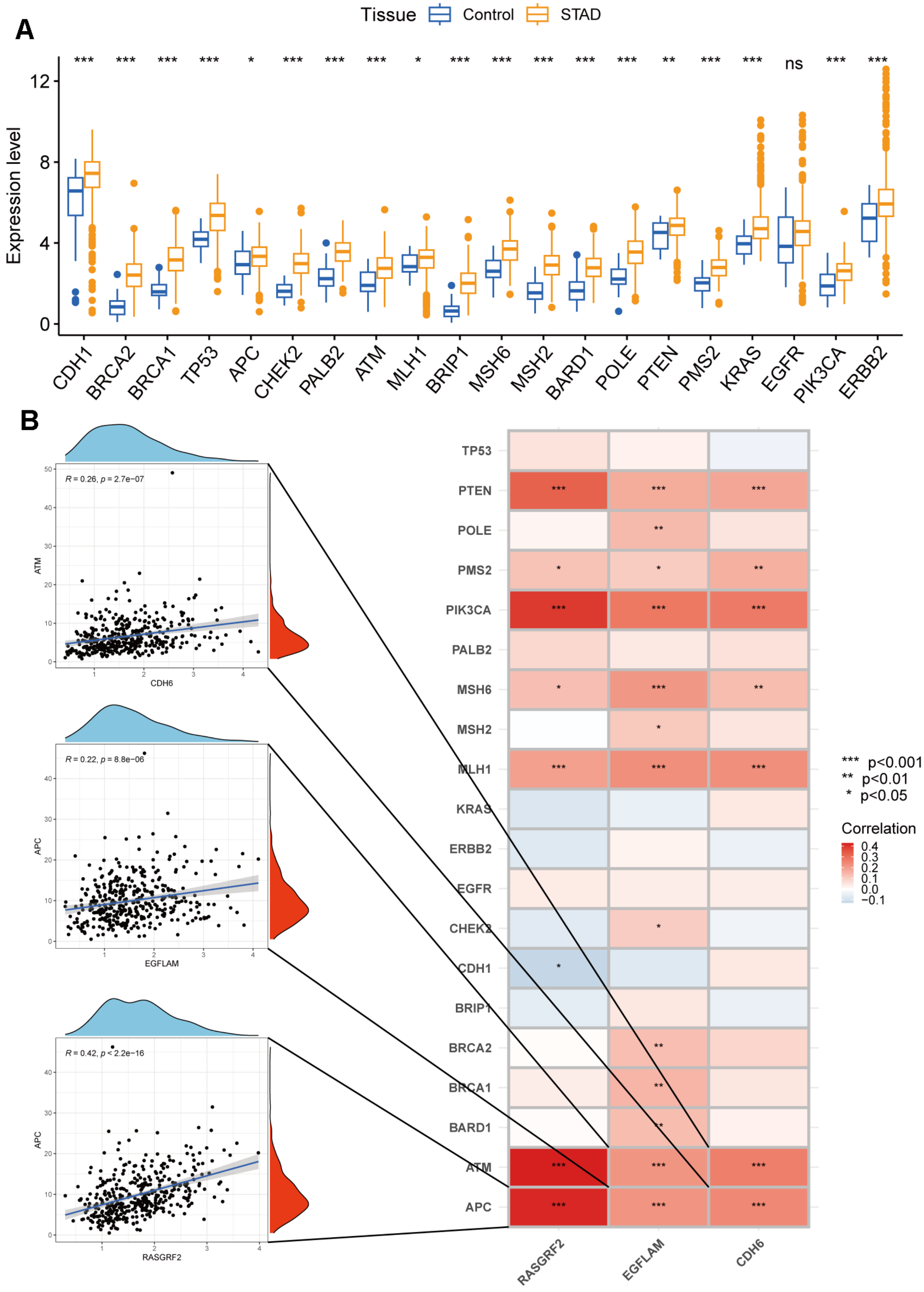

Supplementary Figure 2. Exploration of interrelationships between hub genes and disease-related genes. (**A**) Comparison of disease-related genes in normal and tumor tissues. (**B**) Pearson correlation analysis of hub genes and disease-associated genes. *p < 0.05, **p < 0.01, ***p < 0.001.

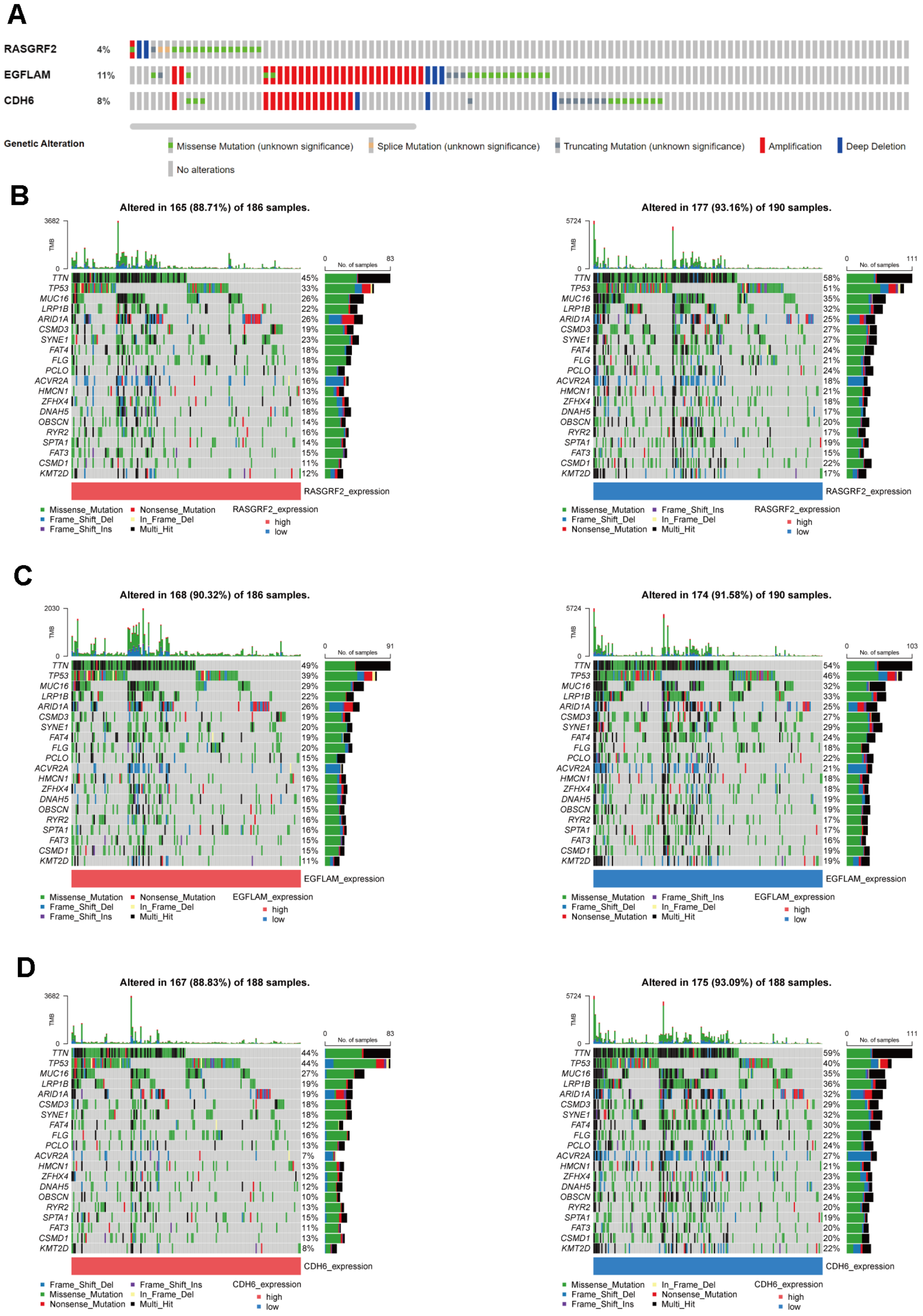

Supplementary Figure 3. Mutation analysis of the hub genes. (**A**) Analysis of genetic variation in the hub genes of the TCGA_STAD cohort. (**B**–**D**) Mutational analysis of somatic cells with different RASGRF2, EGFLAM, and CDH6 expressions.

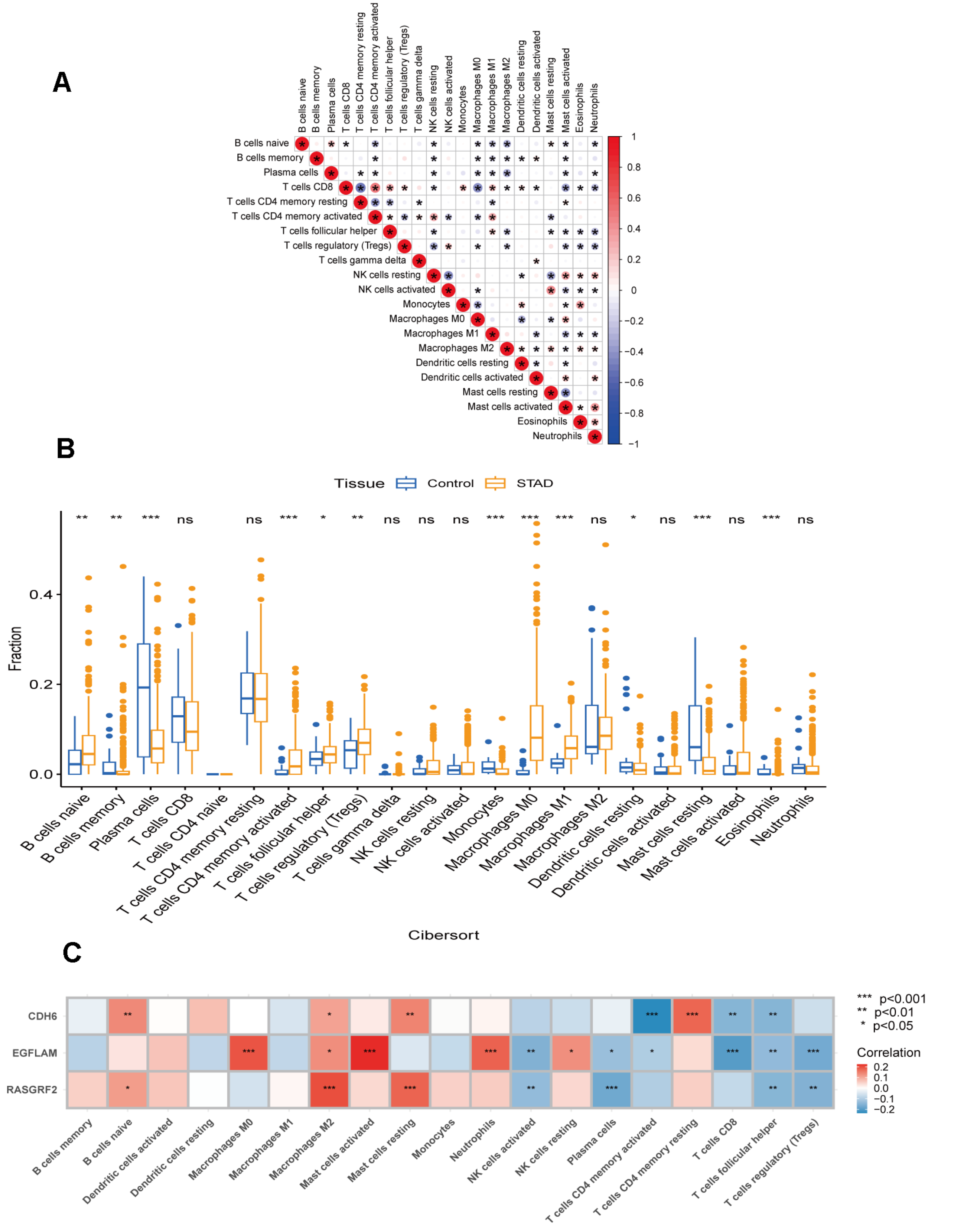

Supplementary Figure 4. Analysis of Pearson correlation between hub genes and immune cells. (**A**) Correlation analysis of 22 immune cells in STAD patients. (**B**) Comparison of 22 immune cells in normal and tumor tissues. (**C**) Analysis of hub genes correlation with 22 types of immune cells. *p < 0.05, **p < 0.01, ***p < 0.001.

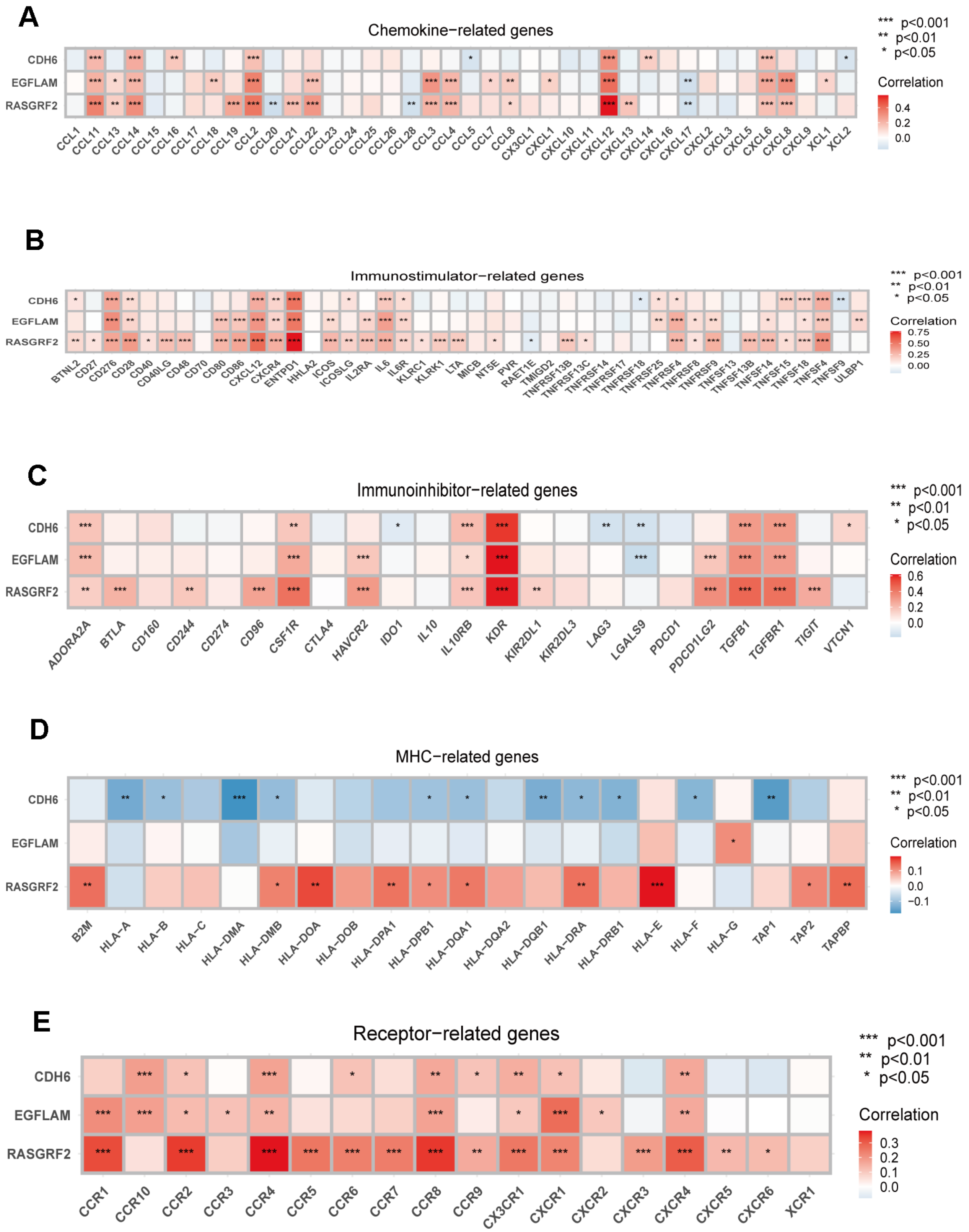

Supplementary Figure 5. Pearson correlation analysis of hub genes with immune-related genes. (**A**) Chemokine-related genes. (**B**) Immunostimulator-related genes. (**C**) Immunoinhibitor-related genes. (**D**) MHC-related genes. (**E**) Receptor-related genes. *p < 0.05, **p < 0.01, ***p < 0.001.

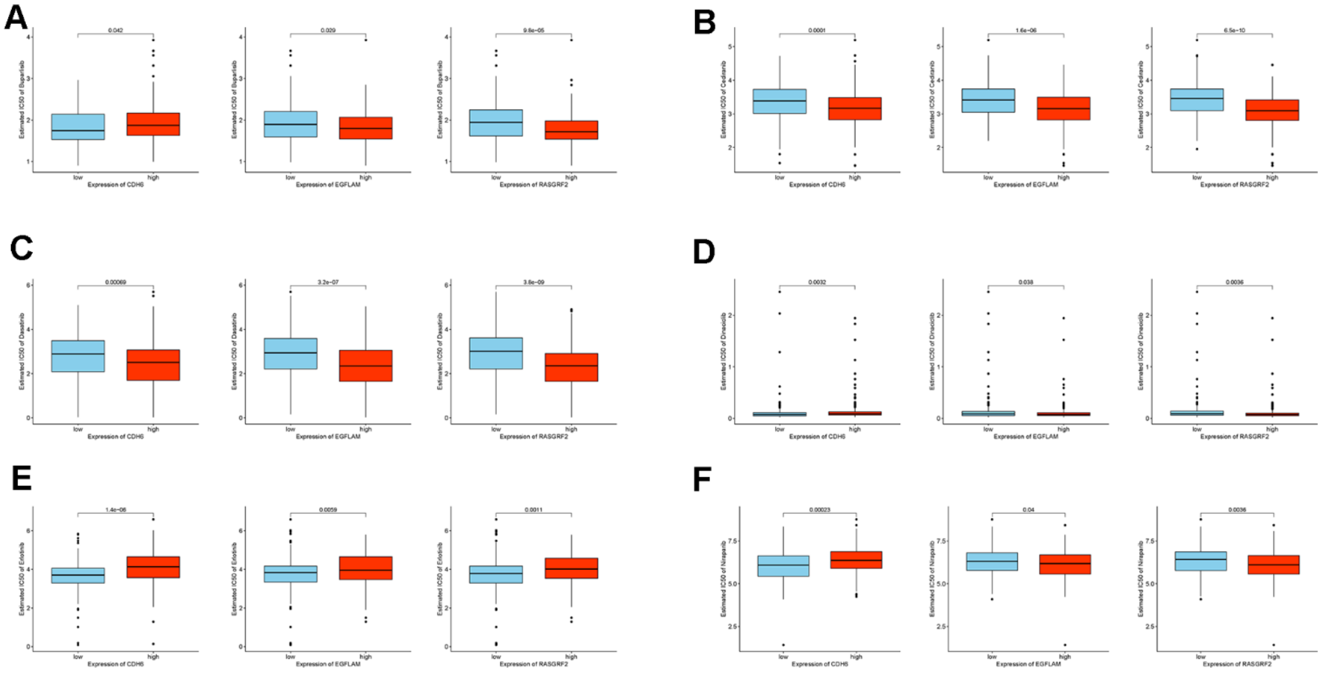

Supplementary Figure 6. Drug sensitivity analysis of the hub genes. (**A**) Buparlisib. (**B**) Cediranib. (**C**) Dasatinib. (**D**) Dinaciclib. (**E**) Erlotinib. (**F**) Niraparib.

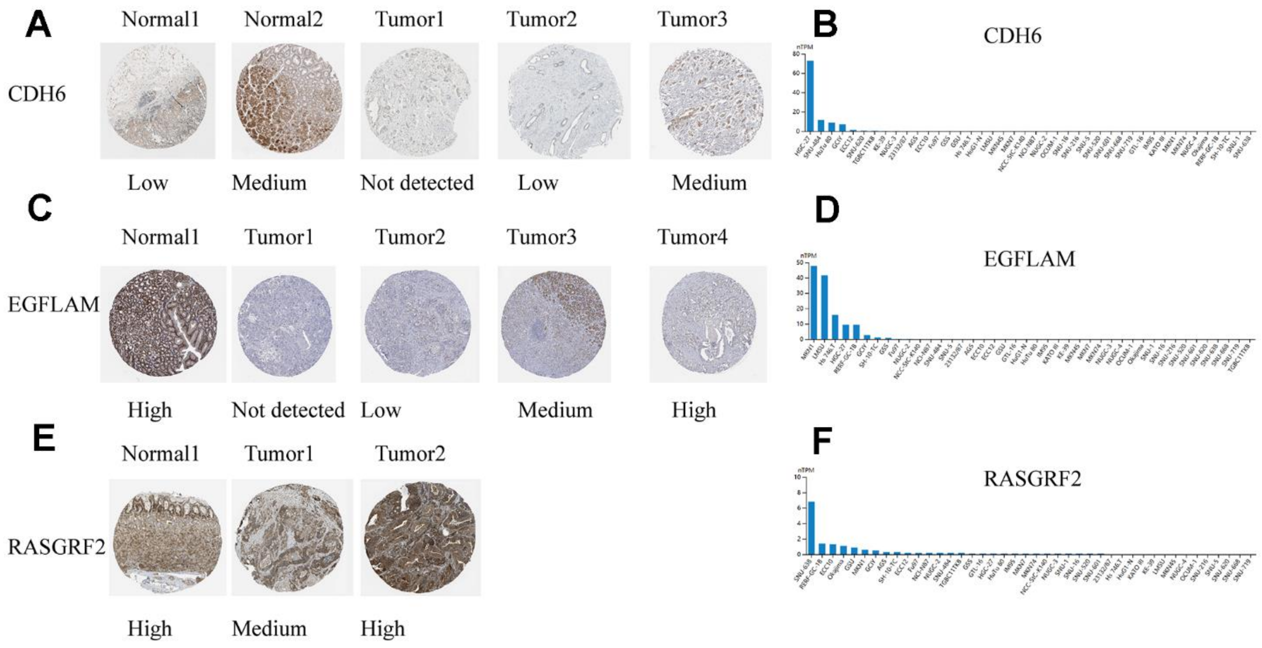

Supplementary Figure 7. Validation of hub genes expression levels. (**A**) Validation of protein expression levels of CDH6 in the HPA database. (**B**) Validation of CDH6 expression levels in gastric cancer cell lines using the CCLE database. (**C**) Validation of protein expression levels of EGFLAM in the HPA database. (**D**) Validation of EGFLAM expression levels in gastric cancer cell lines using the CCLE database. (**E**) Validation of protein expression levels of RASGRF2 in the HPA database. (**F**) Validation of RASGRF2 expression levels in gastric cancer cell lines using the CCLE database.

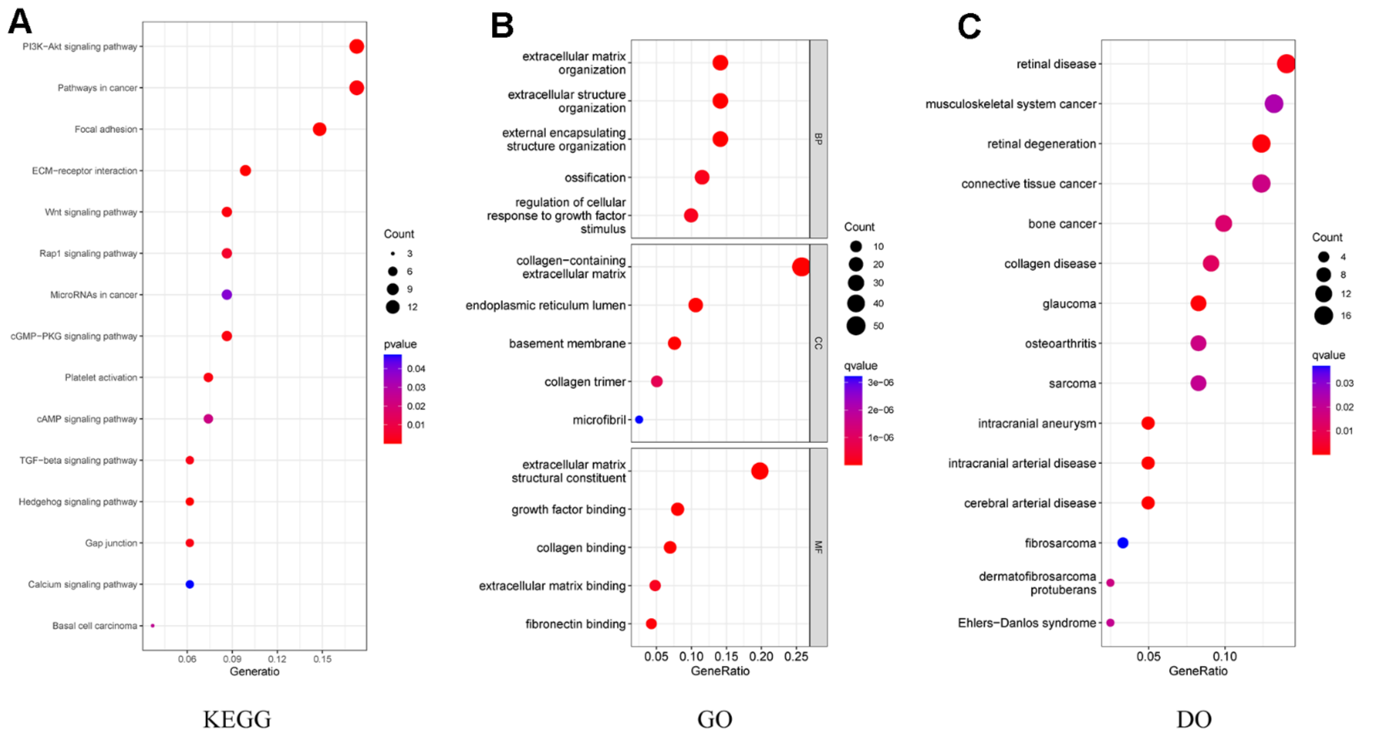

Supplementary Figure 8. Enrichment analysis of CAFs-related genes. (**A**) KEGG analysis of CAFs-related genes. (**B**) GO analysis of CAFs-related genes. (**C**) DO analysis of CAFs-related genes.
