# Supplementary material for: Integrated multi-omics analysis and machine learning identify hub genes and potential mechanisms of resistance to immunotherapy in gastric cancer
Source: Aging (Albany NY). 2024 Apr 22;16(8):7331–56. doi: 10.18632/aging.205760 (PMC11087130; doi:10.18632/aging.205760)
Supplement: Supplementary Table 1 [file aging-16-205760-s002.docx]

| Supplementary Table 1. 1136 differentially expressed genes obtained from the ICBatlas database. | | | | | |  |
| --- | --- | --- | --- | --- | --- | --- |
|  |  |  |  |  |  |  |
| **Gene Symbol** | **Response Mean** | **Non-Response Mean** | **Log2FC** | **FDR** | **P value** |  |
| KLK6 | 177 | 11 | 5.027 | 0 | 0 |  |
| MUC16 | 7 | 4 | 4.84 | 0 | 0 |  |
| HP | 16 | 31 | 4.766 | 0 | 0 |  |
| MAT1A | 51 | 5 | 4.7 | 0 | 0 |  |
| HPX | 21 | 13 | 4.514 | 0 | 0 |  |
| CALB2 | 37 | 8 | 4.503 | 0 | 0 |  |
| FGA | 152 | 76 | 4.386 | 0 | 0 |  |
| APOH | 44 | 8 | 4.343 | 0 | 0 |  |
| ITIH1 | 3 | 5 | 4.267 | 0 | 0 |  |
| FGB | 35 | 30 | 4.026 | 0 | 0 |  |
| KRT6A | 20 | 13 | 3.989 | 0 | 0 |  |
| TNFSF9 | 309 | 23 | 3.887 | 0 | 0 |  |
| KLK8 | 6 | 1 | 3.719 | 0 | 0 |  |
| CXCL11 | 831 | 134 | 3.68 | 0 | 0 |  |
| DIO1 | 20 | 3 | 3.442 | 0 | 0 |  |
| PLA2G2A | 913 | 123 | 3.39 | 0.01 | 0 |  |
| ALB | 70 | 107 | 3.375 | 0 | 0 |  |
| GPR87 | 19 | 5 | 3.34 | 0 | 0 |  |
| ITIH3 | 14 | 13 | 3.298 | 0 | 0 |  |
| KLK10 | 262 | 34 | 3.227 | 0 | 0 |  |
| SLC25A47 | 3 | 3 | 3.211 | 0.01 | 0 |  |
| FGG | 39 | 20 | 3.211 | 0.03 | 0.001 |  |
| IDO1 | 2,947 | 184 | 3.063 | 0 | 0 |  |
| LGALS17A | 27 | 3 | 3.045 | 0.01 | 0 |  |
| HAMP | 12 | 5 | 3.019 | 0 | 0 |  |
| UCA1 | 81 | 18 | 2.983 | 0.03 | 0.001 |  |
| CXCL9 | 3,925 | 582 | 2.94 | 0 | 0 |  |
| KRT6B | 71 | 14 | 2.92 | 0 | 0 |  |
| RP11-488P3.1 | 8 | 4 | 2.911 | 0 | 0 |  |
| CXCL10 | 2,976 | 444 | 2.899 | 0 | 0 |  |
| PRKCG | 78 | 12 | 2.889 | 0 | 0 |  |
| PADI1 | 16 | 3 | 2.854 | 0.04 | 0.002 |  |
| ORM1 | 71 | 65 | 2.853 | 0 | 0 |  |
| GBP5 | 4,130 | 670 | 2.746 | 0 | 0 |  |
| FGL1 | 20 | 7 | 2.703 | 0.04 | 0.002 |  |
| CYP2E1 | 57 | 37 | 2.674 | 0.03 | 0.001 |  |
| VSNL1 | 336 | 37 | 2.584 | 0 | 0 |  |
| TNNT1 | 394 | 16 | 2.572 | 0.03 | 0.002 |  |
| GBP7 | 3 | 3 | 2.518 | 0.02 | 0.001 |  |
| TNS4 | 780 | 130 | 2.511 | 0 | 0 |  |
| RP11-58O9.2 | 23 | 5 | 2.486 | 0.01 | 0 |  |
| RP3-460G2.2 | 4 | 2 | 2.481 | 0.01 | 0 |  |
| KRT80 | 575 | 47 | 2.479 | 0 | 0 |  |
| VTN | 21 | 33 | 2.475 | 0.05 | 0.003 |  |
| CYP2C8 | 16 | 13 | 2.444 | 0.02 | 0.001 |  |
| PTMAP5 | 30 | 13 | 2.432 | 0 | 0 |  |
| LINC01260 | 3 | 1 | 2.415 | 0.01 | 0 |  |
| GC | 265 | 125 | 2.409 | 0.04 | 0.002 |  |
| CXCR2P1 | 194 | 25 | 2.399 | 0 | 0 |  |
| FAM163B | 7 | 1 | 2.393 | 0.01 | 0 |  |
| NPSR1 | 36 | 11 | 2.369 | 0.05 | 0.003 |  |
| WARS | 13,436 | 3,592 | 2.334 | 0 | 0 |  |
| HAPLN3 | 1,404 | 329 | 2.317 | 0 | 0 |  |
| AIM2 | 172 | 62 | 2.317 | 0 | 0 |  |
| CAMK2N2 | 11 | 5 | 2.275 | 0.01 | 0 |  |
| DMKN | 1,764 | 235 | 2.266 | 0 | 0 |  |
| KLK7 | 42 | 8 | 2.25 | 0.04 | 0.002 |  |
| RAET1K | 24 | 2 | 2.21 | 0 | 0 |  |
| ARG1 | 11 | 7 | 2.195 | 0.01 | 0 |  |
| C2orf82 | 221 | 69 | 2.191 | 0 | 0 |  |
| AC002331.1 | 26 | 5 | 2.19 | 0 | 0 |  |
| HLA-G | 313 | 147 | 2.178 | 0 | 0 |  |
| SCEL | 117 | 35 | 2.178 | 0.05 | 0.003 |  |
| LINC01485 | 4 | 2 | 2.166 | 0.04 | 0.003 |  |
| PROX1-AS1 | 26 | 4 | 2.162 | 0.02 | 0 |  |
| LEMD1 | 49 | 9 | 2.155 | 0.02 | 0 |  |
| ACTBL2 | 7 | 2 | 2.115 | 0.03 | 0.001 |  |
| LAMC2 | 5,152 | 2,977 | 2.107 | 0.01 | 0 |  |
| OR2I1P | 4,773 | 629 | 2.075 | 0.01 | 0 |  |
| LYPD3 | 234 | 50 | 2.073 | 0 | 0 |  |
| SLC22A1 | 13 | 6 | 2.061 | 0.02 | 0.001 |  |
| CTB-50L17.14 | 6 | 2 | 2.047 | 0.02 | 0.001 |  |
| SIX4 | 157 | 60 | 2.046 | 0 | 0 |  |
| TFAP2A-AS1 | 30 | 7 | 2.045 | 0 | 0 |  |
| RP11-492E3.2 | 25 | 15 | 2.04 | 0.02 | 0.001 |  |
| IFNG | 109 | 16 | 2.014 | 0.01 | 0 |  |
| RP11-291B21.2 | 50 | 18 | 1.99 | 0.01 | 0 |  |
| GSDMC | 56 | 14 | 1.973 | 0.01 | 0 |  |
| UNC5A | 14 | 12 | 1.937 | 0.01 | 0 |  |
| BLACAT1 | 32 | 9 | 1.914 | 0.04 | 0.002 |  |
| UBD | 28 | 5 | 1.892 | 0.01 | 0 |  |
| GJB3 | 727 | 145 | 1.848 | 0.01 | 0 |  |
| SEMA3A | 113 | 110 | 1.846 | 0 | 0 |  |
| RP11-10J5.1 | 13 | 2 | 1.819 | 0.02 | 0.001 |  |
| SPX | 5 | 5 | 1.808 | 0.01 | 0 |  |
| SERPINA1 | 8,770 | 5,532 | 1.765 | 0.03 | 0.002 |  |
| RP11-351C21.2 | 9 | 4 | 1.755 | 0.01 | 0 |  |
| PMAIP1 | 707 | 158 | 1.753 | 0 | 0 |  |
| PLIN5 | 77 | 46 | 1.749 | 0.02 | 0.001 |  |
| FBXO39 | 9 | 4 | 1.739 | 0.04 | 0.002 |  |
| SYCE2 | 24 | 9 | 1.736 | 0 | 0 |  |
| LINC01554 | 3 | 2 | 1.73 | 0.05 | 0.003 |  |
| XKRX | 38 | 9 | 1.721 | 0 | 0 |  |
| SIK1 | 208 | 61 | 1.719 | 0.01 | 0 |  |
| CAPRIN2 | 675 | 355 | 1.709 | 0 | 0 |  |
| CCL18 | 1,748 | 305 | 1.701 | 0.03 | 0.002 |  |
| GZMB | 764 | 159 | 1.662 | 0.02 | 0.001 |  |
| FAM26F | 684 | 145 | 1.662 | 0 | 0 |  |
| ANKRD19P | 19 | 8 | 1.655 | 0.02 | 0.001 |  |
| KREMEN2 | 52 | 20 | 1.637 | 0.02 | 0.001 |  |
| ERICH2 | 21 | 16 | 1.618 | 0.03 | 0.001 |  |
| RP11-1069G10.1 | 6 | 5 | 1.613 | 0.03 | 0.001 |  |
| IL32 | 5,908 | 1,770 | 1.612 | 0 | 0 |  |
| FRMD5 | 318 | 80 | 1.611 | 0 | 0 |  |
| BATF2 | 974 | 287 | 1.603 | 0 | 0 |  |
| CD274 | 679 | 129 | 1.587 | 0.02 | 0.001 |  |
| RP11-122G18.11 | 6 | 3 | 1.585 | 0.02 | 0.001 |  |
| CTD-2288O8.1 | 13 | 3 | 1.58 | 0.02 | 0.001 |  |
| GBP4 | 4,726 | 1,596 | 1.568 | 0 | 0 |  |
| FOSL1 | 211 | 137 | 1.566 | 0.04 | 0.003 |  |
| FBXL16 | 122 | 37 | 1.566 | 0.01 | 0 |  |
| HLA-DQB1 | 6,505 | 2,717 | 1.545 | 0 | 0 |  |
| RARRES3 | 2,701 | 1,097 | 1.542 | 0 | 0 |  |
| HAS3 | 505 | 128 | 1.541 | 0.05 | 0.003 |  |
| BST2 | 7,647 | 1,698 | 1.541 | 0 | 0 |  |
| LINC00920 | 92 | 27 | 1.539 | 0 | 0 |  |
| RPL22L1 | 2,336 | 722 | 1.529 | 0 | 0 |  |
| RP4-694A7.2 | 4 | 2 | 1.524 | 0.05 | 0.003 |  |
| RP11-144G6.12 | 3 | 3 | 1.521 | 0.02 | 0.001 |  |
| RP11-248J18.2 | 25 | 13 | 1.519 | 0.01 | 0 |  |
| GBP1P1 | 246 | 65 | 1.491 | 0.01 | 0 |  |
| CTXN1 | 135 | 20 | 1.49 | 0.04 | 0.002 |  |
| KRT86 | 17 | 10 | 1.468 | 0.03 | 0.001 |  |
| RP11-567F11.1 | 5 | 1 | 1.466 | 0.03 | 0.001 |  |
| MT1F | 431 | 247 | 1.464 | 0 | 0 |  |
| GZMH | 331 | 111 | 1.457 | 0.01 | 0 |  |
| HSD17B6 | 71 | 77 | 1.443 | 0.03 | 0.002 |  |
| KCNRG | 7 | 4 | 1.437 | 0.03 | 0.001 |  |
| ZNF683 | 89 | 25 | 1.435 | 0.04 | 0.002 |  |
| ETV7 | 884 | 281 | 1.433 | 0 | 0 |  |
| RN7SL834P | 15 | 5 | 1.433 | 0.01 | 0 |  |
| MT1X | 491 | 264 | 1.431 | 0 | 0 |  |
| LY6E | 8,911 | 3,561 | 1.427 | 0 | 0 |  |
| IFI6 | 7,770 | 2,530 | 1.422 | 0.02 | 0.001 |  |
| AC007036.6 | 4 | 2 | 1.418 | 0.04 | 0.002 |  |
| MT2A | 4,774 | 1,627 | 1.394 | 0.02 | 0 |  |
| SDS | 390 | 163 | 1.387 | 0.05 | 0.003 |  |
| C2CD4C | 14 | 14 | 1.384 | 0.04 | 0.002 |  |
| IER5L | 1,003 | 356 | 1.369 | 0.01 | 0 |  |
| RP11-609D21.3 | 9 | 4 | 1.36 | 0.03 | 0.001 |  |
| SLC27A5 | 254 | 82 | 1.345 | 0.01 | 0 |  |
| SFN | 5,365 | 1,784 | 1.331 | 0.02 | 0.001 |  |
| TAP1 | 11,539 | 3,219 | 1.326 | 0 | 0 |  |
| IFITM10 | 14 | 8 | 1.326 | 0.01 | 0 |  |
| DOCK3 | 23 | 19 | 1.325 | 0.03 | 0.002 |  |
| ATF5 | 1,480 | 595 | 1.324 | 0.01 | 0 |  |
| IFITM1 | 4,734 | 1,514 | 1.321 | 0.02 | 0 |  |
| FXYD5 | 5,887 | 1,817 | 1.313 | 0 | 0 |  |
| PSMB9 | 5,739 | 1,407 | 1.303 | 0 | 0 |  |
| TYMP | 6,166 | 2,334 | 1.303 | 0.01 | 0 |  |
| USP30-AS1 | 53 | 22 | 1.301 | 0.05 | 0.003 |  |
| GBP1 | 6,682 | 1,999 | 1.3 | 0.01 | 0 |  |
| LAG3 | 354 | 117 | 1.295 | 0.01 | 0 |  |
| PCED1B | 209 | 117 | 1.286 | 0.02 | 0 |  |
| LRG1 | 1,420 | 946 | 1.283 | 0.01 | 0 |  |
| IFIT3 | 2,791 | 956 | 1.276 | 0.01 | 0 |  |
| SUSD4 | 255 | 117 | 1.267 | 0.05 | 0.003 |  |
| CYSRT1 | 58 | 18 | 1.262 | 0.03 | 0.002 |  |
| FOXD4 | 19 | 8 | 1.247 | 0 | 0 |  |
| RP11-267J23.4 | 18 | 9 | 1.245 | 0.05 | 0.003 |  |
| SMCO2 | 23 | 9 | 1.24 | 0.05 | 0.003 |  |
| NKG7 | 704 | 259 | 1.24 | 0.03 | 0.002 |  |
| PHLDA1 | 1,901 | 1,099 | 1.24 | 0.01 | 0 |  |
| CRTAM | 83 | 26 | 1.236 | 0.03 | 0.001 |  |
| EPHA2 | 3,631 | 1,341 | 1.204 | 0.01 | 0 |  |
| ANXA1 | 8,575 | 2,758 | 1.202 | 0.02 | 0.001 |  |
| KIAA0895 | 411 | 234 | 1.196 | 0.01 | 0 |  |
| MB21D1 | 855 | 322 | 1.194 | 0 | 0 |  |
| LMTK3 | 105 | 63 | 1.192 | 0.04 | 0.002 |  |
| ERRFI1 | 2,111 | 1,039 | 1.192 | 0.04 | 0.002 |  |
| PTMAP4 | 7 | 4 | 1.19 | 0.02 | 0 |  |
| CADM4 | 533 | 195 | 1.19 | 0.01 | 0 |  |
| PROSER2 | 254 | 258 | 1.187 | 0.02 | 0 |  |
| HLA-DRB1 | 16,605 | 7,987 | 1.184 | 0.02 | 0.001 |  |
| CENPH | 516 | 207 | 1.175 | 0 | 0 |  |
| FASLG | 128 | 53 | 1.173 | 0.02 | 0.001 |  |
| APOBEC3H | 54 | 21 | 1.171 | 0.04 | 0.002 |  |
| EPSTI1 | 3,613 | 896 | 1.168 | 0.03 | 0.002 |  |
| HAGHL | 243 | 97 | 1.165 | 0.04 | 0.002 |  |
| ATP8B3 | 42 | 35 | 1.159 | 0.03 | 0.002 |  |
| HIST2H2AC | 24 | 10 | 1.158 | 0 | 0 |  |
| CD8A | 1,034 | 393 | 1.145 | 0.05 | 0.003 |  |
| CDCP1 | 2,862 | 1,560 | 1.143 | 0.01 | 0 |  |
| CENPU | 814 | 307 | 1.14 | 0 | 0 |  |
| GCHFR | 153 | 46 | 1.134 | 0.01 | 0 |  |
| KRT18 | 22,879 | 12,438 | 1.133 | 0 | 0 |  |
| UBE2L6 | 4,926 | 1,587 | 1.119 | 0 | 0 |  |
| MIP | 6 | 2 | 1.111 | 0.03 | 0.001 |  |
| LAP3 | 5,748 | 2,651 | 1.101 | 0 | 0 |  |
| RP11-529E10.6 | 20 | 10 | 1.097 | 0.05 | 0.003 |  |
| STMN1 | 6,584 | 2,398 | 1.093 | 0 | 0 |  |
| STAT1 | 17,771 | 6,306 | 1.089 | 0.01 | 0 |  |
| AC011247.3 | 6 | 2 | 1.078 | 0.04 | 0.002 |  |
| FDXR | 817 | 247 | 1.076 | 0 | 0 |  |
| IRF1 | 4,719 | 1,877 | 1.063 | 0.02 | 0.001 |  |
| JAKMIP1 | 92 | 34 | 1.049 | 0.04 | 0.002 |  |
| LINC01572 | 20 | 8 | 1.045 | 0.03 | 0.002 |  |
| ARNTL2 | 2,207 | 838 | 1.024 | 0.01 | 0 |  |
| SLC9A7 | 944 | 522 | 1.016 | 0.01 | 0 |  |
| PLK2 | 1,155 | 943 | 1.014 | 0.01 | 0 |  |
| MND1 | 202 | 80 | 1.01 | 0 | 0 |  |
| FUZ | 110 | 202 | -1.004 | 0.05 | 0.003 |  |
| RASL12 | 133 | 209 | -1.006 | 0.02 | 0.001 |  |
| PLEKHH2 | 105 | 243 | -1.011 | 0.04 | 0.002 |  |
| MAMLD1 | 48 | 81 | -1.012 | 0.05 | 0.003 |  |
| MAGED1 | 1,667 | 3,265 | -1.013 | 0.01 | 0 |  |
| KCNN3 | 84 | 161 | -1.014 | 0.04 | 0.002 |  |
| HSPB8 | 157 | 272 | -1.018 | 0.05 | 0.003 |  |
| ADAMTS7 | 141 | 226 | -1.021 | 0.04 | 0.002 |  |
| SLC22A17 | 89 | 190 | -1.021 | 0.02 | 0.001 |  |
| CA14 | 19 | 40 | -1.021 | 0.02 | 0.001 |  |
| RGAG4 | 61 | 114 | -1.022 | 0.01 | 0 |  |
| SH3PXD2A | 870 | 1,693 | -1.024 | 0 | 0 |  |
| FAM126A | 282 | 484 | -1.025 | 0.01 | 0 |  |
| AFAP1L1 | 237 | 328 | -1.028 | 0.02 | 0.001 |  |
| STON2 | 106 | 137 | -1.029 | 0.03 | 0.002 |  |
| PLCB1 | 343 | 604 | -1.03 | 0.01 | 0 |  |
| HSPA12A | 52 | 104 | -1.032 | 0.03 | 0.001 |  |
| ZEB2 | 856 | 1,000 | -1.034 | 0.02 | 0.001 |  |
| MRVI1 | 363 | 627 | -1.037 | 0.01 | 0 |  |
| PDZD2 | 115 | 214 | -1.038 | 0.04 | 0.002 |  |
| PHLDB1 | 581 | 1,102 | -1.044 | 0.01 | 0 |  |
| CDH5 | 679 | 1,092 | -1.046 | 0.02 | 0.001 |  |
| NID1 | 1,489 | 2,874 | -1.047 | 0.01 | 0 |  |
| DAB2 | 869 | 1,101 | -1.047 | 0.02 | 0.001 |  |
| VWA7 | 92 | 163 | -1.047 | 0.03 | 0.002 |  |
| TCF7L1 | 64 | 143 | -1.05 | 0.02 | 0.001 |  |
| SGCE | 225 | 456 | -1.051 | 0.01 | 0 |  |
| PTPRB | 404 | 1,016 | -1.051 | 0.01 | 0 |  |
| AHRR | 34 | 81 | -1.053 | 0.04 | 0.002 |  |
| NRIP2 | 63 | 104 | -1.054 | 0 | 0 |  |
| ZNF470 | 59 | 92 | -1.057 | 0.04 | 0.002 |  |
| ADGRL2 | 560 | 1,089 | -1.057 | 0.01 | 0 |  |
| SNED1 | 91 | 190 | -1.058 | 0.01 | 0 |  |
| MEF2C | 302 | 582 | -1.059 | 0 | 0 |  |
| SPIRE1 | 182 | 263 | -1.059 | 0.02 | 0.001 |  |
| LRP1 | 2,193 | 4,481 | -1.06 | 0.05 | 0.003 |  |
| SLC16A2 | 193 | 334 | -1.061 | 0.04 | 0.002 |  |
| PDGFRB | 1,005 | 2,254 | -1.064 | 0.04 | 0.003 |  |
| KIAA1462 | 362 | 770 | -1.065 | 0.03 | 0.002 |  |
| DIP2C | 337 | 496 | -1.067 | 0.01 | 0 |  |
| CCT8P1 | 30 | 59 | -1.069 | 0.05 | 0.003 |  |
| MRGPRF | 70 | 164 | -1.072 | 0.04 | 0.003 |  |
| TBX2 | 326 | 525 | -1.074 | 0 | 0 |  |
| PCDH18 | 395 | 641 | -1.074 | 0.01 | 0 |  |
| PLXNA2 | 698 | 1,524 | -1.076 | 0.01 | 0 |  |
| KIF7 | 52 | 101 | -1.078 | 0.03 | 0.001 |  |
| RHOJ | 236 | 343 | -1.079 | 0.01 | 0 |  |
| GOLIM4 | 1,488 | 2,416 | -1.079 | 0.02 | 0.001 |  |
| EHD2 | 640 | 1,421 | -1.08 | 0.01 | 0 |  |
| RASGRF2 | 151 | 225 | -1.08 | 0.01 | 0 |  |
| HENMT1 | 148 | 444 | -1.08 | 0.02 | 0.001 |  |
| CPNE8 | 171 | 279 | -1.081 | 0.01 | 0 |  |
| CPED1 | 241 | 402 | -1.082 | 0.03 | 0.001 |  |
| CYGB | 309 | 555 | -1.083 | 0.04 | 0.002 |  |
| CNTNAP1 | 92 | 199 | -1.083 | 0.01 | 0 |  |
| ACKR3 | 323 | 489 | -1.085 | 0.04 | 0.003 |  |
| SHANK3 | 269 | 619 | -1.086 | 0.04 | 0.002 |  |
| PARD3B | 305 | 463 | -1.09 | 0.04 | 0.002 |  |
| OLFML3 | 371 | 475 | -1.096 | 0.05 | 0.003 |  |
| RAMP2 | 310 | 424 | -1.098 | 0.04 | 0.003 |  |
| AOC3 | 246 | 545 | -1.099 | 0.03 | 0.001 |  |
| DISP1 | 146 | 311 | -1.1 | 0.01 | 0 |  |
| PAMR1 | 110 | 227 | -1.102 | 0 | 0 |  |
| GJC1 | 172 | 282 | -1.102 | 0.03 | 0.001 |  |
| TMTC1 | 200 | 364 | -1.106 | 0.01 | 0 |  |
| PRR29 | 37 | 46 | -1.108 | 0.03 | 0.001 |  |
| AC144831.1 | 14 | 20 | -1.108 | 0.05 | 0.003 |  |
| RAI2 | 119 | 250 | -1.109 | 0.02 | 0.001 |  |
| KIF5C | 45 | 88 | -1.111 | 0.04 | 0.003 |  |
| PXDN | 1,110 | 2,020 | -1.111 | 0.03 | 0.002 |  |
| FEZ1 | 116 | 199 | -1.114 | 0.01 | 0 |  |
| ARHGEF17 | 417 | 809 | -1.114 | 0.01 | 0 |  |
| ZNF649 | 94 | 160 | -1.116 | 0.03 | 0.001 |  |
| RCAN2 | 222 | 453 | -1.12 | 0.02 | 0.001 |  |
| ZNF549 | 75 | 161 | -1.12 | 0.01 | 0 |  |
| CXorf36 | 237 | 369 | -1.122 | 0.02 | 0.001 |  |
| CRACR2A | 93 | 267 | -1.123 | 0.02 | 0.001 |  |
| RASL11A | 110 | 243 | -1.124 | 0.01 | 0 |  |
| LINGO1 | 67 | 104 | -1.124 | 0.04 | 0.003 |  |
| HCG11 | 161 | 371 | -1.126 | 0.03 | 0.002 |  |
| SEMA5A | 232 | 470 | -1.127 | 0 | 0 |  |
| KL | 35 | 92 | -1.131 | 0.02 | 0.001 |  |
| HGD | 508 | 739 | -1.131 | 0.04 | 0.002 |  |
| MCAM | 1,642 | 2,408 | -1.137 | 0.01 | 0 |  |
| RFTN2 | 67 | 116 | -1.148 | 0.01 | 0 |  |
| EDNRB | 319 | 583 | -1.15 | 0.01 | 0 |  |
| SLC15A2 | 47 | 80 | -1.156 | 0.01 | 0 |  |
| SYNPO | 1,320 | 2,376 | -1.159 | 0.01 | 0 |  |
| EMID1 | 229 | 392 | -1.159 | 0.02 | 0.001 |  |
| SESN3 | 269 | 573 | -1.167 | 0.01 | 0 |  |
| ZNF518B | 157 | 324 | -1.167 | 0.01 | 0 |  |
| SGIP1 | 81 | 150 | -1.168 | 0.04 | 0.003 |  |
| SVIL | 833 | 1,489 | -1.17 | 0.01 | 0 |  |
| ZBTB20 | 39 | 90 | -1.174 | 0.03 | 0.002 |  |
| PIR | 281 | 427 | -1.177 | 0.01 | 0 |  |
| THBD | 519 | 807 | -1.181 | 0.01 | 0 |  |
| NOV | 57 | 117 | -1.182 | 0.03 | 0.002 |  |
| DAAM2 | 148 | 319 | -1.182 | 0.01 | 0 |  |
| HGF | 179 | 243 | -1.185 | 0.02 | 0.001 |  |
| FRZB | 152 | 331 | -1.186 | 0.05 | 0.003 |  |
| RASSF8 | 152 | 219 | -1.186 | 0.02 | 0.001 |  |
| C14orf37 | 34 | 88 | -1.186 | 0.01 | 0 |  |
| CC2D2B | 15 | 21 | -1.188 | 0.05 | 0.003 |  |
| LRRC70 | 10 | 16 | -1.19 | 0.02 | 0 |  |
| ZNF215 | 23 | 74 | -1.19 | 0.02 | 0.001 |  |
| NOTCH3 | 757 | 1,865 | -1.192 | 0.05 | 0.003 |  |
| C5orf42 | 156 | 366 | -1.193 | 0.04 | 0.002 |  |
| EDIL3 | 542 | 1,172 | -1.193 | 0.01 | 0 |  |
| NPR1 | 125 | 174 | -1.2 | 0.02 | 0.001 |  |
| PLD1 | 585 | 890 | -1.2 | 0 | 0 |  |
| IL34 | 74 | 105 | -1.202 | 0.03 | 0.001 |  |
| PRKG1 | 129 | 301 | -1.208 | 0 | 0 |  |
| FBXL7 | 94 | 151 | -1.211 | 0.01 | 0 |  |
| SYNE3 | 160 | 276 | -1.211 | 0.01 | 0 |  |
| NEURL1B | 346 | 764 | -1.212 | 0.01 | 0 |  |
| TRIL | 68 | 181 | -1.212 | 0.04 | 0.002 |  |
| RECK | 130 | 247 | -1.213 | 0.01 | 0 |  |
| CD34 | 875 | 1,357 | -1.213 | 0.01 | 0 |  |
| PEAR1 | 125 | 218 | -1.213 | 0.01 | 0 |  |
| CD93 | 1,710 | 2,235 | -1.214 | 0.02 | 0.001 |  |
| LEF1 | 137 | 211 | -1.214 | 0.01 | 0 |  |
| CD109 | 195 | 487 | -1.215 | 0.03 | 0.001 |  |
| C22orf34 | 18 | 38 | -1.216 | 0.02 | 0.001 |  |
| TCF4 | 751 | 1,441 | -1.217 | 0 | 0 |  |
| ITGB3 | 50 | 88 | -1.219 | 0.03 | 0.001 |  |
| GJB1 | 460 | 1,030 | -1.22 | 0.01 | 0 |  |
| GPRASP1 | 58 | 127 | -1.22 | 0.02 | 0.001 |  |
| LGI2 | 46 | 96 | -1.221 | 0.03 | 0.001 |  |
| SOGA1 | 257 | 669 | -1.223 | 0 | 0 |  |
| WNT2B | 90 | 136 | -1.223 | 0.02 | 0.001 |  |
| PCDHGB7 | 31 | 46 | -1.231 | 0.01 | 0 |  |
| OSBP2 | 68 | 127 | -1.232 | 0.04 | 0.002 |  |
| PRPH2 | 16 | 39 | -1.232 | 0.03 | 0.001 |  |
| NHSL2 | 116 | 214 | -1.234 | 0.01 | 0 |  |
| COL6A1 | 3,506 | 4,597 | -1.235 | 0.02 | 0 |  |
| PTCH1 | 380 | 729 | -1.238 | 0.01 | 0 |  |
| RUNDC3B | 23 | 37 | -1.239 | 0.05 | 0.003 |  |
| ZNF605 | 136 | 364 | -1.239 | 0 | 0 |  |
| FGD5 | 250 | 690 | -1.24 | 0 | 0 |  |
| PYGO1 | 46 | 91 | -1.24 | 0.01 | 0 |  |
| NETO2 | 117 | 208 | -1.252 | 0.05 | 0.003 |  |
| CEP126 | 42 | 101 | -1.252 | 0.03 | 0.001 |  |
| TTC28 | 163 | 395 | -1.256 | 0 | 0 |  |
| EGFLAM | 70 | 131 | -1.258 | 0.01 | 0 |  |
| KLHL30 | 9 | 17 | -1.259 | 0.05 | 0.003 |  |
| MGAT3 | 357 | 806 | -1.259 | 0.04 | 0.002 |  |
| PCDHGA9 | 13 | 24 | -1.261 | 0.01 | 0 |  |
| IGDCC4 | 72 | 152 | -1.262 | 0 | 0 |  |
| SLFN13 | 261 | 731 | -1.264 | 0.01 | 0 |  |
| MATN2 | 397 | 1,516 | -1.265 | 0.02 | 0.001 |  |
| NTF3 | 13 | 26 | -1.266 | 0.01 | 0 |  |
| TBX2-AS1 | 14 | 22 | -1.267 | 0.04 | 0.002 |  |
| ZC3H12B | 34 | 75 | -1.268 | 0 | 0 |  |
| SLC4A10 | 8 | 16 | -1.268 | 0.04 | 0.002 |  |
| TATDN2P2 | 22 | 45 | -1.271 | 0.01 | 0 |  |
| TEK | 259 | 503 | -1.272 | 0.01 | 0 |  |
| CSGALNACT1 | 299 | 505 | -1.273 | 0 | 0 |  |
| ME1 | 348 | 1,117 | -1.273 | 0.04 | 0.002 |  |
| PTGS1 | 536 | 1,060 | -1.275 | 0.01 | 0 |  |
| CACNB2 | 25 | 41 | -1.276 | 0.05 | 0.003 |  |
| CASC15 | 39 | 89 | -1.281 | 0.04 | 0.002 |  |
| NAV1 | 346 | 720 | -1.281 | 0 | 0 |  |
| PLAGL1 | 189 | 371 | -1.284 | 0 | 0 |  |
| ZNF736 | 122 | 243 | -1.284 | 0.03 | 0.001 |  |
| CNTNAP3P2 | 6 | 14 | -1.285 | 0.03 | 0.001 |  |
| GJA1 | 761 | 1,453 | -1.289 | 0.01 | 0 |  |
| LINC01415 | 4 | 7 | -1.29 | 0.05 | 0.003 |  |
| KDR | 499 | 1,264 | -1.29 | 0.01 | 0 |  |
| NCKAP5 | 31 | 80 | -1.291 | 0.03 | 0.002 |  |
| PARM1 | 337 | 987 | -1.295 | 0.02 | 0.001 |  |
| CYBRD1 | 1,144 | 1,824 | -1.296 | 0.04 | 0.003 |  |
| SH3RF3 | 128 | 238 | -1.296 | 0 | 0 |  |
| PLA2R1 | 99 | 237 | -1.297 | 0.02 | 0.001 |  |
| CACNA1C | 86 | 237 | -1.298 | 0.01 | 0 |  |
| CDKL1 | 20 | 43 | -1.298 | 0 | 0 |  |
| CSPG4 | 210 | 423 | -1.299 | 0 | 0 |  |
| KIRREL | 442 | 842 | -1.299 | 0 | 0 |  |
| ZNF853 | 63 | 135 | -1.301 | 0.01 | 0 |  |
| SLC2A4 | 17 | 33 | -1.302 | 0.04 | 0.002 |  |
| SYNPO2 | 414 | 1,007 | -1.304 | 0.02 | 0.001 |  |
| DUOXA1 | 20 | 51 | -1.305 | 0.02 | 0.001 |  |
| AKR1C3 | 3,273 | 6,205 | -1.308 | 0.03 | 0.001 |  |
| CSRNP3 | 13 | 49 | -1.313 | 0.03 | 0.002 |  |
| ZNF512B | 283 | 433 | -1.314 | 0.01 | 0 |  |
| ROBO1 | 403 | 708 | -1.314 | 0 | 0 |  |
| EMCN | 345 | 356 | -1.316 | 0.02 | 0.001 |  |
| EFHC2 | 16 | 52 | -1.318 | 0.03 | 0.002 |  |
| ACTRT3 | 22 | 48 | -1.319 | 0 | 0 |  |
| PRDM5 | 63 | 123 | -1.32 | 0.01 | 0 |  |
| FILIP1 | 69 | 133 | -1.321 | 0 | 0 |  |
| THSD7A | 46 | 105 | -1.323 | 0.01 | 0 |  |
| VASH2 | 31 | 72 | -1.325 | 0.01 | 0 |  |
| PTGDS | 28 | 53 | -1.326 | 0.04 | 0.002 |  |
| ZNF256 | 37 | 71 | -1.327 | 0.01 | 0 |  |
| NRG2 | 5 | 9 | -1.327 | 0.03 | 0.001 |  |
| CUBN | 22 | 43 | -1.329 | 0.01 | 0 |  |
| NDRG1 | 4,490 | 9,600 | -1.332 | 0 | 0 |  |
| COL6A2 | 4,328 | 5,458 | -1.333 | 0.02 | 0.001 |  |
| FAM188B | 14 | 33 | -1.333 | 0.02 | 0.001 |  |
| BICC1 | 134 | 271 | -1.335 | 0.04 | 0.002 |  |
| ZNF530 | 35 | 89 | -1.336 | 0 | 0 |  |
| ANGPT1 | 86 | 170 | -1.337 | 0 | 0 |  |
| KCNAB1 | 30 | 55 | -1.338 | 0.01 | 0 |  |
| FAM13C | 41 | 94 | -1.34 | 0.01 | 0 |  |
| NID2 | 200 | 323 | -1.341 | 0.03 | 0.001 |  |
| LHX6 | 67 | 115 | -1.346 | 0.04 | 0.002 |  |
| FAM65C | 88 | 165 | -1.347 | 0.01 | 0 |  |
| VSTM4 | 105 | 242 | -1.348 | 0.02 | 0.001 |  |
| KLHL13 | 40 | 117 | -1.35 | 0.01 | 0 |  |
| BTBD11 | 26 | 59 | -1.35 | 0.04 | 0.002 |  |
| AKR1E2 | 17 | 44 | -1.356 | 0.02 | 0.001 |  |
| COL15A1 | 2,012 | 3,243 | -1.356 | 0.04 | 0.002 |  |
| CTD-2334D19.1 | 8 | 15 | -1.357 | 0.04 | 0.002 |  |
| FAT2 | 4 | 8 | -1.358 | 0.05 | 0.003 |  |
| TSPAN18 | 219 | 358 | -1.358 | 0.01 | 0 |  |
| GLI1 | 33 | 74 | -1.358 | 0.05 | 0.003 |  |
| RP11-14N7.2 | 4 | 13 | -1.362 | 0.03 | 0.002 |  |
| LPL | 76 | 155 | -1.363 | 0.04 | 0.002 |  |
| CDH6 | 118 | 226 | -1.364 | 0.01 | 0 |  |
| OLFML1 | 95 | 178 | -1.364 | 0.01 | 0 |  |
| TRPC6 | 76 | 146 | -1.366 | 0.01 | 0 |  |
| ZNF154 | 41 | 71 | -1.367 | 0 | 0 |  |
| LHFP | 397 | 692 | -1.367 | 0.02 | 0.001 |  |
| DDR2 | 350 | 718 | -1.37 | 0.01 | 0 |  |
| MAP1B | 256 | 546 | -1.372 | 0.03 | 0.002 |  |
| ADAM23 | 37 | 87 | -1.375 | 0.01 | 0 |  |
| CTD-2554C21.2 | 7 | 17 | -1.383 | 0.04 | 0.002 |  |
| BANK1 | 73 | 142 | -1.385 | 0.05 | 0.003 |  |
| ITGA7 | 149 | 339 | -1.385 | 0 | 0 |  |
| SOBP | 40 | 121 | -1.386 | 0.01 | 0 |  |
| LAMA4 | 1,200 | 2,358 | -1.388 | 0 | 0 |  |
| ITGA8 | 157 | 330 | -1.388 | 0.02 | 0.001 |  |
| LINC00654 | 44 | 89 | -1.394 | 0 | 0 |  |
| DCHS1 | 228 | 597 | -1.396 | 0 | 0 |  |
| FSTL1 | 3,678 | 6,140 | -1.397 | 0.01 | 0 |  |
| ADGRF5 | 775 | 1,351 | -1.401 | 0.01 | 0 |  |
| RP11-54O7.3 | 18 | 50 | -1.404 | 0.04 | 0.002 |  |
| LRRC17 | 32 | 65 | -1.407 | 0.04 | 0.002 |  |
| LGALS2 | 115 | 219 | -1.413 | 0.01 | 0 |  |
| RSPH4A | 7 | 10 | -1.417 | 0.04 | 0.003 |  |
| PAPLN | 126 | 367 | -1.422 | 0 | 0 |  |
| LRRFIP1P1 | 10 | 22 | -1.436 | 0.04 | 0.002 |  |
| PRICKLE1 | 53 | 91 | -1.44 | 0.03 | 0.001 |  |
| SALL2 | 36 | 91 | -1.441 | 0.01 | 0 |  |
| SOX6 | 83 | 123 | -1.441 | 0.03 | 0.001 |  |
| NMNAT2 | 26 | 49 | -1.444 | 0.04 | 0.002 |  |
| EML1 | 169 | 419 | -1.449 | 0 | 0 |  |
| NEK10 | 11 | 23 | -1.45 | 0.04 | 0.002 |  |
| COL23A1 | 53 | 132 | -1.453 | 0 | 0 |  |
| SLCO2A1 | 332 | 1,196 | -1.454 | 0.03 | 0.002 |  |
| SV2A | 33 | 80 | -1.454 | 0 | 0 |  |
| ACHE | 36 | 178 | -1.455 | 0.03 | 0.001 |  |
| CD1C | 20 | 38 | -1.459 | 0.05 | 0.003 |  |
| PRUNE2 | 151 | 437 | -1.46 | 0.02 | 0.001 |  |
| TCEAL7 | 28 | 49 | -1.463 | 0.03 | 0.001 |  |
| REM1 | 20 | 30 | -1.463 | 0.01 | 0 |  |
| EVC2 | 35 | 61 | -1.465 | 0.02 | 0.001 |  |
| LAMC3 | 145 | 226 | -1.47 | 0.03 | 0.002 |  |
| C2CD4B | 71 | 190 | -1.471 | 0.03 | 0.002 |  |
| CDH13 | 276 | 534 | -1.471 | 0.01 | 0 |  |
| ANO2 | 16 | 36 | -1.472 | 0.01 | 0 |  |
| CDH23 | 28 | 70 | -1.472 | 0.01 | 0 |  |
| COL9A3 | 34 | 118 | -1.476 | 0.02 | 0 |  |
| PDE7B | 51 | 111 | -1.48 | 0 | 0 |  |
| MMP24 | 31 | 32 | -1.48 | 0.04 | 0.002 |  |
| ECM2 | 163 | 323 | -1.481 | 0.02 | 0.001 |  |
| ZNF813 | 41 | 190 | -1.483 | 0.02 | 0.001 |  |
| LSAMP | 74 | 189 | -1.487 | 0.01 | 0 |  |
| TUBB2B | 15 | 23 | -1.487 | 0.03 | 0.002 |  |
| SCN4B | 52 | 95 | -1.488 | 0.03 | 0.002 |  |
| BDNF | 16 | 28 | -1.49 | 0.01 | 0 |  |
| CH25H | 23 | 39 | -1.494 | 0.01 | 0 |  |
| CRYM | 21 | 60 | -1.494 | 0.04 | 0.002 |  |
| RP11-266K4.9 | 5 | 13 | -1.496 | 0.01 | 0 |  |
| CCDC85A | 3 | 11 | -1.497 | 0.02 | 0.001 |  |
| DNM3 | 61 | 162 | -1.498 | 0.01 | 0 |  |
| CTD-2017D11.1 | 17 | 26 | -1.505 | 0.02 | 0.001 |  |
| LPAR1 | 193 | 396 | -1.507 | 0.01 | 0 |  |
| ASAH2 | 36 | 48 | -1.509 | 0.04 | 0.002 |  |
| AHNAK | 11,579 | 17,792 | -1.511 | 0 | 0 |  |
| CORO2B | 16 | 31 | -1.516 | 0.01 | 0 |  |
| PCDHGB5 | 15 | 29 | -1.519 | 0.05 | 0.003 |  |
| HTR2A | 6 | 14 | -1.52 | 0.05 | 0.003 |  |
| CDH26 | 14 | 27 | -1.524 | 0.02 | 0.001 |  |
| NAALADL1 | 68 | 124 | -1.524 | 0.01 | 0 |  |
| RP11-673E1.3 | 3 | 5 | -1.525 | 0.03 | 0.002 |  |
| CCDC181 | 14 | 23 | -1.53 | 0.03 | 0.001 |  |
| SHISA2 | 41 | 93 | -1.532 | 0.03 | 0.002 |  |
| DTNA | 53 | 123 | -1.533 | 0.03 | 0.001 |  |
| ARHGAP6 | 68 | 165 | -1.533 | 0 | 0 |  |
| CLDN11 | 30 | 49 | -1.537 | 0.05 | 0.003 |  |
| ANKRD55 | 8 | 8 | -1.538 | 0.04 | 0.002 |  |
| EPHA3 | 125 | 140 | -1.539 | 0.04 | 0.002 |  |
| GRID1 | 14 | 43 | -1.539 | 0.02 | 0.001 |  |
| DCLK1 | 40 | 73 | -1.54 | 0.05 | 0.003 |  |
| PRRX2 | 47 | 90 | -1.541 | 0.05 | 0.003 |  |
| CDHR2 | 193 | 734 | -1.542 | 0.04 | 0.002 |  |
| CLMP | 199 | 389 | -1.542 | 0.02 | 0.001 |  |
| GUCY1A2 | 83 | 167 | -1.543 | 0 | 0 |  |
| PDE3A | 77 | 155 | -1.549 | 0.01 | 0 |  |
| PDGFRA | 715 | 2,048 | -1.55 | 0 | 0 |  |
| SCUBE2 | 23 | 65 | -1.553 | 0.02 | 0.001 |  |
| PCDHB7 | 11 | 21 | -1.554 | 0.05 | 0.003 |  |
| TRPC4 | 19 | 50 | -1.555 | 0 | 0 |  |
| LINC01537 | 4 | 5 | -1.556 | 0.03 | 0.001 |  |
| AC007743.1 | 7 | 20 | -1.558 | 0.04 | 0.002 |  |
| MYOCD | 47 | 149 | -1.558 | 0.02 | 0.001 |  |
| SMOC2 | 246 | 506 | -1.566 | 0.03 | 0.001 |  |
| TEX11 | 15 | 25 | -1.568 | 0.04 | 0.002 |  |
| LRRTM2 | 6 | 14 | -1.571 | 0.03 | 0.001 |  |
| PCDHGC3 | 72 | 241 | -1.572 | 0 | 0 |  |
| PREX2 | 100 | 306 | -1.575 | 0 | 0 |  |
| TSPAN11 | 99 | 242 | -1.576 | 0.02 | 0.001 |  |
| KCNF1 | 3 | 7 | -1.579 | 0.03 | 0.001 |  |
| GSTM5 | 11 | 44 | -1.581 | 0.03 | 0.002 |  |
| TUB | 25 | 80 | -1.584 | 0.03 | 0.001 |  |
| UNC5C | 85 | 207 | -1.585 | 0.01 | 0 |  |
| SEMA5B | 27 | 60 | -1.587 | 0 | 0 |  |
| SEMA6A | 109 | 284 | -1.588 | 0.03 | 0.001 |  |
| HHIP-AS1 | 12 | 28 | -1.59 | 0.02 | 0.001 |  |
| CGNL1 | 239 | 249 | -1.593 | 0.05 | 0.003 |  |
| PIEZO2 | 99 | 264 | -1.594 | 0 | 0 |  |
| SPINK5 | 102 | 268 | -1.6 | 0.03 | 0.001 |  |
| OLFML2B | 270 | 403 | -1.6 | 0.04 | 0.002 |  |
| HOXA2 | 14 | 28 | -1.605 | 0.03 | 0.001 |  |
| GYPE | 4 | 6 | -1.607 | 0.05 | 0.003 |  |
| VGLL3 | 60 | 76 | -1.609 | 0.03 | 0.002 |  |
| CTTNBP2 | 27 | 63 | -1.611 | 0.01 | 0 |  |
| CYTL1 | 18 | 37 | -1.611 | 0.02 | 0.001 |  |
| SEMA6D | 58 | 150 | -1.613 | 0.01 | 0 |  |
| CD248 | 179 | 441 | -1.615 | 0.02 | 0.001 |  |
| SELP | 75 | 223 | -1.616 | 0.03 | 0.001 |  |
| ZNF521 | 105 | 243 | -1.617 | 0 | 0 |  |
| PDE1C | 63 | 170 | -1.617 | 0.02 | 0.001 |  |
| PLA2G4D | 4 | 9 | -1.618 | 0.03 | 0.002 |  |
| GLIS1 | 7 | 17 | -1.62 | 0.01 | 0 |  |
| HIST1H4H | 59 | 123 | -1.621 | 0 | 0 |  |
| SYPL2 | 11 | 23 | -1.622 | 0.04 | 0.003 |  |
| FGF12 | 16 | 21 | -1.624 | 0.03 | 0.001 |  |
| PABPC4L | 15 | 31 | -1.625 | 0 | 0 |  |
| LINC01197 | 19 | 29 | -1.626 | 0.03 | 0.002 |  |
| SLC5A4 | 5 | 18 | -1.626 | 0.04 | 0.003 |  |
| ENPP6 | 11 | 34 | -1.629 | 0.03 | 0.001 |  |
| PCDHB4 | 13 | 41 | -1.631 | 0 | 0 |  |
| MACROD2 | 42 | 100 | -1.633 | 0.01 | 0 |  |
| LINC01091 | 28 | 47 | -1.634 | 0.02 | 0.001 |  |
| PODN | 147 | 358 | -1.635 | 0.01 | 0 |  |
| PRSS35 | 10 | 20 | -1.635 | 0.04 | 0.003 |  |
| MFAP4 | 464 | 1,606 | -1.637 | 0.01 | 0 |  |
| KCNN1 | 5 | 7 | -1.639 | 0.04 | 0.002 |  |
| OLFML2A | 154 | 445 | -1.643 | 0 | 0 |  |
| ZNF541 | 5 | 8 | -1.644 | 0.04 | 0.002 |  |
| CAPN11 | 9 | 21 | -1.647 | 0.03 | 0.002 |  |
| C1QTNF2 | 23 | 38 | -1.647 | 0.01 | 0 |  |
| RP11-297L17.2 | 6 | 19 | -1.647 | 0.02 | 0.001 |  |
| ADD3-AS1 | 8 | 10 | -1.648 | 0.03 | 0.002 |  |
| VWF | 1,326 | 4,702 | -1.649 | 0.01 | 0 |  |
| FBLN5 | 266 | 623 | -1.649 | 0 | 0 |  |
| RP11-43F13.3 | 3 | 7 | -1.65 | 0.02 | 0.001 |  |
| RBP2 | 3 | 8 | -6.716 | 0 | 0 |  |
| NRK | 2 | 6 | -4.704 | 0 | 0 |  |
| MALRD1 | 2 | 10 | -4.933 | 0 | 0 |  |
| LTF | 187 | 597 | -5.081 | 0 | 0 |  |
| TNNT3 | 2 | 4 | -5.143 | 0 | 0 |  |
| GAST | 5 | 6 | -5.673 | 0.01 | 0 |  |
| LCT | 1 | 2 | -4.452 | 0 | 0 |  |
| PI16 | 19 | 26 | -4.473 | 0 | 0 |  |
| REG3A | 81 | 264 | -4.516 | 0 | 0 |  |
| NTS | 2 | 11 | -4.594 | 0 | 0 |  |
| COMP | 8 | 17 | -4.677 | 0 | 0 |  |
| ALPI | 2 | 21 | -4.169 | 0 | 0 |  |
| SCN2A | 6 | 14 | -4.324 | 0 | 0 |  |
| CEACAM20 | 0 | 9 | -4.326 | 0 | 0 |  |
| TMPRSS15 | 3 | 4 | -4.331 | 0 | 0 |  |
| CA1 | 3 | 12 | -4.448 | 0 | 0 |  |
| A2ML1 | 2 | 3 | -4.071 | 0 | 0 |  |
| FGF19 | 1 | 3 | -4.072 | 0 | 0 |  |
| CSAG1 | 3 | 2 | -4.106 | 0 | 0 |  |
| MEP1B | 3 | 11 | -4.123 | 0 | 0 |  |
| MMP13 | 4 | 8 | -4.154 | 0 | 0 |  |
| CASR | 2 | 4 | -3.874 | 0 | 0 |  |
| SFRP2 | 21 | 29 | -3.89 | 0 | 0 |  |
| MB | 6 | 4 | -3.93 | 0 | 0 |  |
| CAMKV | 1 | 2 | -3.989 | 0.01 | 0 |  |
| FAM178B | 1 | 3 | -4.02 | 0 | 0 |  |
| COL2A1 | 3 | 8 | -3.799 | 0.01 | 0 |  |
| UCN3 | 1 | 13 | -3.862 | 0 | 0 |  |
| AC018890.6 | 1 | 3 | -3.868 | 0 | 0 |  |
| MYBPC1 | 9 | 27 | -3.868 | 0 | 0 |  |
| LHFPL3 | 2 | 9 | -3.87 | 0 | 0 |  |
| SLC6A4 | 3 | 9 | -3.707 | 0 | 0 |  |
| NTRK2 | 21 | 45 | -3.761 | 0 | 0 |  |
| SI | 12 | 40 | -3.761 | 0.02 | 0.001 |  |
| PLG | 2 | 3 | -3.768 | 0.01 | 0 |  |
| ACTA1 | 3 | 2 | -3.784 | 0.02 | 0.001 |  |
| KIF25-AS1 | 1 | 4 | -3.58 | 0.01 | 0 |  |
| OGN | 12 | 37 | -3.632 | 0 | 0 |  |
| ISX | 2 | 9 | -3.666 | 0.01 | 0 |  |
| CA4 | 7 | 51 | -3.681 | 0 | 0 |  |
| IGFN1 | 3 | 4 | -3.701 | 0.01 | 0 |  |
| SLC3A1 | 11 | 85 | -3.53 | 0 | 0 |  |
| GJB7 | 1 | 2 | -3.546 | 0 | 0 |  |
| CRABP2 | 33 | 151 | -3.568 | 0 | 0 |  |
| NPY6R | 3 | 3 | -3.571 | 0.01 | 0 |  |
| KRT1 | 2 | 2 | -3.577 | 0.01 | 0 |  |
| LEFTY1 | 5 | 12 | -3.486 | 0.01 | 0 |  |
| GDF10 | 2 | 3 | -3.503 | 0.01 | 0 |  |
| TRPM6 | 15 | 44 | -3.51 | 0 | 0 |  |
| PCSK2 | 1 | 7 | -3.515 | 0.01 | 0 |  |
| DPY19L2P1 | 2 | 4 | -3.517 | 0 | 0 |  |
| TRIM54 | 2 | 46 | -3.446 | 0 | 0 |  |
| XPNPEP2 | 9 | 45 | -3.447 | 0 | 0 |  |
| DSG1 | 2 | 1 | -3.466 | 0.01 | 0 |  |
| PHACTR3 | 6 | 25 | -3.47 | 0 | 0 |  |
| DRD2 | 1 | 2 | -3.481 | 0 | 0 |  |
| SPINK4 | 11 | 38 | -3.361 | 0 | 0 |  |
| NOC2LP1 | 2 | 3 | -3.364 | 0 | 0 |  |
| SLC4A1 | 1 | 3 | -3.399 | 0.01 | 0 |  |
| AC108676.1 | 2 | 6 | -3.419 | 0 | 0 |  |
| NDST4 | 1 | 10 | -3.43 | 0 | 0 |  |
| HBA2 | 117 | 280 | -3.288 | 0 | 0 |  |
| LRRTM1 | 1 | 5 | -3.296 | 0.01 | 0 |  |
| ROS1 | 4 | 9 | -3.329 | 0 | 0 |  |
| CACNA1B | 2 | 3 | -3.331 | 0 | 0 |  |
| ADCYAP1R1 | 1 | 5 | -3.353 | 0 | 0 |  |
| GDF3 | 1 | 2 | -3.255 | 0 | 0 |  |
| ZG16 | 4 | 5 | -3.261 | 0.03 | 0.001 |  |
| OMD | 6 | 13 | -3.268 | 0 | 0 |  |
| RPE65 | 0 | 4 | -3.27 | 0 | 0 |  |
| NKX6-2 | 5 | 16 | -3.286 | 0.03 | 0.002 |  |
| SCARA5 | 17 | 150 | -3.229 | 0 | 0 |  |
| C6orf58 | 21 | 22 | -3.233 | 0.03 | 0.002 |  |
| ABCC13 | 1 | 5 | -3.235 | 0.01 | 0 |  |
| PCDHB6 | 4 | 7 | -3.252 | 0 | 0 |  |
| MYOZ3 | 7 | 17 | -3.253 | 0 | 0 |  |
| C12orf56 | 0 | 5 | -3.173 | 0.02 | 0.001 |  |
| DCX | 2 | 9 | -3.178 | 0 | 0 |  |
| CYP2B7P | 18 | 47 | -3.191 | 0 | 0 |  |
| CBLN2 | 1 | 4 | -3.212 | 0.01 | 0 |  |
| GALNT13 | 2 | 9 | -3.217 | 0.01 | 0 |  |
| RP11-587P21.2 | 2 | 7 | -3.122 | 0.02 | 0 |  |
| MS4A15 | 0 | 3 | -3.125 | 0.01 | 0 |  |
| MEOX2 | 8 | 25 | -3.129 | 0 | 0 |  |
| PNMA3 | 6 | 6 | -3.142 | 0 | 0 |  |
| AGBL4 | 1 | 3 | -3.163 | 0.01 | 0 |  |
| HS6ST2 | 5 | 14 | -3.082 | 0 | 0 |  |
| SLC13A2 | 1 | 58 | -3.092 | 0.01 | 0 |  |
| NEFM | 1 | 5 | -3.107 | 0 | 0 |  |
| GJB6 | 1 | 4 | -3.108 | 0 | 0 |  |
| EGF | 2 | 5 | -3.121 | 0 | 0 |  |
| PCSK1 | 12 | 62 | -3.05 | 0 | 0 |  |
| FOLR3 | 2 | 7 | -3.056 | 0 | 0 |  |
| NMUR2 | 20 | 55 | -3.057 | 0 | 0 |  |
| THSD7B | 6 | 12 | -3.069 | 0 | 0 |  |
| HS3ST5 | 2 | 5 | -3.081 | 0 | 0 |  |
| KCNB2 | 1 | 3 | -3.002 | 0.01 | 0 |  |
| CHGB | 14 | 108 | -3.015 | 0.01 | 0 |  |
| LHFPL3-AS2 | 4 | 44 | -3.022 | 0 | 0 |  |
| PCDHA6 | 1 | 4 | -3.025 | 0.01 | 0 |  |
| CHRDL1 | 14 | 19 | -3.026 | 0.03 | 0.001 |  |
| LINC00942 | 3 | 5 | -2.953 | 0.01 | 0 |  |
| SALL4 | 15 | 40 | -2.97 | 0.01 | 0 |  |
| ZNF676 | 3 | 3 | -2.99 | 0.01 | 0 |  |
| PCDHAC1 | 1 | 3 | -2.995 | 0.01 | 0 |  |
| MUC2 | 10 | 93 | -3 | 0.04 | 0.002 |  |
| FLG | 3 | 7 | -2.936 | 0 | 0 |  |
| SLC6A19 | 11 | 58 | -2.938 | 0.02 | 0.001 |  |
| ERICH3 | 3 | 7 | -2.943 | 0.01 | 0 |  |
| SNX31 | 1 | 6 | -2.947 | 0 | 0 |  |
| ATP13A5 | 3 | 4 | -2.952 | 0.03 | 0.001 |  |
| MUC12 | 21 | 43 | -2.903 | 0 | 0 |  |
| RP4-565E6.1 | 1 | 7 | -2.913 | 0 | 0 |  |
| SLC15A1 | 12 | 80 | -2.914 | 0 | 0 |  |
| PKP1 | 5 | 12 | -2.915 | 0.01 | 0 |  |
| TBX10 | 1 | 12 | -2.925 | 0 | 0 |  |
| PIRT | 1 | 3 | -2.882 | 0.02 | 0.001 |  |
| CYP1A1 | 1 | 3 | -2.886 | 0.02 | 0.001 |  |
| ACTN3 | 2 | 2 | -2.897 | 0.04 | 0.002 |  |
| PTPRG-AS1 | 3 | 5 | -2.9 | 0 | 0 |  |
| TACR1 | 4 | 16 | -2.902 | 0 | 0 |  |
| CTA-313A17.2 | 0 | 2 | -2.858 | 0.01 | 0 |  |
| UNC13C | 0 | 4 | -2.86 | 0.01 | 0 |  |
| AQP10 | 2 | 6 | -2.862 | 0.02 | 0.001 |  |
| DAB1 | 18 | 73 | -2.864 | 0 | 0 |  |
| PPP1R14C | 8 | 36 | -2.871 | 0 | 0 |  |
| HAVCR1 | 5 | 18 | -2.843 | 0 | 0 |  |
| PCDHGB1 | 3 | 4 | -2.847 | 0.01 | 0 |  |
| CCDC144CP | 5 | 3 | -2.848 | 0.02 | 0.001 |  |
| PNLIPRP2 | 1 | 8 | -2.85 | 0.02 | 0.001 |  |
| MUC20P1 | 21 | 92 | -2.858 | 0 | 0 |  |
| MAEL | 1 | 2 | -2.826 | 0.04 | 0.002 |  |
| TMEM132C | 2 | 16 | -2.829 | 0 | 0 |  |
| DEUP1 | 1 | 2 | -2.832 | 0.01 | 0 |  |
| DCHS2 | 18 | 80 | -2.835 | 0.01 | 0 |  |
| PRSS33 | 4 | 12 | -2.84 | 0 | 0 |  |
| HAP1 | 7 | 24 | -2.805 | 0 | 0 |  |
| TRIM58 | 2 | 10 | -2.809 | 0 | 0 |  |
| ZNF492 | 2 | 3 | -2.811 | 0.01 | 0 |  |
| MMP3 | 437 | 760 | -2.816 | 0.02 | 0.001 |  |
| SPIN2A | 2 | 4 | -2.826 | 0.01 | 0 |  |
| CLEC3B | 65 | 180 | -2.784 | 0 | 0 |  |
| ZNF208 | 8 | 18 | -2.786 | 0 | 0 |  |
| HMGCLL1 | 3 | 17 | -2.797 | 0 | 0 |  |
| COL6A5 | 6 | 13 | -2.798 | 0.01 | 0 |  |
| FBLN1 | 317 | 1,474 | -2.799 | 0 | 0 |  |
| MYO3A | 3 | 5 | -2.767 | 0.02 | 0.001 |  |
| CR2 | 12 | 40 | -2.769 | 0 | 0 |  |
| DNM1P51 | 1 | 2 | -2.769 | 0.01 | 0 |  |
| MDS2 | 1 | 3 | -2.77 | 0.01 | 0 |  |
| TBX18 | 5 | 15 | -2.771 | 0.01 | 0 |  |
| HBA1 | 39 | 83 | -2.746 | 0.01 | 0 |  |
| AC104534.2 | 2 | 10 | -2.755 | 0 | 0 |  |
| HSP90AB2P | 1 | 1 | -2.758 | 0.04 | 0.002 |  |
| SLC5A5 | 19 | 177 | -2.76 | 0.03 | 0.001 |  |
| RP13-870H17.3 | 19 | 14 | -2.767 | 0.03 | 0.002 |  |
| SCN3A | 5 | 29 | -2.732 | 0 | 0 |  |
| CEL | 14 | 18 | -2.74 | 0 | 0 |  |
| MYH13 | 3 | 9 | -2.741 | 0 | 0 |  |
| XG | 5 | 24 | -2.741 | 0.02 | 0.001 |  |
| FAM135B | 2 | 24 | -2.743 | 0.01 | 0 |  |
| FMO6P | 3 | 3 | -2.693 | 0.04 | 0.003 |  |
| PLEKHD1 | 5 | 7 | -2.694 | 0.01 | 0 |  |
| GLYATL2 | 2 | 5 | -2.697 | 0.01 | 0 |  |
| FEV | 1 | 3 | -2.708 | 0.04 | 0.002 |  |
| AC016735.2 | 0 | 3 | -2.72 | 0.01 | 0 |  |
| LINC01140 | 12 | 15 | -2.678 | 0 | 0 |  |
| RP11-596D21.1 | 2 | 3 | -2.684 | 0.02 | 0.001 |  |
| RP11-355F16.1 | 2 | 10 | -2.69 | 0 | 0 |  |
| RP11-500B12.1 | 2 | 5 | -2.69 | 0.02 | 0.001 |  |
| KCNT1 | 3 | 8 | -2.692 | 0.01 | 0 |  |
| RP11-175K6.1 | 5 | 11 | -2.664 | 0 | 0 |  |
| AKR1B15 | 2 | 8 | -2.666 | 0 | 0 |  |
| TMC2 | 1 | 3 | -2.668 | 0.02 | 0.001 |  |
| COL9A1 | 34 | 147 | -2.672 | 0 | 0 |  |
| HBB | 987 | 1,112 | -2.677 | 0.02 | 0.001 |  |
| AC020951.1 | 1 | 2 | -2.629 | 0.01 | 0 |  |
| IGKV1D-27 | 10 | 32 | -2.632 | 0.02 | 0.001 |  |
| AVPR2 | 10 | 28 | -2.638 | 0 | 0 |  |
| EBF2 | 5 | 24 | -2.653 | 0.01 | 0 |  |
| SEZ6L | 3 | 7 | -2.663 | 0.01 | 0 |  |
| GRIN2A | 5 | 35 | -2.615 | 0 | 0 |  |
| HAS1 | 7 | 12 | -2.617 | 0.01 | 0 |  |
| VEGFD | 3 | 12 | -2.62 | 0 | 0 |  |
| LINC00473 | 1 | 4 | -2.626 | 0.01 | 0 |  |
| PRELP | 37 | 123 | -2.626 | 0.01 | 0 |  |
| TINAG | 7 | 52 | -2.597 | 0.02 | 0.001 |  |
| PQLC2L | 10 | 69 | -2.601 | 0 | 0 |  |
| ST8SIA2 | 2 | 4 | -2.604 | 0.01 | 0 |  |
| RP11-536O18.1 | 3 | 4 | -2.605 | 0.02 | 0.001 |  |
| PXDNL | 4 | 11 | -2.606 | 0 | 0 |  |
| NELL1 | 5 | 27 | -2.588 | 0.01 | 0 |  |
| GABRE | 32 | 81 | -2.592 | 0 | 0 |  |
| HRASLS | 4 | 8 | -2.593 | 0 | 0 |  |
| CACNA1C-AS2 | 1 | 2 | -2.593 | 0.02 | 0.001 |  |
| ASCL1 | 1 | 12 | -2.595 | 0 | 0 |  |
| CLCA4 | 1 | 3 | -2.576 | 0.03 | 0.001 |  |
| PLPPR4 | 26 | 50 | -2.576 | 0 | 0 |  |
| RXRG | 3 | 6 | -2.579 | 0.01 | 0 |  |
| IGSF10 | 7 | 25 | -2.58 | 0 | 0 |  |
| MFAP5 | 23 | 168 | -2.583 | 0.02 | 0.001 |  |
| VIPR2 | 8 | 25 | -2.573 | 0 | 0 |  |
| SPAG17 | 2 | 15 | -2.574 | 0.03 | 0.001 |  |
| OXGR1 | 1 | 4 | -2.574 | 0.01 | 0 |  |
| IGHV7-56 | 1 | 3 | -2.574 | 0.05 | 0.003 |  |
| PCDHA4 | 3 | 9 | -2.575 | 0.01 | 0 |  |
| CACNA1I | 7 | 21 | -2.561 | 0 | 0 |  |
| FOXN1 | 1 | 3 | -2.562 | 0.02 | 0.001 |  |
| AIRE | 3 | 8 | -2.562 | 0 | 0 |  |
| GPR179 | 1 | 4 | -2.566 | 0.02 | 0.001 |  |
| ANO3 | 3 | 8 | -2.57 | 0.02 | 0.001 |  |
| SNORD3A | 5 | 7 | -2.531 | 0.04 | 0.002 |  |
| KLHL41 | 2 | 8 | -2.542 | 0.01 | 0 |  |
| TNR | 3 | 12 | -2.543 | 0 | 0 |  |
| CSMD2 | 12 | 46 | -2.545 | 0 | 0 |  |
| TCEAL5 | 2 | 5 | -2.545 | 0 | 0 |  |
| NUTM2E | 1 | 3 | -2.513 | 0.04 | 0.002 |  |
| FAR2P2 | 3 | 12 | -2.522 | 0.01 | 0 |  |
| FAM189A1 | 4 | 9 | -2.526 | 0.01 | 0 |  |
| CXCL14 | 509 | 2,710 | -2.528 | 0 | 0 |  |
| MIR7-3HG | 0 | 2 | -2.53 | 0.02 | 0.001 |  |
| HFM1 | 2 | 5 | -2.498 | 0.01 | 0 |  |
| BMS1P22 | 1 | 5 | -2.498 | 0.02 | 0.001 |  |
| CNR1 | 7 | 47 | -2.5 | 0 | 0 |  |
| WNT11 | 11 | 8 | -2.502 | 0.04 | 0.002 |  |
| AMPD1 | 10 | 38 | -2.505 | 0.02 | 0.001 |  |
| TCL6 | 4 | 7 | -2.478 | 0.01 | 0 |  |
| COL21A1 | 22 | 56 | -2.48 | 0 | 0 |  |
| OR2A1 | 1 | 3 | -2.491 | 0.03 | 0.002 |  |
| XXbac-BPG55C20.7 | 2 | 3 | -2.493 | 0.04 | 0.002 |  |
| HBD | 4 | 6 | -2.496 | 0.02 | 0.001 |  |
| IGLC7 | 38 | 121 | -2.468 | 0.04 | 0.002 |  |
| ERVFRD-1 | 1 | 3 | -2.469 | 0.02 | 0.001 |  |
| CD300E | 169 | 52 | -2.469 | 0.02 | 0.001 |  |
| SLC18A1 | 5 | 9 | -2.471 | 0.03 | 0.002 |  |
| LINC01152 | 1 | 2 | -2.478 | 0.04 | 0.002 |  |
| FZD10-AS1 | 6 | 36 | -2.447 | 0 | 0 |  |
| ANPEP | 773 | 4,374 | -2.447 | 0.01 | 0 |  |
| PCDHA10 | 3 | 13 | -2.452 | 0.01 | 0 |  |
| RP4-625H18.2 | 5 | 16 | -2.457 | 0.01 | 0 |  |
| EDN3 | 5 | 61 | -2.463 | 0.03 | 0.002 |  |
| GOLGA8VP | 1 | 3 | -2.422 | 0.02 | 0.001 |  |
| SSTR5-AS1 | 3 | 17 | -2.427 | 0.03 | 0.002 |  |
| MTND4LP30 | 3 | 9 | -2.433 | 0.03 | 0.001 |  |
| A4GNT | 25 | 229 | -2.434 | 0.04 | 0.002 |  |
| HORMAD1 | 4 | 5 | -2.443 | 0.01 | 0 |  |
| SCG2 | 19 | 100 | -2.401 | 0 | 0 |  |
| GREB1L | 6 | 58 | -2.401 | 0.02 | 0.001 |  |
| SLIT3 | 126 | 315 | -2.401 | 0 | 0 |  |
| PP14571 | 1 | 3 | -2.41 | 0.02 | 0.001 |  |
| FRG1EP | 1 | 3 | -2.422 | 0.03 | 0.001 |  |
| PTH2R | 2 | 10 | -2.386 | 0.01 | 0 |  |
| SEMA3E | 26 | 79 | -2.386 | 0.01 | 0 |  |
| FBN2 | 64 | 164 | -2.395 | 0 | 0 |  |
| MUC3A | 257 | 992 | -2.397 | 0 | 0 |  |
| HMGA1P5 | 0 | 3 | -2.4 | 0.02 | 0.001 |  |
| RFX6 | 1 | 48 | -2.373 | 0.05 | 0.003 |  |
| RP11-13K12.1 | 2 | 2 | -2.374 | 0.04 | 0.003 |  |
| FZD10 | 10 | 32 | -2.375 | 0 | 0 |  |
| ACE2 | 111 | 463 | -2.381 | 0.01 | 0 |  |
| SDK2 | 14 | 50 | -2.385 | 0 | 0 |  |
| IL11 | 46 | 47 | -2.365 | 0.05 | 0.003 |  |
| ZNF726 | 11 | 21 | -2.369 | 0.01 | 0 |  |
| ZNF727 | 1 | 6 | -2.37 | 0.01 | 0 |  |
| UCN2 | 6 | 6 | -2.371 | 0.04 | 0.002 |  |
| FLRT2 | 67 | 186 | -2.372 | 0 | 0 |  |
| ADAMTS18 | 7 | 12 | -2.35 | 0.01 | 0 |  |
| NRG1 | 51 | 281 | -2.351 | 0 | 0 |  |
| HIST3H2BB | 4 | 6 | -2.357 | 0.01 | 0 |  |
| COL7A1 | 288 | 594 | -2.358 | 0 | 0 |  |
| AKNAD1 | 1 | 4 | -2.363 | 0.02 | 0.001 |  |
| CHRNB2 | 3 | 12 | -2.342 | 0.01 | 0 |  |
| CYP2W1 | 22 | 28 | -2.343 | 0.01 | 0 |  |
| NEB | 34 | 122 | -2.343 | 0 | 0 |  |
| LCNL1 | 2 | 2 | -2.343 | 0.03 | 0.001 |  |
| RP11-170L3.7 | 1 | 5 | -2.343 | 0.03 | 0.002 |  |
| ZNF536 | 3 | 17 | -2.329 | 0.02 | 0.001 |  |
| DLGAP3 | 5 | 12 | -2.331 | 0 | 0 |  |
| WISP3 | 4 | 19 | -2.335 | 0.04 | 0.002 |  |
| RP11-242J7.1 | 1 | 2 | -2.338 | 0.04 | 0.003 |  |
| ARMC4 | 1 | 4 | -2.339 | 0.03 | 0.001 |  |
| ACAN | 47 | 97 | -2.324 | 0.02 | 0.001 |  |
| HIST2H2BA | 2 | 5 | -2.324 | 0.02 | 0.001 |  |
| RP11-157G21.2 | 2 | 3 | -2.325 | 0.02 | 0 |  |
| LINC00870 | 4 | 6 | -2.326 | 0.02 | 0.001 |  |
| UNC13A | 22 | 44 | -2.326 | 0 | 0 |  |
| SCN2B | 1 | 4 | -2.312 | 0.05 | 0.003 |  |
| FREM1 | 15 | 52 | -2.313 | 0.02 | 0 |  |
| HIST1H2BJ | 49 | 45 | -2.314 | 0.01 | 0 |  |
| LPAR4 | 2 | 9 | -2.318 | 0.01 | 0 |  |
| GUCY2C | 46 | 413 | -2.319 | 0.01 | 0 |  |
| MIR497HG | 5 | 10 | -2.29 | 0 | 0 |  |
| LINC00605 | 1 | 3 | -2.295 | 0.05 | 0.003 |  |
| TLL1 | 12 | 68 | -2.302 | 0 | 0 |  |
| HHIP | 38 | 249 | -2.308 | 0 | 0 |  |
| MUSK | 2 | 10 | -2.309 | 0.01 | 0 |  |
| CCDC178 | 3 | 9 | -2.279 | 0.02 | 0.001 |  |
| DES | 464 | 972 | -2.283 | 0.02 | 0.001 |  |
| TRHDE-AS1 | 1 | 8 | -2.283 | 0.02 | 0.001 |  |
| CACNA1G | 5 | 9 | -2.283 | 0 | 0 |  |
| PAPPA | 54 | 117 | -2.285 | 0 | 0 |  |
| TWIST2 | 13 | 27 | -2.27 | 0.01 | 0 |  |
| SLC7A4 | 4 | 38 | -2.273 | 0 | 0 |  |
| NLRP7 | 7 | 13 | -2.273 | 0.01 | 0 |  |
| UCHL1 | 39 | 94 | -2.274 | 0.02 | 0 |  |
| CYP27C1 | 2 | 7 | -2.279 | 0.03 | 0.001 |  |
| TMEM236 | 12 | 32 | -2.247 | 0.01 | 0 |  |
| RP11-384P7.7 | 2 | 3 | -2.25 | 0.03 | 0.001 |  |
| SCN3B | 6 | 18 | -2.262 | 0 | 0 |  |
| SLC17A7 | 6 | 14 | -2.263 | 0.01 | 0 |  |
| CADPS | 13 | 67 | -2.27 | 0 | 0 |  |
| RSPH14 | 3 | 6 | -2.22 | 0.02 | 0.001 |  |
| GPR142 | 1 | 5 | -2.223 | 0.01 | 0 |  |
| UNC93A | 3 | 16 | -2.227 | 0.05 | 0.003 |  |
| GABBR2 | 2 | 6 | -2.227 | 0.03 | 0.001 |  |
| RYR1 | 39 | 50 | -2.236 | 0.01 | 0 |  |
| DAZL | 3 | 7 | -2.211 | 0.04 | 0.002 |  |
| PLXNA4 | 14 | 32 | -2.215 | 0 | 0 |  |
| NDNF | 18 | 99 | -2.215 | 0 | 0 |  |
| AMER3 | 1 | 7 | -2.216 | 0.04 | 0.002 |  |
| ACTC1 | 4 | 16 | -2.219 | 0.03 | 0.002 |  |
| COL24A1 | 13 | 33 | -2.197 | 0 | 0 |  |
| LINC00840 | 2 | 5 | -2.198 | 0.02 | 0.001 |  |
| AP3B2 | 5 | 11 | -2.199 | 0.02 | 0.001 |  |
| TRHDE | 5 | 45 | -2.201 | 0.01 | 0 |  |
| ADRA2A | 140 | 420 | -2.203 | 0 | 0 |  |
| KCNJ6 | 4 | 12 | -2.176 | 0.01 | 0 |  |
| P2RX6 | 4 | 6 | -2.185 | 0.01 | 0 |  |
| RP11-736N17.10 | 2 | 4 | -2.19 | 0.02 | 0.001 |  |
| COL14A1 | 355 | 852 | -2.191 | 0 | 0 |  |
| SLC14A2 | 2 | 5 | -2.195 | 0.03 | 0.002 |  |
| RNF126P1 | 1 | 3 | -2.168 | 0.04 | 0.002 |  |
| ABI3BP | 109 | 235 | -2.172 | 0 | 0 |  |
| ROBO2 | 20 | 102 | -2.173 | 0 | 0 |  |
| RP11-286H15.1 | 2 | 4 | -2.174 | 0.02 | 0.001 |  |
| KIF26A | 66 | 187 | -2.175 | 0 | 0 |  |
| DCN | 3,398 | 7,841 | -2.153 | 0 | 0 |  |
| RP11-678G14.3 | 2 | 4 | -2.157 | 0.05 | 0.003 |  |
| ZNF730 | 1 | 3 | -2.159 | 0.03 | 0.001 |  |
| SVEP1 | 118 | 335 | -2.162 | 0 | 0 |  |
| ZNF732 | 2 | 3 | -2.163 | 0.02 | 0.001 |  |
| HSF5 | 1 | 2 | -2.148 | 0.04 | 0.003 |  |
| KCNH6 | 3 | 9 | -2.149 | 0.01 | 0 |  |
| MLC1 | 7 | 16 | -2.151 | 0 | 0 |  |
| RP11-109J4.1 | 0 | 5 | -2.151 | 0.05 | 0.003 |  |
| FCGBP | 562 | 2,564 | -2.151 | 0.03 | 0.002 |  |
| RP11-566K19.6 | 2 | 4 | -2.136 | 0.03 | 0.001 |  |
| MEGF10 | 3 | 7 | -2.139 | 0.02 | 0.001 |  |
| SCARA3 | 96 | 316 | -2.141 | 0 | 0 |  |
| LRRC18 | 1 | 4 | -2.141 | 0.04 | 0.002 |  |
| PRAP1 | 5 | 11 | -2.147 | 0.02 | 0.001 |  |
| ENTPD3 | 16 | 74 | -2.12 | 0.02 | 0.001 |  |
| CELF3 | 2 | 20 | -2.12 | 0.03 | 0.001 |  |
| GALNT15 | 39 | 82 | -2.122 | 0.01 | 0 |  |
| GCK | 4 | 13 | -2.125 | 0 | 0 |  |
| LRRC37A6P | 5 | 22 | -2.133 | 0 | 0 |  |
| ZNF157 | 0 | 2 | -2.11 | 0.02 | 0.001 |  |
| NEFH | 17 | 21 | -2.111 | 0.01 | 0 |  |
| LAMA1 | 55 | 67 | -2.111 | 0.04 | 0.002 |  |
| BTBD7P1 | 1 | 3 | -2.112 | 0.03 | 0.001 |  |
| C1GALT1C1L | 2 | 7 | -2.114 | 0.02 | 0.001 |  |
| FCER1A | 21 | 29 | -2.1 | 0.02 | 0 |  |
| ABCA13 | 7 | 33 | -2.102 | 0.01 | 0 |  |
| PCLO | 10 | 140 | -2.105 | 0.04 | 0.002 |  |
| FABP4 | 25 | 52 | -2.108 | 0.02 | 0.001 |  |
| TDRD1 | 1 | 3 | -2.109 | 0.04 | 0.002 |  |
| NANOS3 | 4 | 10 | -2.089 | 0.01 | 0 |  |
| LRRC19 | 14 | 25 | -2.09 | 0.03 | 0.001 |  |
| KCNJ3 | 23 | 65 | -2.091 | 0.03 | 0.002 |  |
| GLIS3 | 94 | 273 | -2.098 | 0 | 0 |  |
| SLC26A5 | 5 | 12 | -2.099 | 0.04 | 0.002 |  |
| NDP | 4 | 10 | -2.077 | 0.01 | 0 |  |
| TKTL1 | 3 | 7 | -2.081 | 0.05 | 0.003 |  |
| PCYT1B | 1 | 3 | -2.082 | 0.04 | 0.002 |  |
| ATP1A2 | 6 | 28 | -2.083 | 0.01 | 0 |  |
| APOD | 79 | 267 | -2.089 | 0.05 | 0.003 |  |
| ACKR1 | 143 | 372 | -2.067 | 0.02 | 0.001 |  |
| RP11-442O1.3 | 1 | 3 | -2.067 | 0.04 | 0.002 |  |
| TPTEP1 | 15 | 53 | -2.07 | 0.01 | 0 |  |
| FBN1 | 817 | 1,583 | -2.071 | 0 | 0 |  |
| AC003973.3 | 9 | 13 | -2.076 | 0.02 | 0.001 |  |
| SLC38A11 | 28 | 86 | -2.054 | 0.02 | 0.001 |  |
| ADGRL3 | 31 | 173 | -2.054 | 0 | 0 |  |
| ADAM33 | 31 | 161 | -2.056 | 0.01 | 0 |  |
| KCNS1 | 3 | 11 | -2.062 | 0.04 | 0.002 |  |
| SLCO1C1 | 6 | 13 | -2.065 | 0 | 0 |  |
| CCDC80 | 310 | 679 | -2.048 | 0.04 | 0.002 |  |
| PRL | 0 | 2 | -2.049 | 0.03 | 0.001 |  |
| CHRM4 | 2 | 8 | -2.05 | 0.02 | 0.001 |  |
| IL13RA2 | 23 | 46 | -2.05 | 0.04 | 0.003 |  |
| CRHR2 | 1 | 7 | -2.051 | 0.01 | 0 |  |
| KRT8P36 | 2 | 5 | -2.038 | 0.03 | 0.002 |  |
| CCDC144B | 9 | 46 | -2.04 | 0.03 | 0.001 |  |
| CKMT2 | 20 | 37 | -2.045 | 0.02 | 0.001 |  |
| NRCAM | 60 | 130 | -2.047 | 0.01 | 0 |  |
| NPR3 | 44 | 48 | -2.048 | 0.04 | 0.002 |  |
| ABCG2 | 90 | 171 | -2.028 | 0 | 0 |  |
| MYO7B | 34 | 461 | -2.032 | 0.03 | 0.002 |  |
| DPT | 81 | 175 | -2.033 | 0.04 | 0.002 |  |
| AMPH | 24 | 70 | -2.034 | 0 | 0 |  |
| PLAC9 | 57 | 88 | -2.038 | 0.01 | 0 |  |
| L1CAM | 28 | 79 | -2.015 | 0.01 | 0 |  |
| MUC20 | 71 | 288 | -2.019 | 0 | 0 |  |
| SPATA3-AS1 | 3 | 6 | -2.021 | 0.01 | 0 |  |
| CARNS1 | 15 | 32 | -2.023 | 0 | 0 |  |
| TMEM119 | 223 | 462 | -2.024 | 0 | 0 |  |
| TMEM132E | 7 | 15 | -2.006 | 0.01 | 0 |  |
| MAMDC2 | 20 | 93 | -2.008 | 0.02 | 0 |  |
| PRSS51 | 3 | 8 | -2.008 | 0.03 | 0.001 |  |
| C1QTNF7 | 39 | 93 | -2.011 | 0 | 0 |  |
| RGS6 | 8 | 19 | -2.011 | 0.01 | 0 |  |
| DDIT4L | 9 | 14 | -1.987 | 0.03 | 0.002 |  |
| RP11-482G13.1 | 4 | 7 | -1.988 | 0.04 | 0.003 |  |
| OBSCN | 245 | 406 | -1.999 | 0 | 0 |  |
| GJA3 | 2 | 6 | -2 | 0.05 | 0.003 |  |
| PKD1L1 | 18 | 51 | -2.002 | 0 | 0 |  |
| DLGAP1 | 10 | 11 | -1.978 | 0.05 | 0.003 |  |
| AFDN-AS1 | 15 | 24 | -1.981 | 0.01 | 0 |  |
| ZFPM2-AS1 | 7 | 29 | -1.985 | 0.01 | 0 |  |
| COL12A1 | 1,472 | 3,357 | -1.987 | 0 | 0 |  |
| TINCR | 4 | 6 | -1.987 | 0.04 | 0.002 |  |
| TFF3 | 83 | 889 | -1.97 | 0.02 | 0.001 |  |
| SPIB | 23 | 59 | -1.971 | 0.01 | 0 |  |
| AC005301.9 | 6 | 14 | -1.973 | 0.04 | 0.002 |  |
| PAPPA2 | 6 | 40 | -1.975 | 0.03 | 0.001 |  |
| ADH1C | 2,482 | 6,547 | -1.975 | 0.05 | 0.003 |  |
| FAM132A | 14 | 61 | -1.95 | 0 | 0 |  |
| TMEM151B | 1 | 3 | -1.953 | 0.03 | 0.002 |  |
| RP11-362F19.1 | 5 | 11 | -1.954 | 0.05 | 0.003 |  |
| CTD-2536I1.2 | 1 | 6 | -1.956 | 0.02 | 0.001 |  |
| TIMP4 | 9 | 34 | -1.97 | 0 | 0 |  |
| SLC35F1 | 5 | 14 | -1.938 | 0 | 0 |  |
| MMRN1 | 88 | 256 | -1.939 | 0.01 | 0 |  |
| FOXS1 | 34 | 45 | -1.949 | 0.02 | 0.001 |  |
| LRRN1 | 18 | 65 | -1.949 | 0.02 | 0.001 |  |
| PCDHA3 | 2 | 7 | -1.95 | 0.05 | 0.003 |  |
| MAB21L3 | 3 | 8 | -1.928 | 0.04 | 0.003 |  |
| SHROOM4 | 120 | 265 | -1.929 | 0 | 0 |  |
| PCDHB5 | 5 | 25 | -1.931 | 0.02 | 0.001 |  |
| RP11-327P2.7 | 2 | 6 | -1.934 | 0.04 | 0.002 |  |
| ABCA8 | 140 | 477 | -1.938 | 0.02 | 0.001 |  |
| RUNDC3A | 11 | 24 | -1.917 | 0.01 | 0 |  |
| WASIR2 | 2 | 6 | -1.922 | 0.03 | 0.001 |  |
| LINCR-0001 | 2 | 4 | -1.922 | 0.03 | 0.002 |  |
| ELN | 282 | 767 | -1.923 | 0 | 0 |  |
| BEST4 | 7 | 13 | -1.923 | 0.02 | 0.001 |  |
| HOXD1 | 3 | 8 | -1.906 | 0.01 | 0 |  |
| KCNK10 | 22 | 92 | -1.911 | 0.02 | 0.001 |  |
| ELANE | 3 | 5 | -1.913 | 0.04 | 0.002 |  |
| LUZP2 | 3 | 8 | -1.915 | 0.03 | 0.001 |  |
| NLRP6 | 15 | 22 | -1.917 | 0.05 | 0.003 |  |
| PCDHB11 | 8 | 14 | -1.886 | 0.01 | 0 |  |
| IQSEC3 | 11 | 20 | -1.886 | 0 | 0 |  |
| PRKG2 | 9 | 37 | -1.895 | 0.01 | 0 |  |
| GPR143 | 10 | 9 | -1.899 | 0.03 | 0.001 |  |
| WNT6 | 4 | 8 | -1.9 | 0.02 | 0 |  |
| RP11-182J1.14 | 4 | 5 | -1.878 | 0.04 | 0.002 |  |
| SLC5A1 | 170 | 771 | -1.882 | 0.03 | 0.001 |  |
| ARHGAP28 | 39 | 191 | -1.883 | 0 | 0 |  |
| RHOXF1-AS1 | 8 | 22 | -1.883 | 0.03 | 0.001 |  |
| WNK2 | 87 | 336 | -1.884 | 0.02 | 0.001 |  |
| ZBTB16 | 13 | 31 | -1.867 | 0.01 | 0 |  |
| CTNND2 | 13 | 22 | -1.872 | 0.05 | 0.003 |  |
| SYT3 | 2 | 5 | -1.872 | 0.03 | 0.001 |  |
| AC018647.3 | 3 | 5 | -1.873 | 0.04 | 0.002 |  |
| RP11-175K6.2 | 6 | 9 | -1.876 | 0.03 | 0.001 |  |
| FGF2 | 58 | 105 | -1.854 | 0 | 0 |  |
| COL4A6 | 30 | 153 | -1.857 | 0.02 | 0.001 |  |
| MIR27B | 2 | 4 | -1.86 | 0.04 | 0.002 |  |
| RP11-696D21.2 | 1 | 3 | -1.864 | 0.05 | 0.003 |  |
| TRPV3 | 35 | 100 | -1.867 | 0 | 0 |  |
| ZNF812P | 3 | 6 | -1.845 | 0.04 | 0.003 |  |
| ELAVL2 | 2 | 4 | -1.845 | 0.02 | 0.001 |  |
| FAT4 | 123 | 522 | -1.849 | 0 | 0 |  |
| SLC39A5 | 87 | 546 | -1.853 | 0.04 | 0.002 |  |
| SPON1 | 182 | 715 | -1.854 | 0.01 | 0 |  |
| GPER1 | 33 | 52 | -1.84 | 0 | 0 |  |
| MMP2 | 1,711 | 4,108 | -1.841 | 0 | 0 |  |
| TENM1 | 8 | 20 | -1.842 | 0.02 | 0.001 |  |
| BNC2 | 42 | 123 | -1.842 | 0 | 0 |  |
| PALM2-AKAP2 | 2 | 4 | -1.844 | 0.05 | 0.003 |  |
| MYO1A | 235 | 755 | -1.836 | 0.03 | 0.001 |  |
| TEKT5 | 5 | 12 | -1.838 | 0.04 | 0.002 |  |
| SDK1 | 74 | 237 | -1.839 | 0 | 0 |  |
| EFEMP1 | 540 | 938 | -1.839 | 0.03 | 0.001 |  |
| CHRM3 | 22 | 174 | -1.839 | 0.02 | 0.001 |  |
| DPY19L2 | 12 | 32 | -1.833 | 0.02 | 0.001 |  |
| PRRG3 | 5 | 11 | -1.834 | 0.03 | 0.002 |  |
| ADRA1D | 3 | 6 | -1.834 | 0.05 | 0.003 |  |
| VCAN | 1,082 | 3,262 | -1.836 | 0 | 0 |  |
| CABYR | 10 | 27 | -1.836 | 0.01 | 0 |  |
| FBLN2 | 246 | 505 | -1.823 | 0.02 | 0.001 |  |
| ALS2CR12 | 5 | 7 | -1.828 | 0.02 | 0.001 |  |
| FGF18 | 10 | 12 | -1.83 | 0.04 | 0.002 |  |
| PTGFR | 30 | 64 | -1.83 | 0.02 | 0.001 |  |
| AKR1C2 | 250 | 1,274 | -1.832 | 0.03 | 0.001 |  |
| MPV17L | 46 | 122 | -1.809 | 0.02 | 0.001 |  |
| FREM2 | 32 | 76 | -1.81 | 0.04 | 0.003 |  |
| AFF2 | 7 | 17 | -1.816 | 0.04 | 0.003 |  |
| ANKDD1B | 9 | 39 | -1.821 | 0 | 0 |  |
| NKX3-2 | 4 | 14 | -1.822 | 0.02 | 0.001 |  |
| MAP1A | 77 | 160 | -1.797 | 0.01 | 0 |  |
| SCUBE3 | 24 | 41 | -1.797 | 0.01 | 0 |  |
| MOV10L1 | 9 | 16 | -1.805 | 0.02 | 0.001 |  |
| SORCS2 | 31 | 70 | -1.806 | 0 | 0 |  |
| F13A1 | 306 | 674 | -1.807 | 0.02 | 0.001 |  |
| GALNT16 | 22 | 53 | -1.788 | 0.04 | 0.002 |  |
| FGF10 | 5 | 35 | -1.788 | 0.02 | 0.001 |  |
| ANK2 | 54 | 161 | -1.788 | 0.03 | 0.001 |  |
| HSPA2 | 135 | 369 | -1.789 | 0 | 0 |  |
| ZNF418 | 22 | 49 | -1.796 | 0 | 0 |  |
| ST18 | 9 | 25 | -1.772 | 0.05 | 0.003 |  |
| NLGN4X | 15 | 30 | -1.778 | 0.02 | 0.001 |  |
| TNXB | 103 | 355 | -1.779 | 0.01 | 0 |  |
| PCDHB18P | 5 | 8 | -1.779 | 0.02 | 0.001 |  |
| TRIM17 | 8 | 13 | -1.779 | 0.01 | 0 |  |
| NEGR1 | 69 | 217 | -1.755 | 0.03 | 0.001 |  |
| CNTNAP3B | 11 | 16 | -1.756 | 0.02 | 0.001 |  |
| FHL1 | 461 | 1,533 | -1.762 | 0.01 | 0 |  |
| AC013463.2 | 5 | 18 | -1.767 | 0.04 | 0.002 |  |
| AC012368.1 | 1 | 3 | -1.771 | 0.05 | 0.003 |  |
| MEOX1 | 90 | 136 | -1.745 | 0.02 | 0.001 |  |
| TMC1 | 2 | 5 | -1.746 | 0.01 | 0 |  |
| DYNC2H1 | 85 | 266 | -1.747 | 0.01 | 0 |  |
| PCDH19 | 3 | 25 | -1.748 | 0.04 | 0.003 |  |
| SLC5A9 | 18 | 47 | -1.754 | 0.05 | 0.003 |  |
| DNM1P46 | 3 | 8 | -1.725 | 0.01 | 0 |  |
| WNT5A | 454 | 517 | -1.735 | 0.02 | 0.001 |  |
| SPTB | 16 | 39 | -1.735 | 0.04 | 0.002 |  |
| NAALAD2 | 17 | 33 | -1.739 | 0.01 | 0 |  |
| RBMS1P1 | 3 | 6 | -1.74 | 0.03 | 0.001 |  |
| CNTN4 | 40 | 136 | -1.719 | 0 | 0 |  |
| HRC | 8 | 22 | -1.719 | 0 | 0 |  |
| PGR | 12 | 32 | -1.719 | 0.01 | 0 |  |
| RERG | 101 | 173 | -1.723 | 0.01 | 0 |  |
| LINC00327 | 3 | 7 | -1.723 | 0.03 | 0.001 |  |
| COLEC12 | 93 | 233 | -1.71 | 0.02 | 0.001 |  |
| PID1 | 130 | 278 | -1.711 | 0 | 0 |  |
| ARHGEF4 | 27 | 98 | -1.718 | 0 | 0 |  |
| CNTFR | 8 | 32 | -1.718 | 0.02 | 0.001 |  |
| CPAMD8 | 30 | 77 | -1.718 | 0 | 0 |  |
| NFASC | 101 | 244 | -1.698 | 0 | 0 |  |
| PCDHB3 | 7 | 18 | -1.702 | 0.01 | 0 |  |
| ZNF135 | 15 | 38 | -1.705 | 0.01 | 0 |  |
| NIPAL4 | 6 | 8 | -1.708 | 0.02 | 0.001 |  |
| ZNF385D | 26 | 51 | -1.709 | 0.03 | 0.001 |  |
| HMCN2 | 30 | 86 | -1.689 | 0.02 | 0.001 |  |
| ZNF423 | 35 | 100 | -1.69 | 0 | 0 |  |
| MYH7B | 24 | 31 | -1.695 | 0.02 | 0.001 |  |
| ADAMTS9 | 323 | 550 | -1.695 | 0 | 0 |  |
| ATRNL1 | 19 | 52 | -1.697 | 0.03 | 0.002 |  |
| GLI3 | 51 | 96 | -1.677 | 0 | 0 |  |
| SCX | 25 | 35 | -1.681 | 0.04 | 0.003 |  |
| LINC01013 | 3 | 10 | -1.682 | 0.03 | 0.001 |  |
| FAM184A | 26 | 29 | -1.682 | 0.02 | 0.001 |  |
| RBMS3 | 74 | 125 | -1.688 | 0 | 0 |  |
| TRIM9 | 9 | 28 | -1.671 | 0.01 | 0 |  |
| PHEX | 12 | 27 | -1.672 | 0 | 0 |  |
| CXXC4 | 20 | 60 | -1.673 | 0 | 0 |  |
| RHCE | 6 | 13 | -1.676 | 0.04 | 0.002 |  |
| TTBK1 | 11 | 17 | -1.676 | 0.03 | 0.001 |  |
| NTRK3 | 5 | 11 | -1.665 | 0.01 | 0 |  |
| PPP1R14D | 32 | 159 | -1.667 | 0.02 | 0.001 |  |
| EBF1 | 74 | 149 | -1.667 | 0 | 0 |  |
| OLFM1 | 104 | 258 | -1.668 | 0 | 0 |  |
| HSPG2 | 2,156 | 4,967 | -1.668 | 0 | 0 |  |
| DYNC1I1 | 15 | 47 | -1.652 | 0 | 0 |  |
| HIST1H2BG | 23 | 75 | -1.656 | 0.01 | 0 |  |
| GPIHBP1 | 13 | 21 | -1.66 | 0.04 | 0.002 |  |
| MYL4 | 9 | 15 | -1.661 | 0.04 | 0.002 |  |
| SLIT1 | 10 | 14 | -1.662 | 0.04 | 0.002 |  |
| CCDC3 | 387 | 746 | -1.159 | 0.04 | 0.002 |  |
| KLF8 | 39 | 81 | -1.16 | 0.01 | 0 |  |
| PABPC5 | 13 | 17 | -1.162 | 0.04 | 0.002 |  |
| CRMP1 | 101 | 154 | -1.165 | 0.02 | 0.001 |  |
| LDLRAD3 | 98 | 191 | -1.165 | 0.01 | 0 |  |
