# Supplementary material for: Integrated multi-omics analysis and machine learning identify hub genes and potential mechanisms of resistance to immunotherapy in gastric cancer
Source: Aging (Albany NY). 2024 Apr 22;16(8):7331–56. doi: 10.18632/aging.205760 (PMC11087130; doi:10.18632/aging.205760)
Supplement: Supplementary Table 2 [file aging-16-205760-s003.docx]

| Supplementary Table 2. 262 CAFs-related genes identified by WGCNA. | | | | |  |
| --- | --- | --- | --- | --- | --- |
|  |  |  |  |  |  |
| **Gene Symbol** | **HR** | **HR.95L** | **HR.95H** | **P value** |  |
| NHSL2 | 1.17961 | 1.0237 | 1.35925 | 0.02238 |  |
| FBXL7 | 1.27345 | 1.10011 | 1.47411 | 0.0012 |  |
| ZCCHC24 | 1.1472 | 1.02369 | 1.28562 | 0.01813 |  |
| MAGI2-AS3 | 1.3251 | 1.09261 | 1.60706 | 0.00424 |  |
| NAP1L3 | 1.27619 | 1.08422 | 1.50215 | 0.00337 |  |
| PABPC5 | 1.53488 | 1.17094 | 2.01194 | 0.00192 |  |
| BNC2 | 1.18047 | 1.03057 | 1.35216 | 0.01664 |  |
| CNRIP1 | 1.31069 | 1.10483 | 1.5549 | 0.00191 |  |
| TTC28 | 1.19398 | 1.01387 | 1.40608 | 0.03358 |  |
| DDR2 | 1.16778 | 1.04478 | 1.30526 | 0.00631 |  |
| STON1 | 1.17154 | 1.03949 | 1.32036 | 0.00947 |  |
| GLI3 | 1.21933 | 1.0435 | 1.42479 | 0.01256 |  |
| RHOJ | 1.22495 | 1.05104 | 1.42763 | 0.0094 |  |
| AKT3 | 1.24756 | 1.07991 | 1.44122 | 0.00266 |  |
| ATP8B2 | 1.19827 | 1.04321 | 1.37639 | 0.01052 |  |
| PRKD1 | 1.34745 | 1.13853 | 1.5947 | 0.00052 |  |
| VSTM4 | 1.19685 | 1.03721 | 1.38107 | 0.01389 |  |
| RECK | 1.33496 | 1.13072 | 1.5761 | 0.00065 |  |
| NPR2 | 1.22891 | 1.00189 | 1.50738 | 0.04792 |  |
| ZNF521 | 1.30632 | 1.10987 | 1.53755 | 0.00131 |  |
| PKD2 | 1.22713 | 1.04372 | 1.44276 | 0.01321 |  |
| SSC5D | 1.13246 | 1.02539 | 1.25071 | 0.0141 |  |
| EVC | 1.16317 | 1.0187 | 1.32814 | 0.0255 |  |
| EDNRA | 1.24028 | 1.0807 | 1.42342 | 0.00218 |  |
| SGCD | 1.15762 | 1.02776 | 1.30389 | 0.01591 |  |
| ARHGEF25 | 1.16057 | 1.0076 | 1.33677 | 0.03893 |  |
| MFAP4 | 1.09494 | 1.00613 | 1.19159 | 0.03558 |  |
| DPYSL3 | 1.14424 | 1.04435 | 1.25368 | 0.00384 |  |
| BOC | 1.16235 | 1.0482 | 1.28892 | 0.00434 |  |
| FSTL1 | 1.23749 | 1.07452 | 1.42518 | 0.0031 |  |
| OLFML1 | 1.29438 | 1.10266 | 1.51942 | 0.00161 |  |
| PARVA | 1.20762 | 1.02171 | 1.42735 | 0.02698 |  |
| ERG | 1.37062 | 1.13862 | 1.6499 | 0.00086 |  |
| LAMA4 | 1.24863 | 1.0707 | 1.45612 | 0.00464 |  |
| SYDE1 | 1.22921 | 1.04999 | 1.43902 | 0.01027 |  |
| CLMP | 1.13669 | 1.02649 | 1.25872 | 0.0138 |  |
| DLC1 | 1.27865 | 1.09 | 1.49994 | 0.00254 |  |
| LAMA2 | 1.2277 | 1.07964 | 1.39606 | 0.00176 |  |
| ZFPM2 | 1.28956 | 1.10393 | 1.5064 | 0.00134 |  |
| NDN | 1.18343 | 1.0488 | 1.33534 | 0.00627 |  |
| MMRN2 | 1.2864 | 1.08922 | 1.51928 | 0.00301 |  |
| PODN | 1.14575 | 1.03849 | 1.26408 | 0.00667 |  |
| ECM2 | 1.30232 | 1.11133 | 1.52612 | 0.0011 |  |
| PLXDC2 | 1.19822 | 1.05511 | 1.36074 | 0.00533 |  |
| CCDC80 | 1.12864 | 1.03591 | 1.22967 | 0.00566 |  |
| TEK | 1.26715 | 1.06552 | 1.50694 | 0.00741 |  |
| PDGFRB | 1.1962 | 1.04085 | 1.37475 | 0.0116 |  |
| CLIC4 | 1.19514 | 1.0273 | 1.39039 | 0.02095 |  |
| ROR2 | 1.1791 | 1.05451 | 1.31842 | 0.00383 |  |
| NFATC4 | 1.2066 | 1.02864 | 1.41535 | 0.02107 |  |
| TGFB1I1 | 1.16986 | 1.03482 | 1.32253 | 0.01218 |  |
| ARHGEF17 | 1.2195 | 1.04009 | 1.42984 | 0.01452 |  |
| C1S | 1.14644 | 1.00636 | 1.30603 | 0.03985 |  |
| BICC1 | 1.20979 | 1.05615 | 1.38578 | 0.00599 |  |
| ADAMTS10 | 1.21504 | 1.02149 | 1.44526 | 0.0278 |  |
| COL8A1 | 1.13856 | 1.03107 | 1.25725 | 0.01033 |  |
| EFEMP2 | 1.22517 | 1.05737 | 1.41959 | 0.00689 |  |
| TUBA1A | 1.18433 | 1.04877 | 1.33742 | 0.00638 |  |
| KCNE4 | 1.20276 | 1.05465 | 1.37166 | 0.00589 |  |
| FBLN5 | 1.2302 | 1.08486 | 1.395 | 0.00124 |  |
| PTGER3 | 1.23849 | 1.06992 | 1.43361 | 0.00417 |  |
| CDH11 | 1.22873 | 1.07183 | 1.4086 | 0.00313 |  |
| VEGFC | 1.32397 | 1.09606 | 1.59926 | 0.0036 |  |
| IL1R1 | 1.18224 | 1.01674 | 1.37468 | 0.02958 |  |
| GPR162 | 1.37727 | 1.07415 | 1.76592 | 0.0116 |  |
| FBN1 | 1.20174 | 1.06655 | 1.35406 | 0.00254 |  |
| GLI1 | 1.22436 | 1.05898 | 1.41556 | 0.00626 |  |
| LRRC32 | 1.21829 | 1.06577 | 1.39262 | 0.00381 |  |
| BEND6 | 1.53192 | 1.19539 | 1.96319 | 0.00075 |  |
| COL6A2 | 1.16012 | 1.02222 | 1.31663 | 0.02143 |  |
| SVEP1 | 1.21411 | 1.07708 | 1.36859 | 0.0015 |  |
| CYP7B1 | 1.23729 | 1.07006 | 1.43065 | 0.00405 |  |
| DCN | 1.17253 | 1.04775 | 1.31218 | 0.00556 |  |
| SLIT3 | 1.16614 | 1.04267 | 1.30423 | 0.00711 |  |
| COL14A1 | 1.13378 | 1.03255 | 1.24493 | 0.00851 |  |
| TIMP2 | 1.24187 | 1.07636 | 1.43282 | 0.00299 |  |
| HEG1 | 1.19235 | 1.02503 | 1.38697 | 0.02258 |  |
| RFTN2 | 1.44195 | 1.09246 | 1.90324 | 0.00976 |  |
| HMCN1 | 1.18689 | 1.03282 | 1.36395 | 0.01573 |  |
| IGFBP7 | 1.32648 | 1.1362 | 1.54864 | 0.00035 |  |
| ISM1 | 1.16954 | 1.04991 | 1.3028 | 0.00445 |  |
| PECAM1 | 1.24257 | 1.0533 | 1.46585 | 0.01 |  |
| TGFB3 | 1.19102 | 1.03992 | 1.36408 | 0.01155 |  |
| CRISPLD2 | 1.18303 | 1.0305 | 1.35813 | 0.01701 |  |
| HTR2A | 1.30048 | 1.05999 | 1.59553 | 0.01179 |  |
| GLT8D2 | 1.27441 | 1.09632 | 1.48144 | 0.00159 |  |
| NPR1 | 1.27362 | 1.09241 | 1.4849 | 0.00201 |  |
| PTGIR | 1.26119 | 1.02894 | 1.54587 | 0.02544 |  |
| AMPH | 1.2669 | 1.03431 | 1.55179 | 0.02226 |  |
| DACT1 | 1.17383 | 1.03064 | 1.33691 | 0.01575 |  |
| HSPA12B | 1.23105 | 1.02918 | 1.47253 | 0.02293 |  |
| CD34 | 1.3048 | 1.08805 | 1.56472 | 0.0041 |  |
| COLEC12 | 1.20873 | 1.05974 | 1.37868 | 0.00474 |  |
| ARHGEF15 | 1.25107 | 1.03533 | 1.51177 | 0.02037 |  |
| PRICKLE1 | 1.50914 | 1.24617 | 1.82762 | 2.5E-05 |  |
| DLG4 | 1.30504 | 1.04289 | 1.63307 | 0.01996 |  |
| TMEM204 | 1.24535 | 1.03146 | 1.5036 | 0.02248 |  |
| FAM43B | 1.36115 | 1.07357 | 1.72577 | 0.01089 |  |
| COL6A3 | 1.13713 | 1.00911 | 1.28141 | 0.03497 |  |
| EHD2 | 1.17294 | 1.02619 | 1.34068 | 0.01933 |  |
| TIE1 | 1.26706 | 1.06433 | 1.5084 | 0.00779 |  |
| LZTS1 | 1.20112 | 1.03303 | 1.39656 | 0.0172 |  |
| MARVELD1 | 1.24462 | 1.06796 | 1.4505 | 0.00508 |  |
| RHOQ | 1.34797 | 1.05237 | 1.7266 | 0.01807 |  |
| ELN | 1.13771 | 1.03051 | 1.25606 | 0.01061 |  |
| SPON1 | 1.12206 | 1.02991 | 1.22245 | 0.00843 |  |
| RAB31 | 1.22253 | 1.0531 | 1.41922 | 0.0083 |  |
| FKBP7 | 1.45161 | 1.1737 | 1.79533 | 0.00059 |  |
| IGFBP5 | 1.17739 | 1.05071 | 1.31934 | 0.00493 |  |
| COPZ2 | 1.23532 | 1.07544 | 1.41898 | 0.0028 |  |
| LPAR4 | 1.48763 | 1.08397 | 2.04159 | 0.01392 |  |
| ANGPTL2 | 1.16013 | 1.02934 | 1.30753 | 0.01494 |  |
| FIBIN | 1.21519 | 1.06441 | 1.38732 | 0.00393 |  |
| GNG11 | 1.40345 | 1.18092 | 1.66791 | 0.00012 |  |
| RNF144A | 1.33733 | 1.12517 | 1.58949 | 0.00097 |  |
| ENG | 1.2612 | 1.04195 | 1.52659 | 0.01723 |  |
| PRDM6 | 1.49578 | 1.17652 | 1.90168 | 0.00101 |  |
| SERPINF1 | 1.18977 | 1.06161 | 1.3334 | 0.00281 |  |
| FBLN1 | 1.13556 | 1.04258 | 1.23683 | 0.00354 |  |
| LTBP2 | 1.15565 | 1.02162 | 1.30726 | 0.02145 |  |
| SRPX | 1.16366 | 1.05125 | 1.28808 | 0.00345 |  |
| ST6GALNAC5 | 1.17084 | 1.00757 | 1.36056 | 0.03955 |  |
| NOVA2 | 1.32569 | 1.02321 | 1.71759 | 0.03288 |  |
| COL3A1 | 1.15257 | 1.03565 | 1.28267 | 0.00927 |  |
| CSDC2 | 1.1792 | 1.01642 | 1.36804 | 0.02964 |  |
| ASPN | 1.14719 | 1.04607 | 1.25809 | 0.00354 |  |
| LUM | 1.19472 | 1.06015 | 1.34637 | 0.00352 |  |
| SORCS2 | 1.22856 | 1.06108 | 1.42247 | 0.00591 |  |
| GGT5 | 1.2267 | 1.0682 | 1.40872 | 0.0038 |  |
| IGFBP4 | 1.14238 | 1.0129 | 1.28842 | 0.0301 |  |
| CDH5 | 1.2211 | 1.02624 | 1.45296 | 0.02433 |  |
| PTGFR | 1.23959 | 1.0563 | 1.45469 | 0.00852 |  |
| COL15A1 | 1.15667 | 1.01217 | 1.32181 | 0.03255 |  |
| COL8A2 | 1.14969 | 1.0113 | 1.30703 | 0.03303 |  |
| TWIST2 | 1.15507 | 1.02358 | 1.30346 | 0.01939 |  |
| THBS1 | 1.18665 | 1.06247 | 1.32534 | 0.00241 |  |
| ISLR | 1.13459 | 1.03106 | 1.24852 | 0.0097 |  |
| TNC | 1.09506 | 1.00278 | 1.19584 | 0.0432 |  |
| KIAA1755 | 1.19952 | 1.01183 | 1.42203 | 0.03613 |  |
| BCL6B | 1.24736 | 1.03401 | 1.50473 | 0.02092 |  |
| TIMP3 | 1.14175 | 1.01858 | 1.27981 | 0.02284 |  |
| SCARF2 | 1.13164 | 1.00094 | 1.2794 | 0.04827 |  |
| ITGBL1 | 1.13318 | 1.01587 | 1.26404 | 0.02493 |  |
| LRRC17 | 1.28931 | 1.09971 | 1.5116 | 0.00174 |  |
| FBLN2 | 1.10889 | 1.00593 | 1.22239 | 0.03762 |  |
| MMP2 | 1.12364 | 1.00489 | 1.25642 | 0.0408 |  |
| PRRX1 | 1.14861 | 1.0209 | 1.29229 | 0.02123 |  |
| SFRP4 | 1.08664 | 1.0166 | 1.1615 | 0.01451 |  |
| GXYLT2 | 1.18832 | 1.05729 | 1.33559 | 0.0038 |  |
| OLFML3 | 1.18012 | 1.04619 | 1.3312 | 0.00705 |  |
| FNDC1 | 1.13999 | 1.04881 | 1.2391 | 0.00207 |  |
| DOK5 | 1.28337 | 1.07083 | 1.53809 | 0.00692 |  |
| TMEM119 | 1.13323 | 1.00909 | 1.27265 | 0.03461 |  |
| CYGB | 1.18804 | 1.00319 | 1.40697 | 0.04584 |  |
| THY1 | 1.20602 | 1.04706 | 1.3891 | 0.00939 |  |
| SLC24A3 | 1.16273 | 1.01921 | 1.32646 | 0.0249 |  |
| AEBP1 | 1.15015 | 1.02766 | 1.28724 | 0.0149 |  |
| C8orf48 | 1.73644 | 1.25901 | 2.39493 | 0.00077 |  |
| BGN | 1.18692 | 1.05287 | 1.33804 | 0.00507 |  |
| GLIS2 | 1.20667 | 1.03669 | 1.40451 | 0.0153 |  |
| GFPT2 | 1.17872 | 1.04153 | 1.33399 | 0.0092 |  |
| FZD1 | 1.20158 | 1.01525 | 1.42212 | 0.03268 |  |
| SPARC | 1.25383 | 1.09505 | 1.43562 | 0.00106 |  |
| CLEC11A | 1.14658 | 1.00117 | 1.31311 | 0.04806 |  |
| SRPX2 | 1.28232 | 1.0914 | 1.50664 | 0.0025 |  |
| GJA4 | 1.20574 | 1.01393 | 1.43384 | 0.03431 |  |
| MEDAG | 1.16401 | 1.0433 | 1.29869 | 0.00655 |  |
| OLFML2B | 1.18611 | 1.05558 | 1.33279 | 0.00412 |  |
| HLX | 1.54813 | 1.13441 | 2.11275 | 0.00587 |  |
| SFRP2 | 1.08831 | 1.02497 | 1.15557 | 0.00567 |  |
| COL1A2 | 1.1493 | 1.02879 | 1.28391 | 0.01381 |  |
| PCOLCE | 1.1661 | 1.01121 | 1.34472 | 0.03458 |  |
| CMTM3 | 1.2729 | 1.08712 | 1.49043 | 0.00272 |  |
| PDLIM7 | 1.14881 | 1.00025 | 1.31942 | 0.04958 |  |
| CD248 | 1.1906 | 1.03016 | 1.37603 | 0.01816 |  |
| C11orf96 | 1.20156 | 1.06103 | 1.3607 | 0.00381 |  |
| LINC00968 | 1.70576 | 1.18472 | 2.45596 | 0.00409 |  |
| MSC | 1.18248 | 1.01032 | 1.38396 | 0.03681 |  |
| CTSK | 1.19885 | 1.03476 | 1.38895 | 0.01574 |  |
| MFGE8 | 1.25967 | 1.06285 | 1.49294 | 0.00774 |  |
| MFAP5 | 1.13043 | 1.02679 | 1.24455 | 0.01246 |  |
| NCAM2 | 1.34965 | 1.10275 | 1.65182 | 0.00363 |  |
| THBS2 | 1.10129 | 1.00968 | 1.2012 | 0.02945 |  |
| TSPAN4 | 1.21047 | 1.00151 | 1.46304 | 0.0482 |  |
| FHL3 | 1.24713 | 1.0292 | 1.51121 | 0.02421 |  |
| RAB3IL1 | 1.21923 | 1.04198 | 1.42664 | 0.0134 |  |
| MXRA8 | 1.17573 | 1.05024 | 1.31622 | 0.00494 |  |
| P4HA3 | 1.29872 | 1.08374 | 1.55635 | 0.00464 |  |
| PDGFRL | 1.26244 | 1.11994 | 1.42309 | 0.00014 |  |
| HTRA1 | 1.25324 | 1.07891 | 1.45574 | 0.00314 |  |
| VIM | 1.3516 | 1.13023 | 1.61632 | 0.00096 |  |
| GAS1 | 1.12356 | 1.02702 | 1.22917 | 0.01104 |  |
| HTRA3 | 1.19583 | 1.06123 | 1.34749 | 0.00333 |  |
| RARRES2 | 1.16507 | 1.00915 | 1.34508 | 0.03714 |  |
| FMO1 | 1.18052 | 1.01653 | 1.37096 | 0.02964 |  |
