# Supplementary material for: Integrated multi-omics analysis and machine learning identify hub genes and potential mechanisms of resistance to immunotherapy in gastric cancer
Source: Aging (Albany NY). 2024 Apr 22;16(8):7331–56. doi: 10.18632/aging.205760 (PMC11087130; doi:10.18632/aging.205760)
Supplement: Supplementary Table 3 [file aging-16-205760-s004.docx]

| Supplementary Table 3. 195 genes obtained by multiple screening methods for constructing the model. | | | |  |
| --- | --- | --- | --- | --- |
|  |  |  |  |  |
| **Gene Symbol** | **GS.Fibroblast_MCPCOUNTER** | **GS.Fibroblast_XCELL** | **GS.CAFs_EPIC** |  |
| COL1A2 | 0.890036029 | 0.522028935 | 0.959984308 |  |
| COL3A1 | 0.894610059 | 0.578843415 | 0.944831428 |  |
| COL6A2 | 0.93025248 | 0.722093066 | 0.862434471 |  |
| SPARC | 0.843358355 | 0.545131854 | 0.857889761 |  |
| MMP2 | 0.836997442 | 0.595789452 | 0.853113096 |  |
| COL6A3 | 0.842473575 | 0.595096273 | 0.844395423 |  |
| BGN | 0.821585274 | 0.625381124 | 0.82768052 |  |
| MRC2 | 0.833443828 | 0.649069181 | 0.822063402 |  |
| AEBP1 | 0.821663677 | 0.618968839 | 0.812872807 |  |
| PRRX1 | 0.799229516 | 0.607450924 | 0.809929856 |  |
| PCOLCE | 0.804019028 | 0.621085912 | 0.809388835 |  |
| CDH11 | 0.841093152 | 0.683807315 | 0.802040215 |  |
| THBS2 | 0.757431156 | 0.509704784 | 0.800058758 |  |
| GLT8D2 | 0.844072822 | 0.720959401 | 0.793295751 |  |
| CD248 | 0.767962196 | 0.508170996 | 0.787028918 |  |
| LUM | 0.787977702 | 0.641333937 | 0.780637884 |  |
| EFEMP2 | 0.874447723 | 0.761144481 | 0.77910839 |  |
| PDGFRB | 0.810667398 | 0.650863725 | 0.777011928 |  |
| CLEC11A | 0.81216533 | 0.662589378 | 0.77599823 |  |
| THY1 | 0.793352812 | 0.67960182 | 0.764080676 |  |
| SCARF2 | 0.818553194 | 0.624715379 | 0.762075443 |  |
| FNDC1 | 0.754477517 | 0.574755706 | 0.752415089 |  |
| FBN1 | 0.763797921 | 0.646568259 | 0.748944359 |  |
| EMILIN1 | 0.881457615 | 0.813540262 | 0.743074417 |  |
| CPXM1 | 0.722159726 | 0.576882486 | 0.739601239 |  |
| ISLR | 0.812969241 | 0.686277412 | 0.739341546 |  |
| FSTL1 | 0.81524014 | 0.712144587 | 0.73678611 |  |
| TIMP2 | 0.819635843 | 0.648377642 | 0.736396537 |  |
| GPR68 | 0.739089541 | 0.575453879 | 0.727943518 |  |
| GLI3 | 0.83765164 | 0.752297977 | 0.72785962 |  |
| SH3PXD2B | 0.738184315 | 0.518246832 | 0.726619298 |  |
| SYDE1 | 0.85694962 | 0.737952046 | 0.723439049 |  |
| COL8A2 | 0.77788729 | 0.638199073 | 0.717378728 |  |
| OLFML1 | 0.780805997 | 0.741255235 | 0.717189075 |  |
| FBLN2 | 0.71725292 | 0.606207574 | 0.715785032 |  |
| ANGPTL2 | 0.821435879 | 0.631877878 | 0.714797693 |  |
| FIBIN | 0.825348154 | 0.688535866 | 0.714117452 |  |
| CTSK | 0.736971998 | 0.563763418 | 0.712926544 |  |
| TGFB3 | 0.754222741 | 0.695819038 | 0.705845116 |  |
| COPZ2 | 0.839604148 | 0.761589624 | 0.705452001 |  |
| GFPT2 | 0.72757024 | 0.599021894 | 0.703867164 |  |
| AL356417.2 | 0.711815536 | 0.588362225 | 0.699612212 |  |
| COL8A1 | 0.771401696 | 0.71282189 | 0.697941555 |  |
| ZNF521 | 0.756025265 | 0.710410717 | 0.694064693 |  |
| SRPX2 | 0.711618272 | 0.517525321 | 0.693988589 |  |
| ADGRA2 | 0.756707681 | 0.665406662 | 0.692954616 |  |
| SERPINF1 | 0.756960104 | 0.755267462 | 0.690946263 |  |
| GAS1 | 0.705095954 | 0.57422405 | 0.684064415 |  |
| HTRA3 | 0.70204304 | 0.544688908 | 0.681897698 |  |
| RAB31 | 0.765864455 | 0.602697501 | 0.680411885 |  |
| TWIST2 | 0.73509937 | 0.708185427 | 0.67761758 |  |
| BICC1 | 0.726980709 | 0.683139642 | 0.677389448 |  |
| PHLDB1 | 0.747008219 | 0.645262787 | 0.670507404 |  |
| C1R | 0.812869954 | 0.822291266 | 0.669617242 |  |
| SFRP2 | 0.694183084 | 0.655191973 | 0.664405768 |  |
| VSTM4 | 0.780641408 | 0.763864852 | 0.664402549 |  |
| CRISPLD2 | 0.72488085 | 0.656808013 | 0.663822867 |  |
| HIC1 | 0.761553106 | 0.766141935 | 0.663148498 |  |
| VEGFC | 0.740783026 | 0.712425055 | 0.662584353 |  |
| TIMP3 | 0.733045629 | 0.610254417 | 0.662337047 |  |
| SYNDIG1 | 0.686945512 | 0.572089869 | 0.659814131 |  |
| CAVIN1 | 0.838848225 | 0.789041051 | 0.658604265 |  |
| PLXDC2 | 0.741456559 | 0.704414643 | 0.658181499 |  |
| SFRP4 | 0.715773461 | 0.680010422 | 0.656097404 |  |
| DCN | 0.761774713 | 0.777107113 | 0.655209908 |  |
| ARMH4 | 0.699621598 | 0.55965844 | 0.654572815 |  |
| CMTM3 | 0.691872414 | 0.523796067 | 0.653922838 |  |
| LAMA4 | 0.742379849 | 0.590009208 | 0.65262741 |  |
| RARRES2 | 0.695729327 | 0.577871542 | 0.652425442 |  |
| NLGN2 | 0.743976999 | 0.654837946 | 0.650969925 |  |
| LRRC32 | 0.721583777 | 0.693550567 | 0.650542809 |  |
| COL15A1 | 0.696470825 | 0.564945376 | 0.650157901 |  |
| SNED1 | 0.675688136 | 0.605525434 | 0.649328839 |  |
| EVC | 0.734008555 | 0.682370151 | 0.648480495 |  |
| FBXL7 | 0.779358673 | 0.771859561 | 0.644591945 |  |
| P4HA3 | 0.65434996 | 0.516777898 | 0.644480857 |  |
| MAP1A | 0.807297201 | 0.806475639 | 0.644346667 |  |
| HLX | 0.666698196 | 0.547123017 | 0.644021112 |  |
| NFATC4 | 0.756520106 | 0.694875157 | 0.643255777 |  |
| PCDHGA12 | 0.697166404 | 0.649783326 | 0.642731881 |  |
| KIRREL1 | 0.709404578 | 0.590463684 | 0.642464894 |  |
| CCDC80 | 0.753701101 | 0.853510873 | 0.641118856 |  |
| GJA5 | 0.650765374 | 0.503157158 | 0.637876888 |  |
| ECM2 | 0.711058984 | 0.726303825 | 0.635427753 |  |
| SSC5D | 0.819968468 | 0.817725107 | 0.635370672 |  |
| LRRC17 | 0.677029793 | 0.582279145 | 0.635033878 |  |
| ITGA5 | 0.74183513 | 0.530269654 | 0.634324666 |  |
| THBS1 | 0.66886517 | 0.551207614 | 0.634225737 |  |
| AC080038.1 | 0.709053511 | 0.709234204 | 0.630011041 |  |
| PRKD1 | 0.768461593 | 0.711591053 | 0.629911237 |  |
| MSC-AS1 | 0.679343139 | 0.610056395 | 0.629583447 |  |
| ST6GALNAC5 | 0.740321945 | 0.600017269 | 0.628591873 |  |
| C1S | 0.759112691 | 0.751972528 | 0.627142748 |  |
| ADAMTS10 | 0.687070624 | 0.670913681 | 0.62694823 |  |
| EDNRA | 0.746767271 | 0.689974121 | 0.625960611 |  |
| EHD2 | 0.751550336 | 0.636988319 | 0.625590529 |  |
| FKBP7 | 0.7121234 | 0.636225165 | 0.624218893 |  |
| GLIS2 | 0.664099869 | 0.529464202 | 0.623493553 |  |
| TMEM204 | 0.759282424 | 0.712147604 | 0.622380834 |  |
| DOK5 | 0.670069781 | 0.64205589 | 0.622331447 |  |
| OLFML2B | 0.633511834 | 0.559880365 | 0.621681765 |  |
| CNRIP1 | 0.792369135 | 0.860749467 | 0.619366286 |  |
| MARVELD1 | 0.732496008 | 0.64166525 | 0.618905263 |  |
| CLMP | 0.791815301 | 0.782570892 | 0.618097916 |  |
| DCHS1 | 0.71435146 | 0.600659993 | 0.618027238 |  |
| FAM180A | 0.646584987 | 0.655483382 | 0.617845394 |  |
| BNC2 | 0.759210235 | 0.819135089 | 0.614297295 |  |
| KCNE4 | 0.728291486 | 0.714252844 | 0.613213344 |  |
| LTBP2 | 0.649148028 | 0.57741496 | 0.612653595 |  |
| IL1R1 | 0.672570908 | 0.638043474 | 0.612594244 |  |
| CCIN | 0.703298428 | 0.587039084 | 0.612581269 |  |
| AC093908.1 | 0.68503595 | 0.53998863 | 0.611573299 |  |
| PODN | 0.732982773 | 0.803679901 | 0.611422873 |  |
| MSC | 0.675812912 | 0.616023584 | 0.610824389 |  |
| MFAP5 | 0.623731335 | 0.614070068 | 0.609692526 |  |
| ASPN | 0.746256214 | 0.678922562 | 0.609490135 |  |
| LPAR4 | 0.656994091 | 0.69550074 | 0.603831221 |  |
| RNF144A | 0.628290206 | 0.518763944 | 0.602921086 |  |
| TMEM119 | 0.663167384 | 0.672471028 | 0.602455268 |  |
| AP001189.3 | 0.668362819 | 0.644599823 | 0.602290274 |  |
| GLI2 | 0.641011081 | 0.546774437 | 0.601696079 |  |
| CSDC2 | 0.755583902 | 0.672140895 | 0.601581713 |  |
| TIE1 | 0.682686466 | 0.635757812 | 0.595612824 |  |
| NDN | 0.765487298 | 0.783515403 | 0.592926916 |  |
| LOXL1 | 0.654809812 | 0.541361133 | 0.592303707 |  |
| NAP1L3 | 0.769563642 | 0.845063894 | 0.590957554 |  |
| ARHGAP31 | 0.661692165 | 0.611901839 | 0.590498564 |  |
| MAGI2-AS3 | 0.741905403 | 0.790032654 | 0.587460198 |  |
| RECK | 0.688900865 | 0.732331947 | 0.587304903 |  |
| DACT1 | 0.689453066 | 0.666007521 | 0.587025327 |  |
| HTRA1 | 0.640679818 | 0.510249521 | 0.5859503 |  |
| PTGIR | 0.724628672 | 0.722212544 | 0.58472646 |  |
| DNM3OS | 0.697228813 | 0.688340629 | 0.584134713 |  |
| ZCCHC24 | 0.792038132 | 0.886850716 | 0.582766889 |  |
| SEMA6B | 0.629357313 | 0.553841753 | 0.581505084 |  |
| PMP22 | 0.652463069 | 0.566669141 | 0.580826222 |  |
| ENG | 0.650852412 | 0.600452715 | 0.579555818 |  |
| AMPH | 0.694209636 | 0.604115404 | 0.579295742 |  |
| HSPA12B | 0.666176197 | 0.682081608 | 0.577316811 |  |
| MEDAG | 0.618872406 | 0.59073593 | 0.576474878 |  |
| GLI1 | 0.669531952 | 0.70485508 | 0.576346604 |  |
| GXYLT2 | 0.600778767 | 0.507547152 | 0.576075442 |  |
| BEND6 | 0.691565944 | 0.665928434 | 0.576056022 |  |
| THBS3 | 0.62188612 | 0.516064216 | 0.575851745 |  |
| ZBTB47 | 0.716245165 | 0.706083701 | 0.575731784 |  |
| DLC1 | 0.677764518 | 0.630142194 | 0.575525799 |  |
| SORCS2 | 0.60610461 | 0.532835029 | 0.575111835 |  |
| PTGER3 | 0.702183531 | 0.707412705 | 0.573773277 |  |
| CYP7B1 | 0.632929275 | 0.621858052 | 0.573034105 |  |
| SERPING1 | 0.697196665 | 0.706860031 | 0.572873655 |  |
| AFAP1 | 0.653863216 | 0.512762376 | 0.572625364 |  |
| GGT5 | 0.651024629 | 0.691036433 | 0.57120195 |  |
| BCL6B | 0.622405813 | 0.538969098 | 0.568119393 |  |
| DDR2 | 0.750763415 | 0.772648434 | 0.567793379 |  |
| GAS7 | 0.655380745 | 0.67943375 | 0.566579004 |  |
| NPR2 | 0.713525246 | 0.704275182 | 0.566504319 |  |
| LAMA2 | 0.662781187 | 0.691237806 | 0.565788976 |  |
| IGFBP5 | 0.658459685 | 0.630501825 | 0.56502988 |  |
| LHFPL6 | 0.699575584 | 0.802313621 | 0.562805715 |  |
| TNS2 | 0.703284629 | 0.752595115 | 0.560801088 |  |
| MFGE8 | 0.66280963 | 0.503800172 | 0.560110296 |  |
| HTR2A | 0.653267518 | 0.703032251 | 0.559805639 |  |
| ZFPM2 | 0.694160461 | 0.70379827 | 0.559613704 |  |
| SGIP1 | 0.620407064 | 0.503351949 | 0.559302805 |  |
| ZEB2 | 0.63377833 | 0.678630575 | 0.557324772 |  |
| ISM1 | 0.718340686 | 0.725583044 | 0.556786691 |  |
| SGCD | 0.7384787 | 0.742740708 | 0.555259125 |  |
| LRRN4CL | 0.727093839 | 0.807715914 | 0.555190608 |  |
| AC111197.1 | 0.68046309 | 0.721798252 | 0.553545602 |  |
| DIPK2B | 0.662345019 | 0.691375367 | 0.553134324 |  |
| COLEC12 | 0.647676207 | 0.688983562 | 0.552345242 |  |
| NHSL2 | 0.722741746 | 0.797744718 | 0.549765061 |  |
| LZTS1 | 0.606369258 | 0.576923165 | 0.548989023 |  |
| CYGB | 0.631790816 | 0.614949447 | 0.546580801 |  |
| ELN | 0.669358854 | 0.685215792 | 0.546368423 |  |
| ROR2 | 0.705863167 | 0.719534388 | 0.54631212 |  |
| ERG | 0.661963088 | 0.713413702 | 0.546022664 |  |
| FZD1 | 0.604370115 | 0.515428661 | 0.545987322 |  |
| MAP3K4-AS1 | 0.588097388 | 0.503006714 | 0.545872164 |  |
| AP001189.1 | 0.614910126 | 0.641429677 | 0.543926302 |  |
| ARHGEF15 | 0.632454599 | 0.62789029 | 0.543427383 |  |
| RAB3IL1 | 0.595339026 | 0.505424968 | 0.542822198 |  |
| TGFB1I1 | 0.801261812 | 0.808796778 | 0.541768438 |  |
| RHOJ | 0.72752447 | 0.768695366 | 0.54166483 |  |
| GUCY1B1 | 0.733852054 | 0.760884072 | 0.539729799 |  |
| STON1 | 0.753548302 | 0.817325141 | 0.538701733 |  |
| LINC01638 | 0.638546994 | 0.706115106 | 0.536282663 |  |
| TUBA1A | 0.742813793 | 0.744862059 | 0.536205228 |  |
| CD34 | 0.632164204 | 0.638358852 | 0.535778328 |  |
| GPR162 | 0.637928112 | 0.630968836 | 0.535376702 |  |
| RFTN2 | 0.59668138 | 0.573674932 | 0.534975364 |  |
| DLG4 | 0.615202919 | 0.590770096 | 0.534623478 |  |
| HMCN1 | 0.605389363 | 0.594551475 | 0.533999036 |  |
| C8orf48 | 0.60969203 | 0.529600207 | 0.533457844 |  |
| NOVA2 | 0.60756286 | 0.552733869 | 0.533221755 |  |
| PDGFRL | 0.595338995 | 0.554437314 | 0.531226046 |  |
| FAM43B | 0.644139815 | 0.740842914 | 0.530118984 |  |
| MXRA8 | 0.594432812 | 0.523148078 | 0.530090192 |  |
| IGFBP7 | 0.717569537 | 0.774840685 | 0.529107548 |  |
| FBLN1 | 0.602333772 | 0.687395377 | 0.528830927 |  |
| BHLHE22 | 0.656101131 | 0.800126668 | 0.528563124 |  |
| TNC | 0.670984708 | 0.566423173 | 0.528086123 |  |
| PKD2 | 0.686976558 | 0.678023576 | 0.527722621 |  |
| INAFM1 | 0.655441078 | 0.601845391 | 0.527281947 |  |
| FMO1 | 0.535345161 | 0.506700154 | 0.526989925 |  |
| ENTPD1 | 0.624341232 | 0.584866746 | 0.526717029 |  |
| ARHGEF17 | 0.641624363 | 0.529896194 | 0.526442573 |  |
| IGFBP4 | 0.654128258 | 0.714813086 | 0.525287406 |  |
| PABPC5 | 0.716985253 | 0.808167117 | 0.525110871 |  |
| HEG1 | 0.602144737 | 0.588978594 | 0.525031835 |  |
| ATP8B2 | 0.643177589 | 0.714419425 | 0.523996492 |  |
| PECAM1 | 0.614304093 | 0.629754886 | 0.523686019 |  |
| SVEP1 | 0.618551772 | 0.732232274 | 0.52365999 |  |
| SLC24A3 | 0.608586445 | 0.558525856 | 0.522968922 |  |
| PRICKLE1 | 0.612632306 | 0.643922155 | 0.522898148 |  |
| SPON1 | 0.627881602 | 0.674096622 | 0.522543247 |  |
| VIM | 0.603226464 | 0.55470532 | 0.522272753 |  |
| AP002414.2 | 0.572995612 | 0.502527715 | 0.522094526 |  |
| PRDM6 | 0.608829939 | 0.636583112 | 0.5212939 |  |
| NPR1 | 0.630579658 | 0.692352359 | 0.520485632 |  |
| ITGBL1 | 0.594539833 | 0.650987251 | 0.519462866 |  |
| MAP3K3 | 0.636298149 | 0.654174339 | 0.519366431 |  |
| COL14A1 | 0.61522576 | 0.741448627 | 0.518257287 |  |
| ZNF454 | 0.646032039 | 0.698178922 | 0.518203915 |  |
| PTGFR | 0.622278651 | 0.648319558 | 0.517645706 |  |
| PDLIM7 | 0.708947407 | 0.599721748 | 0.517489511 |  |
| SRPX | 0.608808419 | 0.71216394 | 0.516955299 |  |
| LINC00968 | 0.553894057 | 0.567245187 | 0.516950112 |  |
| SLIT3 | 0.608123475 | 0.70945759 | 0.516822846 |  |
| GDF6 | 0.544611096 | 0.551197853 | 0.516793756 |  |
| RHOQ | 0.634620101 | 0.568063937 | 0.516578323 |  |
| BOC | 0.688985678 | 0.826119705 | 0.515979779 |  |
| MFAP4 | 0.732748087 | 0.829495075 | 0.515933235 |  |
| C11orf96 | 0.642120385 | 0.628720618 | 0.514522827 |  |
| TSPAN4 | 0.583652129 | 0.57520074 | 0.514406516 |  |
| GUCY1A1 | 0.687661133 | 0.759471986 | 0.514149722 |  |
| CLIC4 | 0.69429067 | 0.63947592 | 0.514074174 |  |
| TTC28 | 0.654731669 | 0.711285841 | 0.513323436 |  |
| MMRN2 | 0.641380338 | 0.704374734 | 0.512551318 |  |
| OLFML3 | 0.618833162 | 0.584066818 | 0.511913082 |  |
| TEK | 0.610561002 | 0.640307083 | 0.511288198 |  |
| SEPTIN4 | 0.6209665 | 0.676943912 | 0.511269676 |  |
| BNC2-AS1 | 0.670566597 | 0.764175111 | 0.510968779 |  |
| DPYSL3 | 0.73420944 | 0.773639223 | 0.510265644 |  |
| PARVA | 0.710035456 | 0.71926928 | 0.510253508 |  |
| CDH5 | 0.571474596 | 0.510192067 | 0.509697299 |  |
| FBLN5 | 0.647745193 | 0.785284555 | 0.509495073 |  |
| AC092807.3 | 0.664996046 | 0.690641716 | 0.508742992 |  |
| CALHM2 | 0.600057207 | 0.591571549 | 0.50870876 |  |
| TCF21 | 0.634548674 | 0.702036904 | 0.507596481 |  |
| GJA4 | 0.586788589 | 0.587521236 | 0.507409482 |  |
| DKK3 | 0.615296779 | 0.501121751 | 0.506314721 |  |
| AC104117.3 | 0.601482049 | 0.6101988 | 0.505976524 |  |
| AKT3 | 0.673714928 | 0.681694133 | 0.505797192 |  |
| FAT4 | 0.593330421 | 0.545316022 | 0.505349127 |  |
| FHL3 | 0.625905903 | 0.518561318 | 0.503575503 |  |
| KIAA1755 | 0.592316355 | 0.577460749 | 0.503204598 |  |
| ARHGEF25 | 0.744109615 | 0.841400694 | 0.502802935 |  |
| AL049838.1 | 0.613758932 | 0.613098935 | 0.502541876 |  |
| FAM20C | 0.587787619 | 0.546683632 | 0.502253748 |  |
| GNG11 | 0.648807956 | 0.719620558 | 0.501173558 |  |
| NCAM2 | 0.576595235 | 0.50238252 | 0.500687838 |  |
